# Health consequences of exposure to e-waste: an updated systematic review

**DOI:** 10.1016/S2542-5196(21)00263-1

**Published:** 2021-12-08

**Authors:** Sarker M Parvez, Farjana Jahan, Marie-Noel Brune, Julia F Gorman, Musarrat J Rahman, David Carpenter, Zahir Islam, Mahbubur Rahman, Nirupam Aich, Luke D Knibbs, Peter D Sly

**Affiliations:** a Children's Health and Environment Program, Child Health Research Centre, The University of Queensland, South Brisbane, QLD, Australia; b Environmental Intervention Unit, Infectious Diseases Division, icddr,b, Dhaka, Bangladesh; c Department of Environment, Climate Change and Health, WHO, Geneva, Switzerland; d Department of International Health, Johns Hopkins Bloomberg School of Public Health, Baltimore, MD, USA; e School of Public Health, Environmental Health Sciences, University at Albany, Albany, NY, USA; f Department of Civil, Structural and Environmental Engineering, School of Engineering and Applied Sciences, University at Buffalo, The State University of New York, Buffalo, NY, USA; g School of Public Health, Faculty of Medicine and Health, The University of Sydney, Sydney, NSW, Australia

## Abstract

Electronic waste (e-waste) contains numerous chemicals harmful to human and ecological health. To update a 2013 review assessing adverse human health consequences of exposure to e-waste, we systematically reviewed studies reporting effects on humans related to e-waste exposure. We searched EMBASE, PsycNET, Web of Science, CINAHL, and PubMed for articles published between Dec 18, 2012, and Jan 28, 2020, restricting our search to publications in English. Of the 5645 records identified, we included 70 studies that met the preset criteria. People living in e-waste exposed regions had significantly elevated levels of heavy metals and persistent organic pollutants. Children and pregnant women were especially susceptible during the critical periods of exposure that detrimentally affect diverse biological systems and organs. Elevated toxic chemicals negatively impact on neonatal growth indices and hormone level alterations in e-waste exposed populations. We recorded possible connections between chronic exposure to e-waste and DNA lesions, telomere attrition, inhibited vaccine responsiveness, elevated oxidative stress, and altered immune function. The existence of various toxic chemicals in e-waste recycling areas impose plausible adverse health outcomes. Novel cost-effective methods for safe recycling operations need to be employed in e-waste sites to ensure the health and safety of vulnerable populations.

## Introduction

Exponential growth in the electrical and electronic industries to meet customer demand has correspondingly generated large waste flows.^1^, ^2^ Electronic and electrical waste (e-waste) can be defined as any “electrical or electronic equipment, which is waste, including all components, subassemblies and consumables, which are part of the equipment at the time the equipment becomes waste”.^3^ The Global E-waste Monitor estimated that 53·6 million metric tons (Mt) of e-waste were produced globally in 2019. This figure is projected to grow to 74·7 Mt by 2030. Asia generated largest quantity of e-waste in 2019 (24·9 Mt), followed by the Americas (13·1 Mt), Europe (12·0 Mt), Africa (2·9 Mt), and Oceania (0·7 Mt).^4^ An estimated 80% of e-waste from developed countries is illegally exported to low-income and middle-income countries (LMICs) including China, India, Nigeria, Brazil, Ghana, and Pakistan, where labour costs and disposal are cheap and laws are less stringent or poorly enforced.^5^

E-waste contains numerous toxic chemicals including metals such as lead, cadmium, mercury, and nickel, and organic compounds such as flame retardants, chlorofluorocarbons, polycyclic aromatic hydrocarbons (PAHs), polybrominated diphenyl ethers (PBDEs), and polychlorinated dibenzo-p-dioxins and furans (PCDD/Fs). E-waste recycling also recovers valuable materials including iron, aluminium, copper, silver, and rare earth metals but excessive exposure can be noxious.^6^, ^7^ These environmental contaminants pose severe threats to both the health of human beings and the environment.^8^

E-waste exposures occur in the informal and formal recycling sectors, and through contaminants that persist in the environment.^9^, ^10^ Most e-waste recycling occurs in the informal sector, often in unregulated work settings or as cottage industries in homes.^11^ Recovery of precious metals is inefficient, incomplete, and generally carried out without personal protective equipment or modern technology.^12^ Hazardous processes include open burning, manual dismantling, plastic chipping and melting, heating, and acid leaching, cyanide salt leaching, and mercury amalgamation.^9^, ^13^ Hazardous pollutants originating from such processes also contaminate ecosystems, leach into groundwater, contaminate food, and reduce air quality.^14^ Metal contaminants from e-waste are non-biodegradable, and can disturb the aquatic and terrestrial environment's ecological balance by persisting in the environment.^15^ Formal e-waste recycling, in which salvageable materials are safely removed with adequate worker and environmental protection, is expensive, limiting feasibility in LMICs. Although several LMICs have enacted legislation to restrain illegal import of e-waste into their countries, none of the legislation effectively regulates e-waste processing.^16^

E-waste exposures to people occur through multiple complex pathways. Type of exposure source, duration of exposure, and probable inhibitory, synergistic, or additive effects of multiple exposures are all factors that can influence health outcomes.^17^ It is difficult to ascertain the effect of exposure to a specific e-waste related compound or element in isolation. Inhabitants and workers living near e-waste recycling sites can be exposed through inhalation, ingestion, and dermal absorption when they come into physical contact with contaminated soil, dust, air, water, or food sources.^6^, ^10^ Residents living in the vicinity of e-waste recycling areas are at a particularly high risk of exposure. Exposure to contaminants associated with e-waste during gestation, infancy, or childhood can lead to obesity, asthma, or neurodevelopmental disorders.^18^ Adverse health outcomes associated with exposure to e-waste were reviewed in 2013 where 23 epidemiological studies were included from 2274 records published between Jan 1, 1965, and Dec 17, 2012.^17^ This Review updates evidence of the association between e-waste exposure and adverse human health consequences, following PRISMA guidelines.^19^

## Methods

### Search strategy and selection criteria

The complete review protocol, methods, and criteria were based on our previous systematic review.^17^ We searched Web of Science, EMBASE, PubMed, PsycNET, and CINAHL for articles published in English from Dec 18, 2012, to Jan 28, 2020 as previously described (full search terms are in the appendix).^17^ Our scope was limited to published epidemiological literatures that focused on exposure pathways in association with human health indicators (appendix). We excluded studies that reported outcomes in plants, animals, and in-vivo or in-vitro populations. We also excluded reviews, abstracts, editorials, correspondence, reports, book chapters, preface, commentary, and studies that did not report any human health outcome in relation to e-waste exposure. Our study was registered with PROSPERO, number CRD42021223833.

### Data analysis

After preliminary title and abstract screening, relevant articles were retrieved based on predetermined criteria. Two independent reviewers (SMP and FJ) assessed eligibility, with disagreements resolved by consensus. The reviewers developed a data extraction sheet by piloting and revised accordingly. Each reviewer extracted data independently using a standardised protocol based on the following characteristics: publication details, study design, location, sampling population, sample size, exposure, health outcomes, and effect sizes of association between exposure and health outcomes (appendix). Risk of bias was determined by focusing on methodological criteria described elsewhere.^17^

## Results

We identified 70 unique studies after full-text screening (figure). Most studies were done in China (n=66), followed by Vietnam (n=2), Ghana (n=1), and India (n=1). One study used a cohort design, and the rest were cross-sectional in nature. The most common reason for excluding studies was that health effects from e-waste exposures were not reported for the study population (figure). 11 Chinese articles with English abstracts were identified. During screening, one study was eligible for full-text review which we could not assess.

**Figure:**
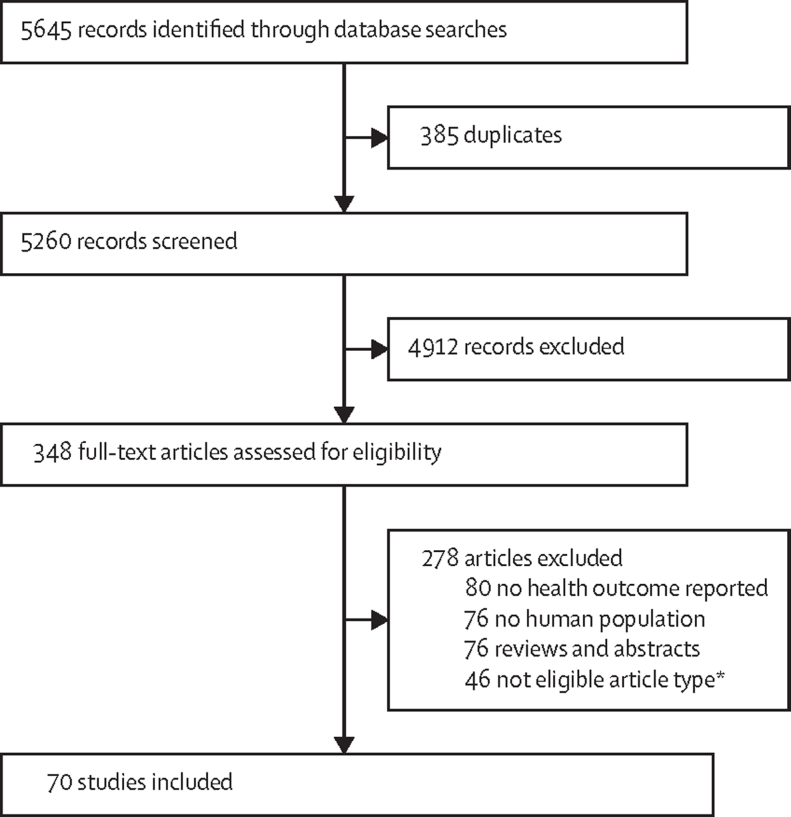
Study profile *Editorial, commentary, preface, news, correspondence, in-vitro experiments, case studies, reports, protocol articles, articles in Chinese, spotlights, chapters, and data articles.

Exposure to e-waste was associated with higher levels of many toxic chemicals and metals including: lead,^20^, ^21^, ^22^, ^23^, ^24^, ^25^, ^26^, ^27^, ^28^, ^29^, ^30^, ^31^, ^32^, ^33^, ^34^, ^35^, ^36^, ^37^, ^38^, ^39^, ^40^, ^41^, ^42^, ^43^, ^44^, ^45^, ^46^, ^47^, ^48^, ^49^, ^50^, ^51^, ^52^, ^53^, ^54^, ^55^ cadmium,^20^, ^22^, ^23^, ^26^, ^27^, ^28^, ^29^, ^31^, ^32^, ^33^, ^39^, ^45^, ^56^, ^57^ mercury,^32^ manganese,^26^, ^36^, ^39^, ^49^ chromium,^31^, ^39^, ^49^, ^58^ nickel,^49^, ^58^, ^59^ PAHs,^21^, ^48^, ^60^, ^61^, ^62^, ^63^, ^64^ PBDEs,^29^, ^35^, ^61^, ^65^, ^66^, ^67^, ^68^, ^69^, ^70^, ^71^, ^72^, ^73^, ^74^, ^75^, ^76^ polychlorinated bisphenols (PCBs),^35^, ^54^, ^67^, ^68^, ^69^, ^70^, ^72^, ^73^, ^74^, ^75^, ^77^, ^78^ dechlorane plus (DP),^77^, ^78^, ^79^ PCDD/Fs,^69^ new flame retardants (NFR),^72^, ^74^ bromophenols,^67^ perchlorate and thiocyanate,^80^ polybrominated biphenyls,^77^ phthalate esters,^81^ bisphenols,^82^, ^83^ and organophosphates.^84^ The chemical classification of e-waste components, sources and potential routes of exposure have been reported in the previous systematic review.^17^

Nine cross-sectional studies from China estimated the effect of e-waste-derived toxic chemicals on physical growth indicators. In general, increased toxicant levels were associated with poor foetal development in early life (table 1). Levels of PBDEs, PAHs, lead, and cadmium were significantly higher in exposed populations than they were in non-exposed individuals.^20^, ^21^, ^22^, ^23^, ^56^, ^60^, ^65^, ^66^ In two studies, neonatal head circumference, body-mass index, and Apgar1 scores were negatively correlated with PBDE concentrations detected in placenta and umbilical cord.^65^, ^66^ During pregnancy, higher urinary PAHs were associated with reduced birthweight, head circumference, body-mass index, and Apgar1 score.^60^ One study reported that blood PAHs were negatively associated with height and chest circumference in children aged 3–7 years.^21^ In three studies, blood lead exposure was associated with decreased child growth and development.^21^, ^22^, ^23^ A 10 ng/g increase in placental cadmium concentration was associated with a decrease of 205 g in weight and 0·44 cm in body length.^20^ Maternal urinary cadmium was associated with reduced birthweight, length, head circumference and Apgar scores in females neonates.^56^ Conversely, two studies found no effect of blood cadmium on child growth parameters (table 1).^22^, ^23^

**Table 1:** Growth and neurodevelopment effects from exposure to electronic waste

	**Exposure setting**	**Exposed population**	**Control population**	**Toxic chemicals**	**Health outcomes**
**Growth**					
Huo et al (2019)^60^	Cross-sectional: exposed area *vs* reference area, China	155 pregnant women (mean age 26·63 years)	102 pregnant women (mean age 27·68 years)	PAHs	Urinary ∑OHPAH=6·87 mg/g cre exposed *vs* 3·90 mg/g cre control (p<0·001); dominant metabolites=2-OHNap and 1-OHPyr. Elevated ∑OHPAHs associated with a decrease of 235 g in bodyweight (95% CI −452 to −17), decrease of 1·72 cm in head circumference (−2·96 to −0·48), decrease of 1·06 kg/m^2^ in BMI (−1·82 to −0·31), and decrease of 0·42 in Apgar1 score (−0·66 to −0·18; all p<0·05).
Li et al (2018)^65^	Cross-sectional: exposed town *vs* reference town, China	150 pregnant women (mean age 26·51 years)	150 pregnant women (mean age 28·43 years)	PBDEs	∑_14_PBDEs in umbilical cord=71·92 ng/g lw *vs* 15·52 ng/g lw (p<0·001) and negatively correlated with neonatal BMI (r=−0·20), Apgar1 score (r=−0·39), and head circumference (r=−0·37; all p<0·01).
Xu et al (2016)^20^	Cross-sectional: exposed town *vs* reference town, China	99 pregnant women (mean age 25·05 years)	86 pregnant women (mean age 27·96 years)	Lead and cadmium	Placental lead=498 ng/g wt *vs* 27 ng/g wt (p<0·01); cadmium=96·19 ng/g wt *vs* 12·65 ng/g wt (p<0·01). Shorter neonatal length in exposed =49·78 *vs* 50·30 cm (p<0·01). Cadmium negatively correlated with neonatal weight (B=−0·20) and length (B–0·44; both p<0·05). Lead was not statistically associated with birth outcomes (p>0·05). 32 differentially expressed proteins identified from 54 protein spots, FUM expression lower in exposed placenta (605 pg/g wt *vs* 1019 pg/g wt; p<0·05).
Xu et al (2015)^66^	Cross-sectional: exposed group *vs* reference group, China	69 pregnant women (mean age 26·4 years)	86 pregnant women (mean age 27·8 years)	PBDEs	Placental ∑PBDE=32·25 ng/g lw *vs* 5·13 ng/g lw; common congener=BDE −209, −28, −153, −183, −47, −99. Neonatal BMI=11·90 kg/m^2^*vs* 12·69 kg/m^2^, Apgar1 score=9·16 *vs* 10·0, and head circumference=33·52 cm *vs* 34·92 cm (all p<0·001). PBDE and BDE-47 negatively correlated with BMI, head circumference, and Apgar1 score, negative correlation between BDE-99 and BMI, BDE-28/153 and Apgar 1 score, and BDE-183 and BMI and Apgar1 score (all p<0·05).
Zhang et al (2018)^56^	Cross sectional: exposed town *vs* reference town, China	237 mother–neonate pairs (mean maternal age 26·29 years)	212 mother–neonate pairs (mean maternal age 28·52 years)	Cadmium	Maternal urinary cadmium with male neonates=1·38 μg/g cre *vs* 0·75 μg/g cre, urinary cadmium with female neonates=1·59 μg/g cre *vs* 0·76 μg/g cre (both p<0·001). Urinary cadmium negatively associated with birthweight (β=−0·16), length (β=−0·17), head circumference (β=−0·38), Apgar1 and Apgar5 score (β=−0·26 and β=−0·43) in female neonates (all p<0·05), in male neonates urinary cadmium negatively associated with Apgar1 score (β=−0·21; p<0·01).
Xu et al (2015)^21^	Cross-sectional: exposed town *vs* reference town, China	95 children aged 3–7 years	72 children aged 3–7 years	PAHs and lead	∑_16_PAHs in blood=68·53 μg/L *vs* 26·92 μg/L, ∑_7_ carcinogenic PAHs=60·27 μg/L *vs* 21·30 μg/L, blood lead 13·89 μg/dL *vs* 8·55 μg/dL (all p<0·01). Blood lead negatively correlated with child height (R_s_=−0·16; p<0·05); child height (β=−3·88) and chest circumference (β=−1·15) negatively associated with ∑_16_PAHs (p<0·05).
Yang et al (2013)^22^	Cross-sectional: e-waste processing area, China	246 kindergarten children aged 3–8 years	None	Lead and cadmium	Blood lead=7·30 μg/dL, blood cadmium=0·69 μg/L. Blood lead negatively associated with height (β=−0·10), weight (β=−0·14; both p<0·05), and positively associated with increase urinary excretion of DPD (mean 10·09 [SD 3·76 nmol/g]; p<0·01). No association between cadmium and bone, calcium metabolic biomarker (p>0·05).
Zeng et al (2019)^23^	Cross sectional: exposed town *vs* reference town, China	300 preschool children (mean age 4·66 years)	170 preschool children (mean age 4·34 years)	Lead, cadmium, chromium, and manganese	Blood lead=6·81 μg/dL *vs* 4·98 μg/dL, blood cadmium=0·66 μg/L *vs* 0·54 μg/L, PM_2·5_=57·73 μg/m^3^*vs* 40·53 μg/m^3^, elevated lead and cadmium in PM_2·5_ (data not shown; all p<0·05). Lower birth length, weight, BMI in exposed (all p<0·05), blood lead negatively associated with height (β=−0·06), weight (β=−0·12), head circumference (β=−0·12), and chest circumference (β=−0·10; all p<0·05). No association between cadmium, chromium, manganese with growth parameters (p>0·05).
**Neurodevelopment**					
Cai et al (2019)^24^	Cross-sectional: exposed town *vs* control town, China	358 preschool children (aged 3–6 years)	216 preschool children (aged 3–6 years)	Lead	Blood lead=4·88 μg/dL *vs* 3·47 μg/dL (p<0·001), serum cortisol=452 ng/mL *vs* 593 ng/mL (p<0·001), cortisol negatively associated with blood lead (B=−0·13, 95% CI −0·27 to −0·003; p<0·05). Elevated blood lead (>5 μg/dL) increased sensory integration difficulty scores (hearing, touch, body awareness, balance and motion, total sensory systems, r=0·10–0·18; p<0·05), scale for touch negatively correlated with serum cortisol levels (r=−0·16; p<0·05).
Liu et al (2015)^25^	Cross-sectional: exposed town *vs* control town, China	135 children (mean age 38 months)	149 children (mean age 39 months)	Lead	Blood lead=11·30 μg/dL *vs* 5·77 μg/dL, lower cognitive scores (100 *vs* 120) and language scores (100 *vs* 111; both p<0·001), no differences of DRD2 genotypes among exposed (p>0·05). Blood lead related to reduced cognitive (β=−0·19) and language scores (β=−0·72; both p<0·001). No association between DRD2 polymorphism and cognitive or language scores (p>0·05).
Liu et al (2014)^26^	Cross-sectional: e-waste disposal site, China	240 kindergarten children aged 3–7 years	None	Lead, cadmium, and manganese	Blood lead=7·33 μg/dL, blood cadmium=0·69 μg/L, blood manganese=17·98 μg/L, serum S100β=0·12 μg/L. ADHD prevalence=18·6% (higher prevalence in males than in females). Blood lead, cadmium, and manganese correlated with conduct problems and antisocial behaviour (data not shown), serum S100β positively correlated with blood lead (≥10 μg/dL, r=0·47) and some behavioural abnormalities (p<0·05).
Zhang et al (2015)^27^	Cross-sectional: e-waste recycling town, China	243 preschool children (aged 3–7 years)	None	Lead and cadmium	Blood lead=7·9 μg/dL, blood cadmium=0·95 μg/L, ADHD=12·8%. Positive correlations between blood lead and ADHD scores (inattentive, hyperactive/impulsive, and total scores, β=0·22–0·28; p<0·001). No correlation with blood cadmium. Elevated blood lead increased risk of ADHD (odds ratio 2·4, 95% CI 1·1 to 5·2).
Liu et al (2018)^28^	Cross-sectional: exposed group *vs* reference group, China	120 children (mean age 37·49 months)	138 children (mean age 38·80 months)	Lead and cadmium	Blood lead=11·30 μg/dL *vs* 5·77 μg/dL, blood cadmium=1·22 μg/L *vs* 0·72 μg/L (both p<0·001). Lower cognitive (100 *vs* 120) and language scores (99 *vs* 111), higher TSH, and higher FT_4_ (all p<0·01). Blood lead negatively correlated with cognitive scores (β=−1·57) and language scores (β=−0·80; both p<0·001) in mediation analysis, no correlation between blood cadmium and language or cognitive scores (p>0·05). FT_3_, FT_4_, and TSH did not mediate between lead and mental development.

PAH=polycyclic aromatic hydrocarbons. cre=creatinine. ∑OHPAH=total hydroxylated PAH. 2-OHNap=2-OHnaphthalene. 1-OHPyr=1-hydroxypyrene. BMI=body-mass index. Apgar1=Apgar score at 1 min. Apgar1=Apgar score at 5 mins. PBDE=polybrominated diphenyl ether. lw=lipid weight. wt=weight. FUM=fumarate hydratase. DPD=deoxypyridinoline. S100β=S100 calcium-binding protein β. DRD2=dopamine receptor-2. ADHD=attention-deficit hyperactivity disorder. TSH=thyroid stimulating hormone. FT_4_=free thyroxine. FT_3_=free triiodothyronine.

Five cross-sectional studies investigated the relationship between lead, cadmium, and manganese and neurodevelopmental outcomes among young children in China (table 1).^24^, ^25^, ^26^, ^27^, ^28^ Higher blood lead levels were associated with poorer neurodevelopmental outcomes, while results were less consistent for other metals. In two studies, children had lower cognitive and language scores that were negatively correlated with blood lead;^25^, ^28^ by contrast, no association was found with cadmium levels.^28^ Cai and colleagues^24^ found that children with higher blood lead levels had more sensory processing difficulties than did children with low blood lead levels. Behavioural abnormalities were found in children with higher blood levels of lead, cadmium, and manganese^26^ while in another study, children with high blood lead (≥10 μg/dL) had 2·4-times higher odds of attention-deficit hyperactivity disorder than did those with low lead exposure (table 1).^27^

13 studies investigated thyroid^29,67–73,79,80^ and sex hormones^30^, ^74^, ^85^ (table 2). Overall, exposure to e-waste-induced toxic chemicals disrupted thyroid function and had endocrine-disrupting effects on sex hormones. In two studies, thyroid hormones (THs) were measured among children aged 4–8 years.^29^, ^69^ No hormonal differences were found between children in the exposed and control groups and the association was not significant between toxicants (PCBs, PBDEs, and PCDD/Fs) and THs.^69^ By contrast, PBDEs were negatively associated with free triiodothyronine in the adjusted regression model; however, no correlation was found between THs and blood lead or cadmium levels.^29^ Xu and colleagues^69^ measured adrenocorticotropic hormone in young children which was positively correlated with serum PBDEs (table 2). In pregnant women, serum PCBs and PBDEs levels were negatively associated with thyroid-stimulating hormone (TSH)^71^ and total thyroxine,^71^ respectively, while DP concentration was positively associated with total triiodothyronine levels in maternal sera reported by Ben and colleagues.^79^ In three studies, serum PCBs were negatively associated with TSH,^67^ free triiodothyronine,^70^ and free thyroxine^70^, ^72^ among adults. However, no association was observed between THs and PCBs/hydroxylated PCBs,^73^ or PBDEs^70^ in other studies. By contrast, serum PBDEs were negatively associated with thyroxine; similarly, NFR disrupted effects on thyroxine-binding globulin and TSH in Chinese adults.^72^ In Vietnamese females, lower concentration of iodine was found where serum perchlorate or thiocyanate yielded no correlations with THs (table 2).^80^

**Table 2:** Hormonal and immunological function resulting from exposure to electronic waste

	**Exposure setting**	**Exposed population**	**Control population**	**Toxic chemicals**	**Health outcomes**
**Hormonal**					
Lv et al (2015)^68^	Cross-sectional: exposed villages *vs* reference village, China	64 pregnant women	10 pregnant women	PCBs and PBDEs	Serum ∑PCBs=26·2 ng/g lw *vs* 14·0 ng/g lw, ∑PBDE_8_=9·77 ng/g lw *vs* 4·80 ng/g lw, PCB-153=8·30 ng/g lw *vs* 3·33 ng/g lw (p=not shown). PCBs, PCB-153, and PCB-138 negatively associated with lower TSH (r=−0·34, r=−0·38, r=−0·45; all p<0·05); no association between PCBs/PBDEs and TT_3_, TT_4_, FT_3_, FT_4_ (p>0·05).
Ben et al (2014)^79^	Cross-sectional: exposed group (>20 years of living) *vs* control (<3 years), China	48 mother–infant pairs (mothers aged ≥18 years)	24 mother–infant pairs (mothers aged ≥18 years)	DPs	DP in maternal sera=13·5 ng/g lw *vs* 3·68 ng/g lw, placenta=4·27 ng/g lw *vs* 1·25 ng/g lw, cord blood=4·02 ng/g lw *vs* 2·03 ng/g lw (all p<0·05), strong correlations between DP concentrations in maternal sera and cord sera, maternal sera and placentas, placentas and cord sera (r>0·7; p<0·001). Lower TSH=1·76 μIU/mL *vs* 2·25 μIU/mL (p<0·05), no difference in TT_3_, TT_4_, FT_3_, FT_4_. DP levels associated with TT_3_ in maternal sera (syn-DP: r=0·37; anti-DP: r=0·36; p<0·05).
Zheng et al (2017)^71^	Cross-sectional: exposed group (>20 years of living) *vs* control (<3 years), China	48 paired mother–fetus	24 paired mother–fetus	PBDEs	PBDE in serum=19·3 ng/g lw *vs* 8·13 ng/g lw, umbilical cord=6·84 ng/g lw *vs* 4·47 ng/g lw, placental tissue=2·20 ng/g lw *vs* 1·06 ng/g lw (p<0·05), major congener=BDE-209 and BDE-153. Significant association between BDE-153 and TT_4_ in exposed group (β=−0·15, 95% CI −0·23 to −0·07, R^2^= 0·531; p<0·001).
Xu et al (2013)^61^	Cross-sectional: exposed city *vs* control city, China	101 pregnant women (mean age 26·20 years)	53 pregnant women (mean age 26·72 years)	PAHs and PBDEs	UCB ∑_16_PAHs=14·43 ppb *vs* 10·05 ppb, ∑PBDE=57·55 ng/g *vs* 8·23 ng/g lipid (both p<0·001). Increased placental IGF-1 and IGFBP-3 expression of mRNA (IGF-1: 0·23 *vs* 0·19 and IGFBP-3: 1·91 *vs* 0·68 (both p<0·05). Lower birthweight and Apgar score in exposed group. ∑PBDEs, ∑_4_ ring-PAHs and ∑_16_PAHs positively correlated with IGFBP-3 (β=0·44, β=0·34, and β=0·26, respectively; all p<0·01). BDE-154, BDE-209, and ∑_5_ ring-PAHs correlated with IGF-1 mRNA (β=0·23, β=0·24, and β=0·29, respectively; all p<0·05).
Xu et al (2014)^29^	Cross-sectional: e-waste area, China	162 children aged 4–6 years	None	PBDEs, lead, and cadmium	Serum PBDE=189·99 ng/g lipid, blood lead=14·53 μg/dL, blood cadmium=0·77 μg/L. Mean FT_3_=6·28 pmol/L, FT_4_=17·78 pmol/L, TSH=2·85 μIU/mL, IGF-1=510·79 ng/mL, IGFBP-3=60·97 ng/mL. ∑PBDEs negatively associated with FT_3_ (β=−0·19) and positively associated with TSH (β=0·27; both p<0·005). BDE-153 correlated with blood lead (β=0·19; p<0·05), no correlation between THs and blood lead or cadmium (p>0·05).
Xu et al (2014)^69^	Cross-sectional: exposed town *vs* control town, China	21 children aged 8 years	24 children aged 8 years	PCBs, PBDEs, and PCDD/Fs	Serum ∑PCBs=40·56 ng/g lipid *vs* 20·69 ng/g lipid, ∑PBDEs=32·09 ng/g lipid *vs* 8·43 ng/g lipid (both p<0·001), PCDD/F=206 pg/g lipid *vs* 160 pg/g lipid (p>0·05). Elevated mean of FT_3_, TT_3_, TT_4_, ACTH, cortisol, GH, and lower FT_4_, TSH, no difference among groups (p>0·05). ∑PBDEs positively associated with ACTH (r=0·61; p<0·05), cortisol positively associated with TSH (r=0·50) and GH levels (0·51; both p<0·05).
Eguchi et al (2014)^80^	Cross-sectional: exposed town *vs* non exposed rural site, Vietnam	83 local residents, aged 10–64 years	48 local residents, aged 10–64 years	Perchlorate (ClO_4_^−^) and thiocyanate (SCN^−^)	Serum perchlorate=0·116 ng/mL *vs* 0·086 ng/mL (p<0·05). Thiocyanate=2020 ng/mL, iodine=3·11 ng/mL, PEC=2·28 μmol/L, greater concentration among males (p<0·05). TT_3_=1·2 ng/mL *vs* 1·3 ng/mL, FT_3_=3·3 pg/mL *vs* 3·4 pg/mL (p<0·05), no correlation between THs and perchlorate/thiocyanate (p>0·05). Iodine significant positive predictor of FT_3_ (β=0·16), TT_3_ (β=0·19), and negative predictor of TSH (β=−0·45; all p<0·01) in males.
Eguchi et al (2015)^67^	Cross-sectional: exposed town *vs* non-exposed rural site, Vietnam	77 adult workers and residents (mean age 33 years)	34 adult workers and residents (mean age 37 years)	PCBs, OH-PCB, PBDEs, MeO-PBDE, OH-PBDE, and BPh	Serum PCBs=420 pg/g *vs* 290 pg/g, OH-PCBs=160 pg/g *vs* 82 pg/g, PBDEs=290 pg/g *vs* 230 pg/g, and BPhs=300 pg/g *vs* 200 pg/g (all p<0·05). FT_3_=3·3 pg/g *vs* 3·5 pg/g, TT_3_=1·2 pg/g *vs* 1·3 pg/g, TT_4_=78 pg/g *vs* 85 pg/g (all p<0·05), FT_4_=1·3 pg/g *vs* 1·2 pg/g, TSH=1·4 pg/g *vs* 1·5 pg/g (both p>0·05). Positive correlation between FT_4_, FT_3_, TT_3_, TT_4_ and PCBs/OH-PCB, and negative correlation between PCB and TSH in females (all p<0·05).
Xu et al (2015)^70^	Cross-sectional: exposed town *vs* control town, China	40 local residents, aged 15–65 years	15 local residents, aged 15–65 years	PCBs and PBDEs	Serum ∑PCBs=964 ng/g *vs* 68 ng/g (p<0·001), ∑PBDEs=139 ng/g *vs* 75 ng/g (p>0·05). FT_3_=4·72 pmol/L *vs* 5·64 pmol/L, FT_4_=14·98 pmol/L *vs* 18·67 pmol/L (both p<0·001), TSH=2·51 μIU/mL *vs* 1·80 μIU/mL (p>0·05). ∑PCBs negatively correlated with FT_3_ (r=−0·41) and FT_4_ (r=−0·39), no correlation between PBDEs and THs (p>0·05).
Guo et al (2019)^72^	Cross-sectional: exposed town *vs* control town, China	54 adult residents aged 26–75 years	58 adult residents aged 26–75 years	PCBs, PBDEs, and NFR	∑PCB=310 ng/g lipid *vs* 42 ng/g lipid, ∑PBDE=190 ng/g lipid *vs* 74 ng/g lipid, ∑NFR=350 ng/g lipid *vs* 110 ng/g lipid (all p<0·05). No mean difference of T_3_, T_4_, FT_3_, FT_4_, TSH among groups (p>0·05), TBG=18 μmol/L *vs* 20 μmol/L (p<0·05). PCB-28, 52, 101, 138, 153 negatively associated with FT_4_ (p<0·05), PBDEs negatively associated with T_4_(p<0·05). ∑NFR negatively associated with TSH (p<0·05) and TBG (p<0·05). Positive association between PBDE congener and T_3_ (BDE-85, BDE-99) and FT_3_ (BDE-47; all p<0·05).
Zheng et al (2017)^73^	Cross sectional: e-waste recycling workers, China	79 adult workers, aged 22–59 years	None	PBDEs, PCBs, and OH-PCB	Serum PCBs=2251 ng/g lipid, PBDEs=724 ng/g lipid, and OH-PCBs 418 ng/g lipid, no association between THs and PCBs/OH-PCBs (p>0·05), elevated T_3_ and T_4_ associated with certain PBDEs congeners (β=0·11–0·17; p<0·05). TH-regulated gene expression associated with certain PCB, OH-PCB, and mostly PBDE congeners (p<0·05)
Yan et al (2013)^30^	Cross-sectional: e-waste dismantling area, China	187 men aged 18–60 years	None	Lead	Blood lead=100·08 μg/L (≤30 years=98·55 μg/L, 31–45 years=100·23 μg/L, and 46–60 years=101·45 μg/L). FSH (≤30 years=5·64 mIU/mL, 31–45 years=11·51 mIU/mL, 46–60 years=15·32 mIU/mL), LH (≤30 years=4·59 mIU/mL, 31–45 years=4·90 mIU/mL, 46–60 years=5·96 mIU/mL), Tr (≤30 years=4823 mIU/mL, 31–45 years=4157 mIU/mL, 46–60 years=3562 mIU/mL). Blood lead associated with FSH (r=0·96), LH (r=0·92), and Tr levels (r=0·89; all p<0·01).
Guo et al (2018)^74^	Ecological study: exposed town *vs* control town, China	54 local residents, aged 26–75 years	58 local residents, aged 26–75 years	NFR, PCBs, and PBDEs	Serum ∑PCB=310 ng/g lipid *vs* 42 ng/g lipid, ∑PBDE=190 ng/g lipid *vs* 74 ng/g lipid, ∑NFR=350 ng/g lipid *vs* 110 ng/g lipid among exposed group (all p<0·05). Female FSH=12 mIU/mL *vs* 55 mIU/mL (p<0·05). NFR (TBB, DPa, DBDPE) and PBDE (BDE-153, 154, 183) negatively associated with female FSH, male Tr positively associated with NFR (TBECH, BTBPE, DPa) and PBDE congener (BDE-47, 100, 153, 183, 207; p<0·05).
Zhou et al (2013)^85^	Cross-sectional: exposed town *vs* reference town, China	46 parturient women (mean age 27·82 years)	44 parturient women (mean age 24·89 years)	Not assessed	Serum E2=2137 pg/mL *vs* 1549 pg/mL, umbilical cord E2=2758 pg/mL *vs* 2211 pg/mL, serum PROG=100 ng/mL *vs* 61 ng/mL, umbilical cord PROG=156 ng/mL *vs* 146 ng/mL (all p<0·05). mRNA of ERalpha, ERbeta increased in placenta and umbilical cord among exposed, mRNA of PROG decreased in placenta and umbilical cord among exposed (all p<0·05).
**Immunological**					
Cao et al (2018)^40^	Cross-sectional: exposed town *vs* reference town, China	62 preschool children aged 3–7 years	56 preschool children aged 3–7 years	Lead	Blood lead=5·06 μg/dL *vs* 3·60 μg/dL (p<0·001). Higher percentage of CD4^+^ Tcm and CD8^+^ Tcm cells among exposed (geometric mean=25·79% *vs* 21·43% and 0·89% *vs* 0·62%, respectively; p<0·001). No difference in serum cytokines (IL-2, IL-7, IL-15) among groups. Blood lead positively associated with CD4^+^ Tcm (β=0·49; p<0·05) and marginal change in CD8^+^ Tcm (p<0·05).
Huo et al (2019)^41^	Cross-sectional: exposed group *vs* reference group, China	132 preschool children aged 2–7 years	135 preschool children aged 2–7 years	Lead	Blood lead=6·51 μg/dL *vs* 4·41 μg/dL, erythrocyte lead=16·60 μg/dL *vs* 11·77 μg/dL (p<0·001). Reduced erythrocyte CD44 and CD58 expression (68·03% *vs* 76·15% and 40·76% *vs* 46·22%, respectively; p<0·01). Elevated erythrocyte lead associated with lower CD44 (B_Q4_ −5·44% [95% CI −9·11 to −1·73]) and CD58 (B_Q4_ −4·27% [−6·90 to −1·68]). Higher cytokines (IL-1β, IL12p70, IFN-γ, except IL-2). Elevated blood lead correlated with higher IL-12p70 (r_s_=0·20), IFN-γ (r_s_=0·22), and lower IL-2 (r_s_=−0·15), leukocyte count (r_s_=−0·12), lymphocyte ratio (r_s_=−0·16), LMR (r_s_=−0·18; all p<0·05).
Zhang et al (2016)^42^	Cross sectional: exposed town *vs* reference town, China	285 preschool children aged 3–7 years	126 preschool children aged 3–7 years	Lead	Blood lead=6·00 μg/dL *vs* 3·92 μg/dL, and lower NK cells (CD3^−^CD56^+^, CD3^−^CD56^bright^CD16^low/−^, and CD3CD56^dim^CD16^+^), increased platelets, IL-1β and lower IL-2, IL-27, MIP-1α, MIP-1β concentration in exposed (all p<0·05), negative association between CD3^−^CD56^bright^ CD16^low/−^ and blood lead (β=−0·182; p<0·05). Blood lead correlated with platelet, neutrophil, monocyte (R_s_=0·11, 0·14, and 0·12, respectively; p<0·05). IL-1β positively and IL-27 negatively associated with blood lead (R_s_=0·16 and −0·31; p<0·05).

PCB=polychlorinated bisphenol. PBDE=polybrominated diphenyl ether. lw=lipid weight. ∑PCBs=total PCB. ∑PBDE8=sum of eight congeners. TSH=thyroid stimulating hormone. TT3=total triiodothyronine. TT4=total thyroxine. FT_3_=free triiodothyronine. FT_4_=free thyroxine. DP=dechlorane plus. syn-DP=syn (or endo)-dechlorane plus. anti-DP=anti (or exo) dechlorane plus. PAH=polycyclic aromatic hydrocarbons. UCB=umbilical cord blood. ppb=parts per billion. IGF-1=insulin-like growth factor. IGFBP-3=IGF binding protein 3. TH=thyroid hormone. PCDD/F=polychlorinated dibenzo-p-dioxins and dibenzofurans. ACTH=adrenocorticotropic hormone. GH=growth hormone. PEC=perchlorate-equivalent concentrations. OH-PCB=hydroxylated PCB. MeO-PBDE=methoxylated PBDE. OH-PBDE=hydroxylated PBDE. BPh=bromophenols. FSH=follicle-stimulating hormone. LH=luteinising hormone. Tr=testosterone. NFR=new flame retardants. TBG=thyroxine-binding globulin. TBB=2-ethylhexyl 2,3,4,5-tetrabromobenzoate. DPa=dechlorane plus anti. DBDPE=1,2-bis(2,3,4,5,6-pentabromophenyl)ethane. TBECH=tetrabromoethylcyclohexane. BTBPE=1,2-bis(tribromophenoxy)-ethane. E2=oestradiol. PROG=progesterone. ERalpha=oestrogen receptor alpha. ERbeta=oestrogen receptor beta. CD4^+^Tcm=CD4^+^ central memory T cells. CD8^+^Tcm=CD8^+^ central memory T cells. B_Q4_=beta coefficient in quartile 4. IL=interleukin. IFN=interferon. LMR=lymphocyte-to-monocyte ratio. NK=natural killer. MIP=macrophage inflammatory protein.

Three studies investigated sex hormones in Chinese adults.^30^, ^74^, ^85^ Elevated serum oestradiol and progesterone were found in exposed pregnant women.^85^ Guo and colleagues^74^ observed disrupting effects of NFR with PBDE congeners on female follicle-stimulating hormone and male testosterone. Moreover, correlation between male sex hormones and blood lead level was somewhat significant, depending on age group assessed.^30^ In two studies,^29^, ^53^ serum insulin-like growth factor (IGF)–IGF-binding protein (IGFBP) were measured in children aged 4–7 years where the investigators did not find an association between IGF-1 and PBDEs^29^ or lead.^53^ However, one PBDE congener (BDE-209) was positively associated with IGFBP-3.^29^ Xu and colleagues^61^ reported that placental IGF-1 and IGFBP-3 were significantly higher in women exposed to e-waste. Exposure to PBDEs and PAHs in utero affects IGF-1 and IGFBP-3 mRNA levels in the placenta, which might have adverse effects on foetal growth and development (table 2).

Alteration of proinflammatory cytokines was observed among preschool children^41^, ^42^, ^64^ and local adult residents^75^ living in an e-waste recycling area when compared with reference sites in China. However, proangiogenic cytokines (RANTES and GROα) did not differ significantly between the two groups.^64^ Multiple studies detected that elevated lead exposure stimulates cytokine secretion, including IL-1β,^41^, ^42^ IL-27,^42^ 1L-12p70,^41^ and IFN-γ.^41^ Another study noted a potential association between IL-1β and urinary PAH exposure.^64^ Huo and colleagues^41^ estimated the effect of lead exposure on expression of erythrocyte adhesion molecules (CD44 and CD58) where reduced erythrocyte immunity was observed due to long-standing environmental lead contamination among preschool children. Cao and colleagues^40^ observed higher proportions of CD4^+^ central memory T cells in an e-waste recycling area where exposure to lead contributed to the increased percentages of peripheral CD4^+^ central memory T cells. Another study found that CR1 expression, which plays crucial roles in B-lymphocyte and T-lymphocyte immune responses, was depressed due to lead exposure.^43^ Two studies^42^, ^45^ identified lower natural killer cells among exposed children, in one study elevated lead levels resulted in lower percentages of natural killer cells^42^ while another study did not (table 2).^45^

Findings from two studies observed telomere aberration among e-waste-exposed pregnant women^57^ and local residents.^75^ The data suggested that telomere dysfunction is potentially induced through exposure to cadmium^57^ and persistent organic pollutants (POPs).^75^ Placental telomere attrition probably begins to occur at the cadmium concentration of 0·0294 mg/g.^57^ Upon proteomic analysis in the umbilical cord, the investigator found altered protective cell oxidative damage (CAT and GSTO1) and cell apoptosis (Cyt c) biomarkers associated following exposure to PBDEs.^65^ In four studies, the micronucleus rate was used to evaluate genotoxicity and was found to be significantly elevated among residents living near the e-waste disposal site.^35^, ^54^, ^75^, ^78^ However, He and colleagues^78^ revealed no correlation between POPs accumulation and micronucleus rate. RNA expression genes involved in ion binding, ion transport, immune regulation, apoptosis, and oxidoreductase activity were verified by quantitative fluorescence PCR where the number of genetic aberrations was higher in men than women.^35^ Multiple studies observed significantly decreased DNA methylation in populations living in e-waste exposed regions (table 3).^36^, ^59^, ^75^

**Table 3:** Genetic and oxidative changes resulting from exposure to electronic waste

	**Exposure setting**	**Exposed population**	**Control population**	**Toxic chemicals**	**Health outcomes**
**Genetic**					
Li et al (2018)^65^	Cross-sectional: exposed town *vs* reference town, China	150 pregnant women (mean age 26·51 years)	150 pregnant women (mean age 28·43 years)	PBDEs	Umbilical cord ∑_14_PBDEs=71·92 ng/g lw *vs* 15·52 ng/g lw (p<0·001). Lower expression of CAT=902 pg/g wt *vs* 1305 pg/g wt, GSTO1=526 pg/g wt *vs* 562 pg/g wt, Cyt c=389 pg/g wt *vs* 268 pg/g wt (all p<0·01). ∑_14_PBDEs, BDE-17, BDE-99, BDE-183 associated with decreased CAT expression (β=−0·31 to −0·10), GSTO1 decrease with BDE-153, BDE-190 (β=−0·20 to −0·16), BDE-99, BDE-190 increased Cyt c expression (β=0·16 to 0·19; all p<0·05).
Lin et al (2013)^57^	Cross-sectional: exposed town *vs* non-polluted town, China	227 healthy puerperae (mean age 26·45 years)	93 healthy puerperae (mean age 27·63 years)	Lead and cadmium	Placental cadmium=0·09 μg/g *vs* 0·02 μg/g (p<0·01), lead=1·25 μg/g *vs* 1·35 μg/g (p>0·05). Placental telomere length negatively correlated with cadmium (r=−0·14; p<0·05), no correlation between placental lead and telomere length (r=0·03; p>0·05). Positive correlation between mean TRF length and T/S ratio (R^2^=0·79; p<0·01). residence during pregnancy in exposed associated with telomere length (OR=2·0, 95% CI 0·07 to 0·60).
Zeng et al (2019)^36^	Cross sectional: exposed town *vs* reference town, China	101 pregnant women (mean age 27·3 years)	103 pregnant women (mean age 28·0 years)	Lead, cadmium, manganese, and chromium	Umbilical cord blood lead=7·34 μg/dL *vs* 3·07 μg/dL (p<0·001), no difference of umbilical cord blood cadmium, manganese, and chromium among groups (p>0·05). Methylation of BAI1 (cg25614253; 8% *vs* 7%, hyper-regulated), CTNNA2 (cg20208879; 62% *vs* 64%, hypo-regulated; all p<0·05), both correlated with umbilical cord blood lead (r=0·16 and r=−0·19; p<0·05). In adjusted regression, umbilical cord blood lead negatively associated with CTNNA2 (β=−1·20, 95% CI −2·13 to −0·26). No correlation between umbilical cord blood cadmium, manganese, chromium levels, and the methylation levels of two CpGs.
Huo et al (2014)^34^	Cross sectional: exposed town *vs* reference town, China	189 neonates and 319 children	84 neonatesand 185 children	Lead	Blood lead in neonates (2004–05: 10·50 μg/dL *vs* 7·79 μg/dL; 2006: 9·41 μg/dL *vs* 5·49 μg/dL), children (2004–05: 15·31 μg/dL *vs* 9·94 μg/dL; 2006: 13·17 μg/dL *vs* 10·04 μg/dL; all p<0·05). No difference of ALAD genotypes between groups (p>0·05), no significant differences between blood lead and ALAD-1/ALAD-1 or ALAD-1/ALAD-2 among newborns and children (all p>0·05).
Xu et al (2020)^37^	Cross-sectional: exposed town *vs* reference town, China	68 preschool children aged 3–7 years	48 preschool children aged 3–7 years	Lead and cadmium	Blood lead=5·29 μg/dL *vs* 3·63 μg/dL (p<0·001), urinary cadmium 1·52 μg/g *vs* 1·21 μg/g cre (p>0·05). Higher promoter methylation levels at cg02978827, position +14, and lower methylation at position +4 of Rb1 (all p<0·05), no difference of methylation in CASP8, MeCP2 among groups. Strong positive trend of MeCP2 promoter methylation with increasing lead (R^2^=0·709) and cadmium (R^2^=0·687), minimal negative trend of Rb1 (R^2^=0·014 and R^2^=0·015) and CASP8 (R^2^=0·001 and R^2^=0·002).
Li et al (2014)^35^	Cross-sectional: close proximity (≤5 km to e-waste recycling) *vs* remote group (<40 km), China	30 adult residents (mean age 41 years)	28 adult residents (mean age 33 years)	Calcium, copper, iron, lead, zinc, selenium, magnesium, and POPs	Lead=90·39 μg/L *vs* 68·40 μg/L, copper=17·34 μM *vs* 15·20 μM, MDA=1·29 *vs* 0·25 nmol/mL, PCBs=42·59 *vs* 10·14, PBDEs=23·05 *vs* 14·60, calcium=1·71 nM *vs* 1·82 nM, zinc=101 μM *vs* 127 μM (all p<0·05). Micronucleus=18·27% *vs* 7·32% (p<0·001). CD4^+^/CD8^+^ T cell ratios, CD4^+^CD25^nt/hi^CD127^lo^ regulatory T cell percentage, and CD95 expression higher in close proximity group (p>0·05). RNA expression genes: men detrimentally affected (p<0·05).
Yuan et al (2018)^75^	Cohort study: exposed town (e-waste disposal center) *vs* control town, China	3349 local residents	2606 local residents	PCBs, PBDEs, and lipid-standardised serum POP	Increased PCBs, PBDEs, ageing signal pathway (P53, Rb, P16^INK4a^, and P14^ARF^ in plasma), IL-6 and IL-10 (p<0·05, data not shown), increased TNF-α (p>0·05, data not shown) among exposed. Micronucleus=20·62% *vs* 7·21% (p<0·01), telomere loss=1·24% *vs* 0·10%, fragile telomere=2·76% *vs* 0·69%, decreased LINE-1 DNA methylation in exposed. PBDE-184 correlated with telomere shortening (r=−0·27; p<0·05). POP exposures associated with type 2 diabetes, autoimmune disorders, abnormal pregnancy, and foetal growth.
Li et al (2020)^59^	Experiment site (e-waste residents and former workers) *vs* reference site, China	23 local residents and 23 former workers, aged 30–50 years	45 residents aged 30–50 years	25 metals	Arsenic=17·24 ng/mL *vs* 15·42 ng/mL *vs* 10·84 ng/mL, nickel=4·01 ng/mL *vs* 4·76 ng/mL *vs* 1·95 ng/mL, silver=0·16 ng/mL *vs* 0·22 ng/mL *vs* 0·03 ng/mL, lanthanum=0·30 ng/mL *vs* 0·47 ng/mL *vs* 0·03 ng/mL, cerium=2·43 ng/mL *vs* 4·08 ng/mL *vs* 0·06 ng/mL (all p<0·05 between controls *vs* e-waste residents and controls *vs* former workers). Blood cerium negatively correlated with global DNA methylation among former workers (r=−0·51; p<0·05).
He et al (2015)^78^	Cross sectional: exposed town *vs* non-exposed town, China	23 adult residents (mean age 35 years)	25 adult residents (mean age 35 years)	PCBs, BDE, DP, HCB, HCH, and DDE	PCBs=149 ng/g lipid *vs* 35 ng/g lipid, DPs=8·14 ng/g lipid *vs* 1·96 ng/g lipid, BDE congeners=16·33 ng/g lipid *vs* 14·28 ng/g lipid (all p<0·05). Higher ROS activity (data not shown) and micronucleus rate (16·74% *vs* 7·8%) in exposed (both p<0·05), no correlation between POPs (PBDE/DP/PCB) and micronucleus rate (p<0·05). Expression of NEIL1/3, RPA3 downregulated, and E3 ligase RNF8 upregulated. Expression of CDC25A upregulated in males and downregulated in females among exposed (p<0·05).
Guo et al (2019)^72^	Cross-sectional: exposed town *vs* control town, China	54 local adult residents aged 26–75 years	58 local adult residents aged 26–75 years	PCBs, PBDEs, and NFR	∑PCB=310 ng/g lipid *vs* 42 ng/g lipid, ∑PBDE=190 ng/g lipid *vs* 74 ng/g lipid, ∑NFR=350 ng/g lipid *vs* 110 ng/g lipid; all p<0·05). Lower expression of TRα=14 × 10^−3^*vs* 29 × 10, TRβ=0·47 × 10^−3^*vs* 0·32 × 10^−3^, and higher expression of ID1=4·2 × 10^−3^*vs* 3·2 × 10^−3^ (all p<0·05). High PCBs, PBDEs and NFRs exposures decrease expression of TRα, and increase expression of ID1 (p<0·05).
**Oxidative damage**					
Ni et al (2014)^31^	Cross-sectional: exposed town *vs* control town, China	126 pregnant women (mean age 26·05)	75 pregnant women (mean age 25·45)	Lead, cadmium, chromium, and nickel	Umbilical cord blood lead=110 ng/mL *vs* 57 ng/mL, cadmium=2·50 ng/mL *vs* 0·33 ng/mL (p<0·001), no difference of nickel and chromium among groups (p>0·05). Umbilical cord blood 8-OHdG=162 ng/mL *vs* 154 ng/mL (p>0·05). 8-OHdG positively associated with cadmium (β=0·13, 95% CI 0·05 to 0·20), chromium (β=0·09, 95% CI 0·01 to 0·16), and nickel (β=0·21, 0·11 to 0·32; all p<0·05).
Zhou et al (2013)^85^	Cross-sectional: exposed town *vs* reference town, China	46 parturient women (mean age 27·82)	44 parturient women (mean age 24·89)	Not assessed	Increased MDA, suppressed SOD in maternal serum, umbilical cord serum, placentas, and umbilical cord among exposed (p<0·05). GPx decreased in placentas and umbilical cord in exposed (p<0·05). MDA, SOD, and GPx in maternal serum associated with umbilical cord serum (r=0·90, r=0·86, r=0·85; all p<0·01), MDA, SOD, GPx in placentas associated with umbilical cords (r=0·89, r=0·96, r=0·77; all p<0·01).
Xu et al (2018)^32^	Cross-sectional: e-waste recycling area, China	118 preschool children aged: 3–6 years	None	Lead, cadmium, and mercury	Blood lead=7·43 μg/dL, blood cadmium=0·72 μg/L, blood mercury=11·13 μg/L, median 8-OHdG=407·79 ng/g cre, median mRNA expression level of hOGG1=0·038. Elevated blood lead (quartiles 2–4) had higher 8-OHdG (β_Q2–Q4_=0·31–0·36; p<0·05) than low blood lead (quartile 1). No correlation between blood cadmium and 8-OHdG (p>0·05), elevated blood mercury (quartile 2) correlated with 8-OHdG than low blood mercury (β_Q2_=0·23; p<0·05).
Li et al (2013)^77^	Cross-sectional: exposed region *vs* reference region, China	23 rural residents (mean age 32·6 years)	28 rural residents (mean age 33·2 years)	PCBs, PBDEs, PBB, DP, HCB, β-HCH, and p,pʹ-DDE	PCBs=60·4 ng/g lipid *vs* 28·4 ng/g lipid, DP=9·0 ng/g lipid *vs* 2·8 ng/g lipid, PBB-153=0·55 ng/g lipid *vs* 0·25 ng/g lipid (all p<0·01). Increased ROS levels in WBC and NG, lower ROS in respiratory burst of NG among exposed (data not shown; p<0·001). Positive correlation between PCBs and ROS in WBC, NG (R=0·30 and R=0·31; p<0·05), inverse correlation between ROS in respiratory burst and PCBs (R=−0·45; p<0·01), no relation between ROS and PBDEs, DP, PBB153 (p>0·05).
Lu et al (2016)^62^	Cross-sectional: e-waste exposed town *vs* rural reference *vs* urban reference town, China	130 local residents aged 0·4–87 years	24 rural residents and 22 urban residents aged 0·4–87 years	PAH	Urinary ∑_10_OH-PAHs=25·4 μg/g cre *vs* 11·7 μg/g cre *vs* 10·9 μg/g cre, 8-OHdG=16·2 μg/g cre *vs* 12·3 μg/g cre *vs* 11·6 μg/g cre, MDA=47·9 μg/g cre *vs* 36·1 μg/g cre *vs* 31·3 μg/g cre (all p<0·05). 8-OHdG significantly increased with ∑_10_OH-PAHs (β=0·35, 95% CI 0·21 to 0·49) and individual OH-PAHs (β=0·10–0·35; p<0·05), urinary 1-PYR correlated with MDA (r=0·28; p<0·01) in exposed group.
Lu et al (2017)^84^	Cross-sectional: e-waste exposed town *vs* rural *vs* urban reference town, China	175 local residents aged 0·4–87 years	29 rural residents and 17 urban residents aged 0·4–87 years	Cl-mOPs and NCl-mOP metabolites	Urinary ∑Cl-mOPs=1·7 ng/mL *vs* 0·93 ng/mL *vs* 0·56 ng/mL (p<0·05), ∑NCl-mOPs=1·5 ng/mL *vs* 0·60 ng/mL (p<0·05 for exposed *vs* rural) *vs* 0·96 ng/mL, most abundant mOPs=BCEP (Cl-mOP) and DPHP (NCl-mOP) increased among exposed than rural reference (p<0·05). Significant association between 8-OHdG and BCEP (r=0·50), BCIPP (r=0·48), DBP (r=0·21), and DPHP (r=0·44) in exposed site (all p<0·05).
Yang et al (2015)^63^	Cross-sectional: e-waste recycling site, China	116 rural residents (mean age 36·9 years)	None	PAHs	1-HO-PYR=0·57 μg/g cre, HO-PHEs=2·2 μg/g cre, HO-FLU=5·0 μg/g cre, HO-BPs=7·0 μg/g cre, HO-NAPs=16·6 μg/g cre. Urinary MDA and 8-OHdG=74·7 μg/g cre and 185 μg/g cre. Positive association between MDA and hydroxy-PAH (1-HO-PYR [β=0·40], HO-PHEs [β=0·48], HO-FLUs [β=0·35], HO-BPs [β=0·48], HO-NAPs [β=0·28]; all p<0·001), no correlation between 8-OHdG and hydroxy-PAH (p>0·05).
Zhang et al (2019)^81^	Cross sectional: exposed *vs* reference village, China	124 local residents aged 0·4–87 years	22 local residents aged 0·4–87 years	PAEs	Urinary ∑mPAE=248 ng/mL *vs* reference (data not shown; p<0·05), higher mCMHP, mEHHP, mEHP, mMP, mEP in exposed group (p<0·05). Positive correlation between mECPP, mCMHP, mEHHP, mEHP, mCPP, mBP, miBP, mMP (8 of 11 mPAEs) and 8-OHdG (r=0·18–0·36; p<0·05).
Zhang et al (2019)^33^	Cross sectional: exposed town *vs* rural reference, China	139 local residents aged 0·4–87 years	26 local residents aged 0·4–87 years	Lead, cadmium, mercury, arsenic, cobalt, manganese, copper, zinc, thallium, tin, antimony, selenium, and aluminium	Urinary lead=4·98 ng/mL *vs* 1·23 ng/mL, cadmium=2·12 ng/mL *vs* 1·33 ng/mL, copper=22·2 ng/mL *vs* 16·9 ng/mL, antimony=0·20 ng/mL *vs* 0·11 ng/mL, arsenic=46·6 ng/mL *vs* 62·0 ng/mL (p<0·05). Urinary 8-OHdG positively correlated with all metals (except manganese and aluminium) in exposed group (r=0·324–0·710; p<0·01), high correlation coefficient between highly toxic arsenic, mercury, lead, cadmium and 8-OHdG (r=0·45–0·61; p<0·01).
Zhang et al (2016)^82^	Cross sectional: exposed villages *vs* rural reference village *vs* urban reference village, China	116 local residents aged 0·4–87 years	22 rural residents and 20 urban residents aged 0·4–87 years	BPA and 7 BPs	Urinary BPA=2·99 ng/mL *vs* 0·59 ng/mL *vs* 0·95 ng/mL (p<0·01), BPS=0·36 ng/mL *vs* 0·39 ng/mL (p>0·05 for exposed *vs* rural) *vs* 0·65 ng/mL, BPF=0·35 *vs* 0·09 (p<0·01 for exposed *vs* rural) *vs* 0·56 ng/mL, urinary 8-OHdG=8·00 ng/mL *vs* 6·84 ng/mL *vs* 7·31 ng/mL (p value not shown). 8-OHdG positively correlated with BPA (r=0·41) and BPS (r=0·39) in exposed (both p<0·001), no relation with BPF (p>0·05).

PBDE=polybrominated diphenyl ether. lw=lipid weight. wt=weight. CAT=catalase. GSTO1=glutathione S transferase omega-1. Cyt=cytochrome. BDE=brominated diphenyl ether. TRF=terminal restriction fragment. cre=creatinine. T/S ratio=telomere/single copy gene ratio. OR=odds ratio. BAI1=brain-specific angiogenesis inhibitor 1. CTNNA2=catenin cadherin-associated protein. ALAD=δ-aminolevulinic acid dehydratase. MDA=malondialdehyde. PCB=polychlorinated bisphenol. IL=interleukin. TNF=tumor necrosis factor. LINE-1=long interspersed nuclear element-1. POP=persistent organic pollutant. hOGG1=human repair enzyme 8-oxoguanine DNA glycosylase. HCB=hexachlorobenzene. HCH=hexachlorocyclohexane. ROS=reactive oxygen species. TR=TH receptor. IDI=iodothyronine deiodinase. 8-OHdG=8-hydroxy-2ʹ-deoxyguanosine. SOD=superoxide dismutase. GPx=glutathione peroxidase. WBC=white blood cell. NG=neutrophil granulocytes. PBB=polybrominated biphenyls. Cl-mOPs=chlorinated organophosphate metabolites. NCl-mOPs=non-chlorinated organophosphate metabolites. BCEP=bis(2-chloroethyl) phosphate. BCIPP=bis(1-chloro-2-propyl) phosphate. DBP=dibutyl phosphate. DPHP=diphenyl phosphate. PYR=pyrene. HO-PYR=hydroxypyrene. HO-PHEs=hydroxyphenanthrenes. HO-FLU=hydroxyfluorenes. HO-BPs=hydroxybiphenyls. HO-NAPs=hydroxynaphthalenes. PAH=polycyclic aromatic hydrocarbons. ∑OHPAH=total hydroxylated PAH. ∑mPAE=phthalate esters metabolites. mCMHP=mono-[(2-carboxymethyl)hexyl] phthalate. mEHHP=5mono-(2-ethyl-5-hydroxyhexyl) phthalate. mEHP=mono-2-ethylhexyl phthalate. mMP=mono-methyl phthalate. mEP=mono-ethyl phthalate. mECPP=mono-(2-ethyl-5-carboxypentyl) phthalate. mBP=mono-n-butyl phthalate. miBP=mono-(2-isobutyl) phthalate. mCPP=mono (3-carboxypropyl) phthalate. BP=bisphenol.

8-hydroxy-2′-deoxyguanosine (8-OHdG), a marker of DNA oxidative damage was measured as the outcome in eight studies.^31^, ^32^, ^33^, ^62^, ^63^, ^81^, ^82^, ^84^ Overall, the findings suggest that exposure to e-waste are associated with elevated oxidative stress. 8-OHdG concentrations were positively associated with elevated blood lead,^32^ hydroxylated PAHs,^62^ bis(2-chloro-isopropyl) phosphate, dibutyl phosphate, diphenyl phosphate,^84^ cadmium, chromium, nickel,^31^ phthalate esters,^81^ arsenic, mercury, lead, cadmium,^33^ and bisphenols-A (BPA)^82^ among e-waste exposed population. By contrast, some studies showed no statistical association between 8-OHdG and lead,^31^ cadmium,^32^ or hydroxy-PAHs.^63^ Li and colleagues^77^ found a possible linkage between PCBs and reactive oxygen species levels in immune cells which indicated higher oxidative stress in adults. Elevated malondialdehyde levels and decreases in both superoxide dismutase and glutathione peroxidase activities suggest that oxidative stress was higher among parturient women and their matching fetuses at an e-waste exposed site relative to referents (table 3).^85^

Respiratory outcomes were investigated in four studies in China where the data suggest that living in an e-waste exposed area might accelerate the respiratory symptoms of children aged 2–8 years.^38^, ^39^, ^86^, ^87^ In two studies, children exposed to e-waste had lower lung function levels including forced vital capacity (FVC) and forced expiratory volume in 1 s (FEV_1_).^38^, ^87^ Zeng and colleagues^38^ did not find a significant association between blood lead or cadmium, or the combined effects of blood lead and cadmium, and lung function, while each unit of haemoglobin (1 g/L) decline was associated with 5 mL decrease in FVC and 4 mL decrease in FEV_1_. However, elevated blood lead level (>5 μg/dL) was found as a risk factor for asthma (adjusted odds ratio 9·50, 95% CI 1·16–77·49).^39^ However, higher blood chromium and blood manganese in preschool children were associated with greater cough and wheeze, respectively.^39^ One study indicated that both birthweight and chest circumference might be good predictors for lung function levels which were positively associated.^87^ Zhang and colleagues^86^ found that severe PM_2·5_ pollution in an e-waste recycling area resulted in heavy individual PM_2·5_ chronic daily intake, and reduced Please add salivary agglutinin (SAG) level in exposed children. Furthermore, ambient PM_2·5_ pollution reduces airway antimicrobial activity by down-regulating saliva SAG levels, which might accelerate airway pathogen infection. However, no correlation between saliva SAG level and proinflammatory cytokines was found (table 4).^86^

**Table 4:** Respiratory, cardiovascular, and haematological changes resulting from exposure to electronic waste

	**Exposure setting**	**Exposed population**	**Control population**	**Toxic chemicals**	**Health outcomes**
**Respiratory**					
Zeng et al (2017)^38^	Cross sectional: exposed town *vs* reference town, China	100 preschool children aged 5–7 years	106 preschool children aged 5–7 years	Lead and cadmium	Blood lead=5·53 μg/dL *vs* 3·57 μg/dL (p<0·001), blood cadmium=0·58 μg/L *vs* 0·57 μg/L (p>0·05), lower Hb, HCT, higher platelet, thrombocytosis in exposed (all p<0·05). FVC=1·23 L *vs* 1·33 L, FEV_1_ =1·16 L *vs* 1·24 L (both p<0·01), FVC/FEV_1_=0·95% *vs* 0·96% (p>0·05). No association between blood lead, cadmium, platelet, and FVC, FEV_1_ (p>0·05). 1 g/L Hb decline associated with 5 mL and 4 mL decrease in FVC and FEV_1_, respectively (p<0·05).
Zeng et al (2017)^87^	Cross sectional: exposed town *vs* reference town, China	100 preschool children aged 5–7 years	106 preschool children aged 5–7 years	Not assessed	FVC=1·23 L *vs* 1·33 L, FEV_1_=1·16 L *vs* 1·24 L (both p<0·05). Birthweight=3·07 kg *vs* 3·25 kg, height=111·03 cm *vs* 112·56 cm, chest circumference=52·63 cm *vs* 53·42 cm (all p<0·05). Lung function associated with birthweight (FVC β=0·13, FEV_1_ β=0·15), chest circumference (FVC β=0·16, FEV_1_ β=0·15; all p<0·05).
Zeng et al (2016)^39^	Cross sectional: exposed town *vs* reference town, China	300 children aged 3–8 years	170 children aged3–8 years	Lead, cadmium, chromium, and manganese	Blood lead=6·24 μg/dL *vs* 4·75 μg/dL, blood cadmium=0·57 μg/L *vs* 0·50 μg/L, lead in PM_2·5_=153 ng/m^3^*vs* 80 ng/m^3^, cadmium in PM_2·5_=5·58 ng/m^3^*vs* 3·48 ng/m^3^ (all p<0·05), no differences in blood chromium and manganese (p>0·05). Increased cough, dyspnoea, phlegm, wheeze in exposed (p<0·05). Blood chromium associated with cough (AOR=1·91, 95% CI 1·17 to 3·13), Blood manganese associated with wheeze (AOR 2·91, 95% CI 1·09 to 7·78), elevated blood lead (>5 μg/dL) associated with asthma (AOR 9·50, 95% CI 1·16 to 77·49; all p<0·05).
Zhang et al (2019)^86^	Cross sectional: exposed town *vs* reference town, China	110 preschool children aged 2–7 years	112 preschool children aged 2–7 years	Not assessed	PM_2·5_=39·06 μg/m^3^*vs* 26·68 μg/m^3^, PM_2·5_ CDI=1·40 ng/kg per day *vs* 0·88 ng/kg per day (p<0·001). Saliva SAG=5·05 ng/mL *vs* 8·68 ng/mL, CDI negatively correlated with saliva SAG level (B=−1·21, 95% CI −2·29 to −0·13; p<0·05). Elevated white blood cells, neutrophils, monocytes, IL-8, and TNF-α in exposed (p<0·001), higher monocyte count associated with lower saliva SAG level (B=−6·25, 95% CI −11·76 to −0·75; p<0·05).
**Cardiovascular**					
Lu et al (2018)^47^	Cross-sectional: exposed town *vs* reference town, China	337 preschool children aged 3–7 years	253 preschool children aged 3–7 years	Lead	Blood lead=7·14 *vs* 3·91 μg/dL; p<0·001, elevated SBP, PP, TG, LDL/HDL and Tc/HDL ratio, lower HDL (all p<0·05). Higher Lp-PLA2=93·29 ng/mL *vs* 79·65 ng/mL, IL-6=10·00 pg/mL *vs* 1·61 pg/mL, IL-8=2·38 pg/mL *vs* 1·59 pg/mL, and TNF-α=2·36 pg/mL *vs* 1·86 pg/mL (all p<0·05). Elevated blood lead associated with higher Lp-PLA2, IL-6 (r_s_=0·20 and r_s_=0·59), (TG B=0·08, 95% CI 0·02 to 0·14) and lower HDL (B=−0·07, 95% CI −0·12 to −0·01), PP (B=−3·10, 95% CI −1·37 to −1·82; all p<0·05). Lp-PLA2 negatively associated with PP and HDL (B=−2·09 and B=−0·05; p<0·01).
Zheng et al (2019)^48^	Cross-sectional: exposed town *vs* reference town, China	105 preschool children aged 3–7 years	98 preschool children aged 3–7 years	Lead and PAHs	Blood lead=7·23 μg/dL *vs* 3·91 μg/dL and elevated urinary ∑OHPAHs, ∑OHNap and ∑OHFlu in exposed group (all p<0·05). Increased monocytes, neutrophils, leukocytes, serum S100A8/A9 and IL-6, IL12p70, IP-10, CD4^+^ T cell percentage in exposed. Elevated blood lead, urinary 2-OHNap and ∑OHFlu associated with higher levels of IL-6, IL12p70, IP-10, CD4^+^ T cell percentage, neutrophil and monocyte counts (all p<0·05).
Cong et al (2018)^88^	Cross-sectional: exposed town *vs* reference town (and non-native), China	228 preschool children aged 3–6 years	104 native and 91 non-native preschool children aged 3–6 years	PM_2·5_, PM_10_, SO_2_, NO_2_, CO, and O_3_	Higher concentrations of PM_2·5_, PM_10_, SO_2_, NO_2_, CO among exposed (data not shown, all p<0·001). Median heart rate=106 bpm *vs* 102 bpm and 100 bpm, plasma norepinephrine=4·42 nmol/L *vs* 3·88 nmol/L and 3·44 nmol/L (both p<0·01). Positive association between PM_2·5_, PM_10_, SO_2_, NO_2_and plasma norepinephrine, PM_2·5_, PM_10_, SO_2_, NO_2,_CO related to increase heart rate (p<0·05).
Gangwar et al (2019)^58^	Cross-sectional: exposed *vs* residential, commercial, and vehicular *vs* residential, India	28 local adult residents aged >18 years	50 adults from residential, commercial, and vehicular sites and 54 adults from residential sites (both groups aged >18 years)	PM_10_, lead, copper, zinc, nickel, and chromium	PM_10_=243 μg/m^3^*vs* 233 μg/m^3^*vs* 193 μg/m^3^. Elevated zinc, lead, chromium, nickel, copper in blood, and PM_10_ among exposed, positive correlation between blood and air heavy metals (r=0·58–0·98; p<0·05). HTN=68% *vs* 44% *vs* 32% (p<0·05), positive correlation between ambient PM_10_with mean SBP and DBP (r=0·62 and 0·67; p<0·05), elevated PM_10_ related to low SpO_2_ (r=−0·78; p<0·05). BMI and HTN positively correlated (data not shown).
Burns et al (2016)^89^	Cross-sectional: e-waste recycling activity, Ghana	57 e-waste recyclers, aged 18–61 years	None	Not assessed	High exposures to noise=43·5%, moderate to high levels of stress: mean PSS score=25 of 40. Positive correlation between noise and heart rate (ρ=0·46; p<0·001), 1 dB increase in noise associated with a 0·17 increase in heart rate (p<0·01).
**Haematological**					
Dai et al (2017)^43^	Cross-sectional: exposed town *vs* reference town, China	332 preschool children aged 2–6 years	152 preschool children aged 2–6 years	Lead	Blood lead=6·5 μg/dL *vs* 4·5 μg/dL, EPb=17·0 μg/dL *vs* 11·9 μg/dL (both p<0·001), lower median erythrocyte CR1 expression=6257 *vs* 8163 (p<0·01). Elevated erythrocyte lead and blood lead negatively associated with HCT, MCV, Hb, MCH, and MCHC (all p<0·05). High blood lead (>7·00) and erythrocyte lead (>18·6) associated with lower erythrocyte CR1 expression (β_Q4_=−0·16, 95% CI −0·32 to −0·008) and (β_Q4_=−0·19, 95% CI −0·35 to −0·03; both p<0·05).
Zeng et al (2018)^44^	Cross sectional: exposed town *vs* reference town, China	331 preschool children aged 3–7 years	135 preschool children aged 3–7 years	Lead	Blood lead=5·64 μg/dL *vs* 3·68 μg/dL (p<0·01), higher median PLT, PCT, MPV, P-LCR level among exposed (p<0·01). Positive correlation between blood lead and PLT (r=0·10), PCT (r=0·12), MPV (r=0·11), P-LCR (r=0·09), child residence in exposed associated with PLT (R^2^=0·07), PCT (R^2^=0·11), MPV (R^2^=0·03; all p<0·05).
Zhang et al (2017)^45^	Cross sectional: exposed group *vs* reference group, China	153 preschool children aged 3–7 years	141 preschool children aged 3–7 years	Lead and cadmium	Blood lead=10·34 μg/dL *vs* 8·30 μg/dL, blood cadmium=2·39 μg/L *vs* 1·79 μg/L (both p<0·001). Higher mean monocytes, eosinophils, neutrophils, basophils, and lower NK cells among exposed (all p<0·05). Blood lead escalated counts in monocytes (β=0·08), eosinophils (β=0·08), basophils (β=0·01), monocyte percentage (β=0·77) and decline neutrophils percentage (β=−4·15). Blood cadmium increase neutrophils percentage and counts (β=3·92 and 0·66; all p<0·05).
Dai et al (2019)^64^	Cross-sectional: exposed area *vs* reference area China	118 preschool children aged 2–7 years	121 preschool children aged 2–7 years	PAHs	Urinary ∑OH-PAHs=3·05 μg/mmol cre *vs* 1·76 μg/mmol cre, ∑OHNa=1·48 μg/mmol cre *vs* 0·75 μg/mmol cre, ∑OHPh=0·94 μg/mmol cre *vs* 0·62 μg/mmol cre (all p<0·001). Increased cytokines (IL-1β=0·43 pg/mL *vs* 0·25 pg/mL, IP-10=28·5 pg/mL *vs* 25·5 pg/mL), lymphocyte ratio, platelet count, PCT and PLR in exposed (all p<0·01). ∑OH-PAHs negatively associated with MPV, PDW, P-LCR, MPVP and positively associated with platelet count, PLR, ∑OHNa positively associated with IL-1β mediated through MPV, PDW, P-LCR, PLR (all p<0·05).
Xu et al (2015)^70^	Cross-sectional: exposed town *vs* control town, China	40 local residents aged 15–65 years	15 local residents aged 15–65 years	PCBs and PBDEs	∑PCBs=964 ng/g *vs* 68 ng/g (p<0·0001), ∑PBDEs 139 ng/g *vs* 76 ng/g (p>0·05). Lower monocyte, lymphocyte and higher neutrophil, Hb, platelets among exposed group (all p<0·05). ∑PCBs negatively correlated with monocyte (r=−0·67), lymphocyte (r=−0·38) and positively correlated with neutrophils (r= 0·58), Hb (r=0·35), ∑PBDEs positively correlated with WBC (r=0·34), Hb (r=0·34), and platelets (r=0·37).
Chen et al (2019)^46^	Experimental: exposed *vs* reference groups, China	158 hospitalised patients aged 4–85 years	109 hospitalised patients aged 4–85 years	Lead and cadmium	Blood lead=8·7 μg/dL *vs* 5·1 μg/dL (p<0·001), cadmium=2·1 μg/L *vs* 2·6 μg/L (p>0·05), RBC=4·5 × 10^3^ cell per μL *vs* 4·2 × 10^3^ cells per μL, Hb=137·0 g/dL *vs* 123·0 g/dL (both p<0·05), platelets (p>0·05). Blood lead positively correlated with blood cadmium (r=0·11; p<0·05). Positive correlation between blood lead and RBC (r=0·17), Hb (r=0·12, both p<0·05).

Hb=haemoglobin. HCT=haematocrit. FVC=forced vital capacity. FEV_1_=forced expiratory volume in 1 s. AOR=adjusted odds ratio. SAG=salivary agglutinin. CDI=chronic daily intake. IL=interleukin. TNF=tumour necrosis factor. SBP=systolic blood pressure. PP=pulse pressure. Tc=total cholesterol. TG=triglyceride. Lp-PLA2=lipoprotein-associated phospholipase A2. PAH=polycyclic aromatic hydrocarbon. ∑OHPAH =total hydroxylated polycyclic aromatic hydrocarbon. ∑OHNap=total hydroxylated naphthalene. ∑OHFlu=total hydroxylated fluorene. Bpm=beats per min. HTN=hypertension. PSS=perceived stress scale. SpO_2_=blood oxygen level. dB=decibel. DBP=diastolic blood pressure. BMI=body-mass index. EPb=erythrocyte lead. CR1=complement receptor. MCV=mean corpuscular volume. MCH= mean corpuscular haemoglobin. MCHC=mean corpuscular haemoglobin concentration. PLT=platelet count. PCT=plateletcrit. cre=creatinine. MPV=mean platelet volume. P-LCR=platelet large cell ratio. NK=natural killer. PLR=platelet count to lymphocyte count. PDW=platelet distribution width. MPVP=mean platelet volume to platelet count. ∑PBDEs=polybrominated diphenyl ether. RBC=red blood cells.

Six studies investigated haematological function as an outcome where lead, cadmium, PCBs, PBDEs, and PAHs were the primary chemical exposures with high concentrations among exposed preschool children,^43^, ^44^, ^45^, ^64^ local residents,^70^ and hospitalised patients.^46^ Two studies revealed that lead and PAH exposure were risk factors related to platelet indices among preschool children.^44^, ^64^ Similarly, blood and erythrocyte lead exposure was related to disadvantageous changes in red blood cells indices and haemoglobin in children from an e-waste recycling area.^43^ Zhang and colleagues^45^ concluded that the alteration of the number and percentage of innate immune cells were linked to higher lead and cadmium levels among e-waste exposed children. One study enrolled hospitalised patients from exposed and reference area where lead levels were correlated with elevated haematological parameters (red blood cells and haemoglobin levels).^46^ Native residents in an e-waste dismantling environment had increased body burden of PCBs and specific PBDE congeners which contributed to abnormal changes in haematological markers (table 4).^70^

Findings from five cross-sectional studies^47^, ^48^, ^58^, ^88^, ^89^ reported cardiovascular-related outcomes from e-waste exposure. Exposure to e-waste increased toxic chemical levels and concomitant abnormal measures of cardiovascular physiology. Three studies investigated cardiovascular risk in preschool children where vascular inflammation and lipid disorder were exacerbated by lead and PAH exposure^47^, ^48^ and air pollutants resulted in increased heart rate and plasma norepinephrine in participants from e-waste recycling areas.^88^ In India, e-waste burning contributed to severe air pollution, potentially explaining alarming levels of heavy metals in adult residents, which were associated with increased prevalence of cardiovascular morbidity, specifically hypertension.^58^ Moreover, noise exposure was associated with increased heart rate in Ghanaian adults (table 4).^89^

We identified three studies^49^, ^50^, ^51^ that included the effect of heavy metals from e-waste on immune system responsiveness of young children after vaccination. Significantly elevated blood lead and lower antibody titres of vaccines were reported among exposed children than those from the reference group in all three studies.^49^, ^50^, ^51^ Children chronically exposed to lead had suppressed antibody titres, indicating reduced immune responsiveness against diphtheria, pertussis, tetanus, Japanese encephalitis, polio,^49^ and hepatitis B.^49^, ^51^ However, no significant correlation was found between blood lead and anti-measles, mumps, or rubella antibody titres (table 5).^50^

**Table 5:** Vaccine, olfactory, reproductive, and other health effects from exposure to electronic waste

	**Exposure setting**	**Exposure setting**	**Control population**	**Toxic chemicals**	**Health outcomes**
**Vaccine**					
Lin et al (2017)^49^	Cross-sectional: exposed town *vs* reference town, China	157 preschool children aged 3–7 years	127 preschool children aged 3–7 years	Lead, zinc, arsenic, mercury, cadmium, chromium, copper, manganese, and selenium	Blood lead=9·43 μg/dL *vs* 6·79 μg/dL (p<0·001), elevated essential elements (manganese, copper, zinc, chromium; p<0·05) in exposed group. Lower antibody titres of diphtheria, pertussis, tetanus, Japanese encephalitis, polio, measles (all p<0·05), hepatitis B (p>0·05). Significant association between antibody titres and elevated lead (OR=0·31–0·45), copper (OR=0·47–0·60), and zinc (OR=0·48–0·56; all p<0·05).
Lin et al (2016)^50^	Cross-sectional: exposed town *vs* reference town, China	263 preschool children aged 2–7 years	115 preschool children aged 2–7 years	Lead	Blood lead=5·61 μg/dL *vs* 3·57 μg/dL (p<0·001). Lower antibody titres (median measles Ab=669 mIU/mL *vs* 1047 mIU/mL, mumps Ab=272 U/mL *vs* 492 U/mL, rubella Ab=37·08 IU/mL *vs* 66·50 IU/mL; all p<0·001). Anti-measles Ab titre positively associated with anti-mumps and rubella (r=0·16 and 0·37; p<0·01), Positive correlation between anti-mumps and anti-rubella Ab titres (r=0·17; p<0·01). No correlation between blood lead and anti-MMR Ab titres (p>0·05).
Xu et al (2015)^51^	Cross-sectional: exposed town *vs* reference town, China	301 kindergarten children (mean age 4·77 years)	289 kindergarten children (mean age 4·47 years)	Lead	Blood lead=6·76 μg/dL *vs* 6·05 μg/dL (p<0·01; 2011=8·76 μg/dL *vs* 7·89 μg/dL, 2012=5·83 μg/dL *vs* 4·61 μg/dL; both p<0·001), median HBsAb titres=1·04 s/co *vs* 4·06 s/co; p<0·001; 2011=0·83 s/co *vs* 4·64 s/co, 2012=1·31 s/co *vs* 3·80 s/co; p<0·001). HBsAb titres negatively associated with blood lead (β=−0·45 in 2011 and β=−0·37 in 2012; p<0·001).
**Auditory and olfactory**					
Liu et al (2018)^52^	Cross-sectional: exposed town *vs* reference town, China	146 preschool children aged 3–7 years	88 preschool children aged 3–7 years	Lead and cadmium	Blood lead=4·94 μg/dL *vs* 3·85 μg/dL (p<0·001), urinary cadmium=2·49 μg/g cre *vs* 1·80 μg/g cre (p>0·05). Hearing loss=28·8% *vs* 13·6% (p<0·001). Hearing loss for lead exposure: AOR=1·24 (95% CI 1·03 to 1·49).
Xu et al (2020)^37^	Cross-sectional: exposed town *vs* reference town, China	68 preschool children aged 3–7 years	48 preschool children aged 3–7 years	Lead and cadmium	Blood lead=5·29 *vs* 3·63 μg/dL; p<0·001, urinary cadmium=1·52 *vs* 1·21 μg/g cre; p>0·05, hearing loss (>25 dB)=50·0% *vs* 20·8%. AOR of lead for hearing loss=1·40 (95% CI 1·06 to 1·84).
Zhang et al (2017)^53^	Cross sectional: exposed town *vs* reference town, China	61 preschool children aged 4–7 years	57 preschool children aged 4–7 years	Lead	Blood lead=9·40 mg/dL *vs* 5·04 mg/dL, serum BDNF=35·91 ng/mL *vs* 28·10 ng/mL (both p<0·001), IGF-1=170 *vs* 154 ng/mL (p>0·05), BDNF positively correlated with blood lead (β=0·68; p<0·01). Lower item and source olfactory memory scores (at 15 min, 5 h, and 24 h) among exposed (p<0·01), and negatively correlated with blood lead (β=−0·29 to −0·16; p<0·05), BDNF (−0·23 to −0·19; p<0·05).
**Reproductive**					
Yu et al (2018)^76^	Exploratory: exposed town *vs* hospital bank, China	32 local adult men (mean age 38·7 years)	25 local adult men (mean age 36·0 years)	PBDE	BDE-28=5·02 pg/g *vs* 1·62 pg/g, BDE-47=6·75 pg/g *vs* 1·32 pg/g, BDE-153=7·36 pg/g *vs* 3·62 pg/g, (p<0·05) in semen, lower sperm count, sperm progressive motility among exposed, tail DNA (comet assay)=57·88% *vs* 33·55%, apoptosis rate (TUNEL assay)=32% *vs* 20% (all p<0·05). Inverse correlation between sperm concentration and count with BDE-47 (β=−0·29 and −0·40; p<0·05), sperm progressive motility ([A+B]%) and sperm viability negatively correlated with BDE-100 in dust (β=−0·36 and −0·11; p<0·05), positive correlation between BDE-28, BDE-47, BDE-153 and paired semen samples (r_s_=0·36–0·54; p<0·05).
Wang et al (2018)^54^	Cross-sectional: exposed town *vs* reference town, China	146 local male residents (mean age 35·8 years)	121 local male residents (mean age 34·9 years)	Lead, copper, zinc, iron, calcium, magnesium, selenium, and PCBs	Higher blood lead, PCBs, MDA, and lower calcium, magnesium, SOD, GSH among exposed (p<0·05, data not shown), MDA, lead, calcium, magnesium and DNA damage associated with the duration of exposure (p<0·05, data not shown). DNA damage in lymphocytes and spermatozoa (TDNA%, TM, OTM by comet assay), DNA aberrations (CA=8·01 *vs* 1·80% and CBMN=26·30% *vs* 4·52%) greater in exposed (all p<0·01). Semen volume=1·39 mL *vs* 2·52 mL, motility rate=45·01% *vs* 58·48% and reduced sperm count among exposed (p<0·05). Exposure duration, PCBs, MDA, and lead revealed risk factors of semen quality (all p<0·05). 13 genes expression of mRNA upregulated and 7 genes downregulated.
**Hepatic**					
Chen et al (2019)^46^	Cross-sectional: exposed *vs* reference groups, China	158 hospitalised patients aged 4–85 years	109 hospitalised patients aged 4–85 years	Lead and cadmium	Blood lead=8·7 μg/dL *vs* 5·1 μg/dL (p<0·001), cadmium=2·1 μg/L *vs* 2·6 μg/L (p>0·05), GGT=68·0 *vs* 26·0 (p<0·001), no difference of AST, ALT, AST/ALT, LDH among groups (p>0·05). Blood lead positively correlated with cadmium (0·117; p<0·05). Positive correlation between blood lead and ALT (r=0·111; p<0·05), Blood cadmium correlated with AST (r=0·22) and ALT (0·21; both p<0·001). Elevated blood lead (≥5 μg/dL) inducing abnormal liver function (AOR=1·94, 95% CI 1·00 to 3·73).
**Renal**					
Xu et al (2015)^70^	Cross-sectional: exposed town *vs* control town, China	40 local residents aged 15–65 years	15 local residents aged 15–65 years	PCB and PBDE	∑PCBs=964 ng/g *vs* 68 ng/g (p<0·001), ∑PBDEs 139 ng/g *vs* 75 ng/g (p>0·05). Serum creatinine=87·05 μmol/L *vs* 74·49 μmol/L, β2-MG=0·25 mg/L *vs* 0·18 mg/L (both p<0·001). ∑PCBs positively correlated with serum creatinine (r=0·40) and β2-MG (r=0·70; both p<0·01).
**Oral**					
Hou et al (2020)^55^	Cross-sectional: exposed town *vs* control town, China	357 preschool children aged 2·5–6 years	217 preschool children aged 2·5–6 years	Lead	Blood lead=4·86 μg/dL *vs* 3·47 μg/dL, IL-6=6·96 pg/mL *vs* 2·76 pg/mL, and TNF-α=6·51 pg/mL *vs* 1·29 pg/mL (all p<0·05). Lower salivary sialic acids=9·58 mg/dL *vs* 17·57 mg/dL (p<0·05), dental caries=62·5% *vs* 53·9% (p<0·05). Negative association between blood lead and salivary sialic acid (B=−5·59, 95% CI −9·62 to −1·55), in mediation analysis, inverse correlation between blood lead and salivary sialic acid through IL-6 (B=−0·95, 95% CI −1·70 to −0·20).
**Metabolic**					
Song et al (2019)^83^	Cross-sectional: exposed villages *vs* reference village, China	119 elderly residents aged 56–93 years	16 elderly residents aged 56–93 years	BPA and 6 alternatives	Serum BPA=3·2 ng/mL *vs* 2·8 ng/mL (p<0·05), dominant BPA alternatives=BPF (71%), BPAP (13%), BPAF (8%), BPS (7%). Abnormal FBG (<3·9 mmol/L or >6·1 mmol/L)=45% *vs* 31% (p<0·05), and associated with BPA (data not shown; p<0·05), high BPAF negatively correlated with low FBG (r=−0·30; p<0·001).

OR=odds ratio. Ab=antibody. MMR=measles, mumps, and rubella. HBsAb=hepatitis B surface antibody. cre=creatinine. AOR=adjusted OR. BDNF=brain derived neurotrophic factor. IGF-1=insulin-like growth factor. PBDE=polybrominated diphenyl ether. BDE=brominated diphenyl ether. TUNEL=TdT-mediated dUTP Nick-End Labelling. PCB=polychlorinated bisphenol. MDA=malondialdehyde. SOD=superoxide dismutase. GSH=glutathione. TDNA=DNA in the comet tail. CA=chromosome aberrations. TM=tail moment. OTM=olive tail moment. CBMN=cytokinesis-block micronucleus. GGT=gamma glutamyl transpeptidase. AST=aspartate aminotransferase. ALT=alanine aminotransferase. β2-MG=β2-microglobulin. BPAP=bisphenol AP. BPAF=bisphenol AF. BPS=bisphenol S. FBG=fasting blood glucose.

In three studies,^37^, ^52^, ^53^ hearing function was estimated concerning lead and cadmium exposures among preschool children. Exposed children had a higher prevalence of hearing loss than did reference children.^37^, ^52^ Lead concentration was significantly higher among exposed children than the reference group^37^, ^52^, ^53^ while no difference was found in urinary cadmium.^37^, ^52^ Two studies found hearing loss was more likely due to lead exposure (adjusted odds ratio 1·24, 95% CI 1·03–1·49 and 1·40, 1·06–1·84).^37^, ^52^ Zhang and colleagues^53^ observed lower olfactory memory scores after odour exposure among children exposed to e-waste, which were negatively correlated with blood lead and serum brain-derived neurotrophic factor levels. Two studies identified that lower semen quality aggravated DNA damage (by comet assay) in e-waste exposed individuals compared with individuals from a reference site.^54^, ^76^ Semen quality parameters were negatively correlated with PBDE congeners in semen samples^76^ and exposure duration from e-waste has a strong association with genomic instability among adult men (table 5).^54^

Chen and colleagues^46^ showed elevated hepatic parameters in patients from the exposed group compared with reference groups. Both blood lead and cadmium were positively associated with alanine aminotransferase. Likewise, elevated blood lead (≥5 μg/dL) induced abnormal liver function (adjusted odds ratio 1·94, 95% CI 1·00–3·73). Elevated serum creatinine and urinary β2-MG, which are clinical renal function indicators, were significantly higher among the exposed adult residents than the control group and they were positively correlated with serum PCBs.^70^ Increased levels of dental caries in deciduous teeth (62·5% *vs* 53·9%) and concomitantly lower salivary sialic acids were found in exposed preschool children when compared with preschool children from a reference town. Higher blood lead was adversely associated with salivary sialic acid levels indicating weakened oral anti-inflammatory ability.^55^ Elderly individuals living in e-waste recycling areas had elevated BPA levels, correlating with e-waste dismantling activities indicating that BPA exposure is associated with abnormal fasting blood glucose (ie, hyperglycaemia and hypoglycaemia; table 5).^83^

## Discussion

The current systematic review is an update of our previous systematic review conducted in 2013 and contains new evidence regarding the health effects from e-waste exposures. The 2013 review contained 23 articles over a span of 47 years. Since then, 70 studies were published in 7 years. Most studies followed an ecological or retrospective cohort design that collected cases from exposure and referral sites. Ten studies were conducted in recycling sites and did not contain reference sites.

The toxic chemicals in e-waste can have a significant adverse impact on health of people living in exposed areas, particularly during sensitive windows of development such as pregnancy and childhood. Endocrine-disrupting chemicals (EDCs) such as phenols, phthalates, parabens, flame retardants, and heavy metals have potentially played adverse health impacts on human reproduction and development.^90^ EDC exposures modulate various physiological processes in pregnant women, exposing the developing fetus to maternal nutritional, chemical, and environmental stressors. Such early life exposure could compromise the early developmental processes and predispose the fetus to adverse health risks later in life.^91^ Moreover, the cumulative effect of EDC exposure is a considerable concern while humans are exposed to a multitude of EDCs at varying doses that might have additive, synergistic, or adverse biological effects.^92^, ^93^ We provide evidence of adverse birth outcomes associated with heavy metals and organic chemicals.^20^, ^56^, ^60^, ^65^, ^66^ Previous studies have also shown that early exposure to heavy metals and organic pollutants in utero can lead to foetal growth retardation.^94^, ^95^ Significant associations between PCBs and PBDEs and maternal serum THs indicate links between maternal health and infant development. Since the chemical structure of PCBs and PBDEs is similar to that of THs, these chemicals can disrupt the thyroid endocrine system,^73^ altered TH homoeostasis and induce neurotoxicity.^96^ Across multiple studies, we found a consistent association between the effects of e-waste exposure and placental transfer of toxic chemicals, bioaccumulation of chemicals, DNA methylation, sex hormone homoeostasis, and oxidative damage in pregnant women. EDC exposures are associated with changes in the gestational endocrine milieu, including altered levels of sex steroids,^97^ altered developmental trajectory of developing fetuses via epigenetic modifications,^98^ modulation of immune system, altered inflammatory cytokine milieu to favour a proinflammatory state,^99^ and might also affect circulating inflammation markers (interleukin-6, interleukin-10, c-reactive protein, and tumour necrosis factor-α).^100^ A consistent theme in the literature more broadly is that exposures in early life can potentiate detrimental effects and resulting in acute infections, morbidity, or even death in infancy and childhood, plus chronic conditions might deleteriously influence health trajectories in later life.^101^ However, we have found some conflicting findings; for example the effect of cadmium exposure on child growth and development. As cadmium accumulates with age, a plausible explanation is that children might be too young to manifest bone problems resulting from cadmium exposure.^22^

Children are particularly vulnerable compared with adults to environmental exposures due to additional exposure routes (eg, breastfeeding, placental exposure, and frequent hand-to-mouth behaviours), their higher basal metabolic rate and immature systems that might be unable to handle and excrete some toxic materials efficiently.^12^ Compared with adults, children also have more time to develop diseases that could be triggered by toxic chemicals in childhood and can evolve through multiple stages and years.^102^ Some studies we reviewed suggest extensive toxicological effects in multiple organ systems among children, including growth and neurodevelopment, endocrine, respiratory, cardiovascular, haematological, immune, and genetic dysfunction related to e-waste exposures. However, consistency of association could not be assessed due to diverse variations in chemical exposure and associated outcomes.^103^ Also, several risk factors such as age, nutritional status, and predisposing conditions could influence the impact of toxic chemicals on health outcomes to some extent. However, the overall body of research suggests that children who are still growing and developing have a substantial risk of harm, from individual chemicals derived from e-waste.^17^, ^104^

Our review has identified adverse health outcomes in relation to e-waste exposure in addition to those identified in the previous review. A few studies have identified a link between chemical exposure from e-waste and suppressed immune response,^49^, ^50^, ^51^ hearing loss,^37^, ^52^ altered hepatic and renal function,^46^, ^70^ decline in oral anti-inflammatory ability,^55^ and abnormal FBG.^83^

One of the strengths of the current systematic review is that most studies relied on biomarkers, which provide an objective measure of chemical exposure (ie, the internal dose). However, other methodological issues were identified in the previous review that remain unchanged in the current review. First, the current review did not contain any longitudinal, prospective studies that establish any temporality of associations. Lack of long-term studies also affects data on diseases that have long latency periods. Second, we did not include any studies exploring dose–response relationships. Third, the sample sizes in the studies included in the current review were only 45–590 people; these low sample sizes are a source of continuing concern. However, given that so many studies identified an association between toxicants and adverse health outcomes within such small sample sizes, the effect sizes of the associations are probably substantial. Fourth, many studies did not adjust for confounders, which reduces our ability to draw inferences between exposure with health outcome.^105^ For example, BPA exposure is associated with obesity but BPA is found in less nutritious and calorie-dense packaged foods that can also increase the risk of obesity. If the consumption of packaged foods is not adjusted, BPA exposure could be assumed to be a risk factor for oxidative damage or adverse metabolic outcomes where consumption packaged foods might likely be driving these adverse health outcomes.^106^ Another example is socioeconomic background as a confounding factor. Children from lower socioeconomic backgrounds are more vulnerable to adverse cognitive health outcomes than are those from higher socioeconomic backgrounds.^107^ The current review contains multiple studies where the exposed and control groups were chosen from non-random populations. It is possible that socioeconomic background factors are driving some of the adverse health consequences and not the exposure to harmful toxicants. Fifth, there are periods of heightened vulnerability for the chemically exposed populations that can overestimate the health effects.^105^ Sixth, most of the studies in the current review explored health outcomes related to isolated exposures. We do not know the cumulative and interactive effects of exposure to chemical mixtures, possibly owing to a high cost for multiple measurements.^105^ Also, no studies in this Review measure the attributable risk from different exposures which could mask the contribution of potentially predominant route of toxicant exposure such as dietary intake. Finally, more than 90% of the studies in this Review were undertaken in China, which limits the generalisability of findings. Future studies should therefore include longitudinal designs, methodologies that allow for exploration of exposure and dose-response relationships, larger sample sizes, adjustments for confounders, random sampling of study populations, and an exploration of health effects in relation to increasing and decreasing levels of vulnerability and chemical mixtures in addition to isolated chemical exposures. Furthermore, future studies can also sample toxicants from multiple sources to gauge the extent to which e-waste contributes to toxicity levels relative to other sources. Most importantly, future studies need to be conducted among more diverse populations in different countries across the world.

Addressing e-waste is in alignment with multiple UN Sustainable Development Goals pertaining to environmental and human health protection (targets 3.9, 6.1, and 6.3), reducing adverse environment impacts of cities (target 11.6), sound management of e-waste in accordance with agreed international frameworks (target 12.4), and protection of labour rights along with economic growth for vulnerable populations (targets 8.3 and 8.8). Alongside the Sustainable Development Goals, other initiatives that continue to address e-waste at an international policy level. For example, the Basel Convention regulates the transboundary movement of hazardous wastes, including e-waste, and obliges countries to ensure safe management and disposal of e-waste and step focuses on scientifically developing a globally accepted standard for e-waste refurbishment and recycling. WHO's Initiative on E-waste and Child Health aims to increase access to evidence, knowledge, and awareness of the health impacts of e-waste, improve health sector capacity to identify risks and track progress of good e-waste policies and interventions that protect public health. Furthermore, initiatives within the context of developed countries are moving towards circular economy strategies that are increasingly focusing on aspects of upscale design and production aspects rather than curative aspects of e-waste management.^4^ These initiatives include emphasising consumer and producer-end responsibilities such as improving recycling habits and designing eco-friendly products.

Although 78 countries have identified policies, legislation, or regulation governing e-waste, these are not usually legally binding, and—where they are legally binding—enforcement is often a challenge.^4^ Ultimately, creating and enforcing policies to prevent the proliferation of e-waste is not nearly enough. In LMICs countries, policies and intervention focusing on curative strategies are imperative for tackling the proliferation of e-waste, both domestic and imported. Further initiatives need to explore cost-effective methods and appropriate technologies based on chemical toxicity for safe recycling operations, including metal recovery and improvement of disposal systems. Such approach should consider the economic benefits of value recovery processes while ensuring the health and safety of populations that depend on informal e-waste recycling for their livelihoods and survival.

## Declaration of interests

We declare no competing interests.
