# Supplementary material for: Health consequences of exposure to e-waste: an updated systematic review
Source: Lancet Planet Health. 2021 Dec 8;5(12):e905–20. doi: 10.1016/S2542-5196(21)00263-1 (PMC8674120; doi:10.1016/S2542-5196(21)00263-1)
Supplement: Supplementary appendix [file mmc1.pdf]

THE LANCET
Planetary Health

Supplementary appendix

This appendix formed part of the original submission and has been peer reviewed. We post it as supplied by the authors.

Supplement to: Parvez SM, Jahan F, Brune M-N, et al. Health consequences of exposure to e-waste: an updated systematic review. *Lancet Planet Health* 2021; **5:** e904–19.

Supplemental material: Consequences of exposure to e-waste: an updated systematic review:

Sarker M. Parvez[1,2], Farjana Jahan[2], Marie-Noel Brune[3], Julia F. Gorman[1], Musarrat J. Rahman[2], David Carpenter[4], Zahir Islam[1], Mahbubur Rahman[2], Nirupam Aich[5], Luke D. Knibbs[6], Peter D. Sly[1]

[1]Children's Health and Environment Program, Child Health Research Centre, The University of Queensland, Australia
[2]Environmental Intervention Unit, Infectious Diseases Division, icddr,b, Dhaka, Bangladesh
[3]Department of Environment, Climate Change and Health, World Health Organization, Geneva, Switzerland
[4] School of Public Health, Environmental Health Sciences, University at Albany, NY, USA
[5]Department of Civil, Structural and Environmental Engineering, School of Engineering and Applied Sciences, University at Buffalo, The State University of New York, Buffalo, NY, USA
[6] School of Public Health, Faculty of Medicine, The University of Queensland, Australia

Section/topic	#	Checklist item	Reported on page #
**TITLE**			
Title	1	Identify the report as a systematic review, meta-analysis, or both.	1
**ABSTRACT**			
Structured summary	2	Provide a structured summary including, as applicable: background; objectives; data sources; study eligibility criteria, participants, and interventions; study appraisal and synthesis methods; results; limitations; conclusions and implications of key findings; systematic review registration number.	2
**INTRODUCTION**			
Rationale	3	Describe the rationale for the review in the context of what is already known.	3
Objectives	4	Provide an explicit statement of questions being addressed with reference to participants, interventions, comparisons, outcomes, and study design (PICOS).	4 Introduction paragraph 4<br>Supplementary material page 5, 8-9
**METHODS**			
Protocol and registration	5	Indicate if a review protocol exists, if and where it can be accessed (e.g., Web address), and, if available, provide registration information including registration number.	2, Methods section (summary)
Eligibility criteria	6	Specify study characteristics (e.g., PICOS, length of follow-up) and report characteristics (e.g., years considered, language, publication status) used as criteria for eligibility, giving rationale.	5<br>Methods (search strategy and selection criteria)<br>Supplementary material page 5
Information sources	7	Describe all information sources (e.g., databases with dates of coverage, contact with study authors to identify additional studies) in the search and date last searched.	5<br>Method (Search strategy and selection criteria)
Search	8	Present full electronic search strategy for at least one database, including any limits used, such that it could be repeated.	Supplementary material page 5
Study selection	9	State the process for selecting studies (i.e., screening, eligibility, included in systematic review, and, if applicable, included in the meta-analysis).	5<br>Method (search strategy

			and selection criteria)
Data collection process	10	Describe method of data extraction from reports (e.g., piloted forms, independently, in duplicate) and any processes for obtaining and confirming data from investigators.	5 Method (data analysis)<br>Supplementary material (page 10)
Data items	11	List and define all variables for which data were sought (e.g., PICOS, funding sources) and any assumptions and simplifications made.	Supplementary material page 6-9
Risk of bias in individual studies	12	Describe methods used for assessing risk of bias of individual studies (including specification of whether this was done at the study or outcome level), and how this information is to be used in any data synthesis.	5 Method (data analysis)
Summary measures	13	State the principal summary measures (e.g., risk ratio, difference in means).	
Synthesis of results	14	Describe the methods of handling data and combining results of studies, if done, including measures of consistency (e.g., I^2) for each meta-analysis.	

Section/topic	#	Checklist item	Reported on page #
Risk of bias across studies	15	Specify any assessment of risk of bias that may affect the cumulative evidence (e.g., publication bias, selective reporting within studies).	
Additional analyses	16	Describe methods of additional analyses (e.g., sensitivity or subgroup analyses, meta-regression), if done, indicating which were pre-specified.	
**RESULTS**			
Study selection	17	Give numbers of studies screened, assessed for eligibility, and included in the review, with reasons for exclusions at each stage, ideally with a flow diagram.	5, Results<br>Paragraph 1<br>Figure 1
Study characteristics	18	For each study, present characteristics for which data were extracted (e.g., study size, PICOS, follow-up period) and provide the citations.	14-27, results<br>Table 1-5
Risk of bias within studies	19	Present data on risk of bias of each study and, if available, any outcome level assessment (see item 12).	Supplementary material<br>Table S1
Results of individual studies	20	For all outcomes considered (benefits or harms), present, for each study: (a) simple summary data for each intervention group (b) effect estimates and confidence intervals, ideally with a forest plot.	
Synthesis of results	21	Present results of each meta-analysis done, including confidence intervals and measures of consistency.	

Risk of bias across studies	22	Present results of any assessment of risk of bias across studies (see Item 15).	Results
Additional analysis	23	Give results of additional analyses, if done (e.g., sensitivity or subgroup analyses, meta-regression [see Item 16]).	
**DISCUSSION**			
Summary of evidence	24	Summarize the main findings including the strength of evidence for each main outcome; consider their relevance to key groups (e.g., healthcare providers, users, and policy makers).	10, Discussion Paragraph 1
Limitations	25	Discuss limitations at study and outcome level (e.g., risk of bias), and at review-level (e.g., incomplete retrieval of identified research, reporting bias).	11, Discussion Paragraph 5
Conclusions	26	Provide a general interpretation of the results in the context of other evidence, and implications for future research.	12, Discussion Paragraph 6-7
**FUNDING**			
Funding	27	Describe sources of funding for the systematic review and other support (e.g., supply of data); role of funders for the systematic review.	Contained within acknowledgement and financial disclosure section

From: Moher D, Liberati A, Tetzlaff J, Altman DG, The PRISMA Group (2009). Preferred Reporting Items for Systematic Reviews and Meta-Analyses: The PRISMA Statement. PLoS Med 6(7): e1000097. doi:10.1371/journal.pmed1000097

For more information, visit:**www.prisma-statement.org**.

Review protocol

Methods of the review

The study will be conducted according to the PRISMA statement

Data sources

5 electronic databases (PubMed, EMBASE, Web of Science, CINAHL, PsycNET) searched with the assistance of librarians.

Search terms

(e-waste OR electronic waste OR WEEE) AND (health OR development* OR mental OR education* OR behavior* OR learning OR psychological OR psychiatric* OR environment* OR exposure* OR food OR fish OR human breastmilk)

Database	Search group	Search terms
Pubmed		(e-waste OR electronic waste OR WEEE OR (Waste Electrical and Electronic Equipment)) AND (health OR development* OR mental OR education* OR behavior* OR learning OR psychological OR psychiatric* OR environment* OR exposure* OR food OR fish OR human breastmilk OR Milk, Human)
EMBASE		((('e waste'/exp OR 'e waste' OR electronic) AND (waste/exp OR waste)) OR weee) AND (health/exp OR health OR development* OR mental OR education* OR behavior* OR learning/exp OR learning OR psychological OR psychiatric* OR environment* OR exposure* OR food/exp OR food OR fish/exp OR fish OR human/exp OR human) AND breastmilk)
Web of Science		Topic=((e-waste OR electronic waste OR WEEE) AND (health OR development* OR mental OR education* OR behavior* OR learning OR psychological OR psychiatric* OR environment* OR exposure* OR food OR fish OR human breastmilk))
CINAHL		(e-waste OR electronic waste OR WEEE) AND (health OR development* OR mental OR education* OR behavior* OR learning OR psychological OR psychiatric* OR environment* OR exposure* OR food OR fish OR human breastmilk)
Psycnet		(e-waste OR electronic waste OR WEEE) AND (health OR development* OR mental OR education* OR behavior* OR learning OR psychological OR psychiatric* OR environment* OR exposure* OR food OR fish OR human breastmilk)

Inclusion/exclusion criteria

Inclusion criteria

Studies were included if they were published in a peer-reviewed journal and reported an association between exposure to waste electrical and electronic equipment (WEEE)/electronic waste (e-waste) and health, violence and criminal behavior, and educational outcomes.

Question of interest

Are individuals exposed to WEEE/e-waste at higher risk of developing health (mental and physical), education, aggression, crime and violence related problems compared with those who are not exposed?

Population

General population, children, adolescents or adults. Non-representative samples were also considered including occupational exposure groups.

Intervention/exposure

Definition of WEEE/e-waste exposure should follow European Union and Basel Convention definitions.

WEEE/E-waste Definition as per European Union and Basel Convention

**Basel Convention:**<br>Basel Convention covers all discarded / disposed materials that possess hazardous characteristics as well as all wastes considered hazardous on a national basis. Annex VIII, refers to E-waste, which is considered hazardous under Art. 1, para. 1(a) of the Convention:<br><br>**A1010:** Metal wastes and waste consisting of alloys of any of the following:<br>• Antimony<br>• Arsenic<br>• Beryllium<br>• Cadmium<br>• Lead<br>• Mercury<br>• Selenium<br>• Tellurium<br>• Thallium<br><br>**A1020:** Waste having as constituents or contaminants, excluding metal waste in massive form, any of the following:<br>• Antimony; antimony compounds<br>• Beryllium; beryllium compounds<br>• Cadmium; cadmium compounds<br>• Lead; lead compounds<br>• Selenium; selenium compounds<br>• Tellurium; tellurium compounds<br><br>**A1030:** Wastes having as constituents or contaminants any of the following:<br>• Arsenic; arsenic compounds<br>• Mercury; mercury compounds<br>• Thallium; thallium compounds<br><br>**A1090:** Ashes from the incineration of insulated copper wire<br><br>**A1150:** Precious metal ash from incineration of printed circuit boards not included on list B<br><br>**A1170:** Unsorted waste batteries excluding mixtures of only list B batteries. Waste batteries not<br><br>specified on list B containing Annex I constituents to an extent to render them hazardous<br><br>**A1180:** Waste electrical and electronic assemblies or scrap containing components such as accumulators and other batteries included on list A, mercury-switches, glass from cathode-ray tubes and other activated glass and PCB-capacitors, or contaminated with Annex I constituents (e.g., cadmium, mercury, lead, polychlorinated biphenyl) to an extent that they possess any ofthe characteristics contained in Annex III. Annex IX, contains the mirror entry, B1110 Electrical and Electronic assemblies given below.<br><br>• Electronic assemblies consisting only of metals or alloys<br>• Waste electrical and electronic assemblies or scrap (including printed circuit boards) not containing components such as accumulators and other batteries included on List A, mercury-switches, glass from cathode-ray tubes and other activated glass and

PCB-capacitors, or not contaminated with Annex 1.

A1190: Waste metal cables coated or insulated with plastics containing or contaminated with coal tar, PCB1, lead, cadmium, other organohalogen compounds or other Annex I constituents to an extent that they exhibit Annex III characteristics.

A2010: Glass waste from cathode-ray tubes and other activated glasses

WEEE Directive (EU, 2002a):

"Electrical or electronic equipment which is waste including all components, subassemblies and consumables, which are part of the product at the time of discarding."

Directive 75/442/EEC, Article 1(a) defines "waste" as "any substance or object which the holder disposes of or is required to dispose of pursuant to the provisions of national law in force."

(a) 'electrical and electronic equipment' or 'EEE' means equipment which is dependent on electrical currents or electromagnetic fields in order to work properly and equipment for the generation, transfer and measurement of such current and fields falling under the categories set out in Annex IA to Directive 2002/96/EC (WEEE) and designed for use with a voltage rating not exceeding 1000 volts for alternating current and 1500 volts for direct current

Annex IA
Categories of electrical and electronic equipment covered by this Directive

1. Large household appliances
2. Small household appliances
3. IT and telecommunications equipment
4. Consumer equipment
5. Lighting equipment
6. Electrical and electronic tools (with the exception of large-scale stationary industrial tools)
7. Toys, leisure and sports equipment
8. Medical devices (with the exception of all implanted and infected products)
9. Monitoring and control instruments
10. Automatic dispensers

Annex IB
List of products, which fall under the categories of Annex IA are given below.

1. Large household appliances

- Large cooling appliances
- Refrigerators
- Freezers
- Other large appliances used for refrigeration, conservation and storage of food
- Washing machines
- Clothes dryers
- Dish washing machines
- Cooking
- Electric hot plates
- Microwaves
- Other large appliances used for cooking and other processing of food
- Electric heating appliances
- Electric radiators
- Other fanning, exhaust ventilation and conditioning equipment

2. Small household appliances

- Vacuum cleaners
- Carpet sweepers
- Other appliances for cleaning
- Appliances used for sewing, knitting, weaving and other processing for textiles
- Iron and other appliances for ironing, mangling and other care of clothing
- Toasters

• Fryers
• Grinders, coffee machines and equipment for opening or sealing containers or packages
• Electric knives
• Appliances for hair-cutting, hair drying, tooth brushing, shaving, massage and other body care appliances
• Clocks, watches and equipment for the purpose of measuring indicating or registering time Scales.

3. IT and telecommunications equipment

• Centralized data processing
• Mainframes
• Minicomputers
• Printer units
• Personal computing:
• Personal computers (CPU, mouse, screen and keyboard included)
• Laptop computer (CPU, mouse, screen and keyboard included)
• Notebook computers
• Notepad computers
• Printers
• Copying equipment
• Electrical and electronic typewriters
• Pocket and desk calculators
• And other products and equipment for the collection, storage, processing, presentation or communication of information by electronic means
• User terminals and systems
• Facsimile
• Telex
• Telephones
• Pay telephones
• Cordless telephones
• Cellular telephones
• Answering systems
• And other products or equipment of transmitting sound, images or other information by telecommunications

4. Consumer equipment

• Radio sets
• Television sets
• Video cameras
• Video recorders
• Hi-fi recorders
• Audio amplifiers
• Musical instruments
• Other products or equipment for the purpose of recording or reproducing sound or image, including signals or other technologies for the distribution of sound and image than by telecommunications

5. Lighting equipment

• Luminaries for fluorescent lamps with the exception of luminaries in households
• Straight fluorescent lamps
• Compact fluorescent lamps
• High intensity discharge lamps, including pressure sodium lamps and metal lamps
• Low pressure sodium lamps
• Other lighting or equipment for the purpose of spreading or controlling light with the exception of filament bulbs

6. Electrical and electronic tools (with the exception large-scale stationary industrial tools)

• Drills
• Saws
• Sewing machines

- Equipment for turning, milling, sanding, grinding, sawing, cutting, shearing, drilling, making, holes, punching, folding, bending or similar processing of wood, metal and other materials
- Tools for riveting, nailing or screwing or removing rivets, nails, screws or similar uses
- Tools for welding, soldering or similar use
- Equipment for spraying, spreading, dispersing or other treatment of liquid or gaseous substances by other means
- Tools for mowing or other gardening activities

7. Toys, leisure and sports equipment

- Electric trains or car racing sets
- Hand-held video game consoles
- Video games
- Computers for biking, diving, running, rowing, etc.
- Sports equipment with electric or electronic components
- Coin slot machines

8. Medical devices (with the exception of all implanted and infected products)

- Radiotherapy equipment
- Cardiology
- Dialysis
- Pulmonary ventilators
- Nuclear medicine
- Laboratory equipment for *in-vitro* diagnosis
- Analysers
- Freezers
- Fertilization tests
- Other appliances for detecting, preventing, monitoring, treating, alleviating illness, injury or disability

9. Monitoring and control instruments

- Smoke detector
- Heating regulators
- Thermostats
- Measuring, weighing or adjusting appliances for household or as laboratory equipment
- Other monitoring and control instruments used in industrial installations (e.g.in control panels)

10. Automatic dispensers

- Automatic dispensers for hot drinks
- Automatic dispensers for hot or cold bottles or cans
- Automatic dispensers for solid products
- Automatic dispensers for money
- All appliances which deliver automatically all kind of products

Exposure Measurement

Serum levels (maternal, child and adult), umbilical cord serum, urine, and self-reported measures

Age range for exposure

all ages

Comparison

Individuals not exposed to e-waste

Outcome

Mental, neurodevelopmental, and physical health outcomes (including mechanistic events such as genotoxicity), education, aggression (crime and violence)

Outcome Measurement

Mental and physical health outcomes diagnosed by a health professional (using criteria and diagnosis) or direct physical measurements and blood tests, standardized tests for educational outcomes, but standardised/non-standardised screening instruments or self-reported health outcomes also accepted.

Study designs of interest

Prospective and retrospective cohort, cross-sectional and case-control studies included.
Limits on year of publication or language: December 2012 to January 2020.
Articles in LOTE deemed relevant based on its abstract are translated.

Exclusion criteria

Articles initially excluded if they are duplicates or if the title clearly demonstrates that the exposure and outcome of interest are not the focus of the article. Articles are then excluded based on the following:

- The article does not explore an association between WEEE/e-waste exposure and health, learning, and violence and criminal behaviour outcomes
- The article does not focus on physical health, mental health and neurodevelopment, learning problems, or violence and criminal behaviour related outcomes (ex. focus is on exposure levels or risk)
- Health outcomes for which no standardized diagnostic criteria are available (ex. Poor or ill health as an outcome)
- The study is a review article, abstract or letter to the editor
- The study does not look at human populations (animal, plant or cell)

Study inclusion/exclusion is completed independently. Results are reviewed and any disagreement is recorded. Results are discussed to reach consensus.

Data extraction sheet

The data extraction sheet is first pilot tested on 10 studies and then revised accordingly to include:

Identification of study

1. Record the first authors' last name, initials
2. Record the journal name
3. Record the year of publication
4. Record the volume number
5. Record the page numbers

Characteristics of study

1. Study period
2. Study design
3. Sample size
4. Study location
5. Chemicals included and analysed
6. Assessment of exposure
7. Health outcomes reported
8. Limitations and adjustments

Table S1. Risk of Bias Assessment

	Sample representative of population	Study design	Ascertainment of exposure to e-waste	Health outcomes	Masking	Adjustment of confounder	Selection of non-exposed controls	Risk of bias
Huo et al[1]	No<br>Score=1	Cross-sectional<br>Score= 1	Biological sample (urine)<br>Score= 2	Birth outcomes<br>Score= 2	No<br>Score= 1	Yes<br>Score= 2	Yes<br>Score= 2	Moderate
Li et al[2]	No<br>Score=1	Cross-sectional<br>Score=1	Biological sample (umbilical cord tissue)<br>Score= 2	Birth and genetic outcomes<br>Score= 2	No<br>Score= 1	Yes<br>Score=2	Yes<br>Score=2	Moderate
Xu et al[3]	No<br>Score=1	Cross-sectional<br>Score=1	Biological sample (placental specimen)<br>Score=2	Birth outcomes and placental proteome alteration<br>Score= 2	No<br>Score=1	Yes<br>Score=2	Yes<br>Score=2	Moderate
Xu et al[4]	No<br>Score=1	Cross-sectional<br>Score=1	Biological sample (placental specimen)<br>Score=2	Birth outcomes<br>Score= 2	No<br>Score=1	Yes<br>Score=2	Yes<br>Score=2	Moderate
Zhang et al[5]	No<br>Score=1	Cross sectional<br>Score=1	Biological sample (urine)<br>Score= 2	Birth outcomes<br>Score= 2	No<br>Score=1	Yes<br>Score=2	Yes<br>Score=2	Moderate
Xu et al[6]	No<br>Score=1	Cross-sectional<br>Score=1	Biological sample (blood)<br>Score= 2	Birth outcomes<br>Score= 2	No<br>Score=1	Yes<br>Score=2	Yes<br>Score=2	Moderate
Yang et al[7]	No<br>Score=1	Cross-sectional<br>Score=1	Biological sample (blood)<br>Score= 2	Birth outcomes and bone metabolic biomarkers<br>Score= 2	No<br>Score=1	Yes<br>Score=2	No<br>Score=1	Moderate
Zeng et al[8]	No<br>Score=1	Cross sectional<br>Score=1	Biological sample (blood)<br>Score= 2	Birth outcomes<br>Score= 2	No<br>Score=1	Yes<br>Score=2	Yes<br>Score=2	Moderate
Cai et al[9]	No<br>Score=1	Cross-sectional<br>Score=1	Biological sample (blood)<br>Score= 2	Sensory integration using standardized tools<br>Score=2	No<br>Score=1	Yes<br>Score=2	Yes<br>Score=2	Moderate
Liu et al[10]	No<br>Score=1	Cross-sectional<br>Score=1	Biological sample (blood)<br>Score= 2	Cognitive and language scores using standardized tools<br>Score=2	No<br>Score=1	Yes<br>Score=2	Yes<br>Score=2	Moderate
Liu et al[11]	No<br>Score=1	Cross-sectional<br>Score=1	Biological sample (blood)<br>Score= 2	Mental outcome (ADHD) using standardized tools<br>Score=2	No<br>Score=1	Yes<br>Score=2	No<br>Score=1	Moderate
Zhang et al[12]	No<br>Score=1	Cross-sectional<br>Score=1	Biological sample (blood)<br>Score= 2	Mental outcome (ADHD) using standardized tools<br>Score=2	No<br>Score=1	Yes<br>Score=2	No<br>Score=1	Moderate

Liu et al[13]	No<br>Score=1	Cross-sectional<br>Score=1	Biological sample (blood)<br>Score= 2	Cognitive and language scores using standardized tools<br>Score=2	No<br>Score=1	Yes<br>Score=2	Yes<br>Score=2	Moderate
Lv et al[14]	No<br>Score=1	Cross-sectional<br>Score=1	Biological sample (blood)<br>Score= 2	Hormonal outcomes<br>Score=2	No<br>Score=1	Yes<br>Score=2	Yes<br>Score=2	Moderate
Ben et al[15]	No<br>Score=1	Cross-sectional<br>Score=1	Biological sample (blood)<br>Score= 2	Hormonal outcomes<br>Score=2	No<br>Score=1	No<br>Score=1	Yes<br>Score=2	High
Zheng et al[16]	No<br>Score=1	Cross-sectional<br>Score=1	Biological sample (blood, umbilical cord blood and placenta)<br>Score= 2	Hormonal outcomes<br>Score=2	No<br>Score=1	No<br>Score=1	Yes<br>Score=2	High
Xu et al[17]	No<br>Score=1	Cross-sectional<br>Score=1	Biological sample (umbilical cord blood, placental tissue)<br>Score= 2	Hormonal and gene expression<br>Score=2	No<br>Score=1	No<br>Score=1	Yes<br>Score=2	Moderate
Xu et al[18]	No<br>Score=1	Cross-sectional<br>Score=1	Biological sample (blood)<br>Score= 2	Hormonal outcomes<br>Score=2	No<br>Score=1	Yes<br>Score=2	No<br>Score=1	High
Xu et al[19]	No<br>Score=1	Cross-sectional<br>Score=1	Biological sample (blood)<br>Score= 2	Hormonal outcomes<br>Score=2	No<br>Score=1	No<br>Score=1	Yes<br>Score=2	Moderate
Eguchi et al[20]	No<br>Score=1	Cross-sectional<br>Score=1	Biological sample (blood)<br>Score= 2	Hormonal outcomes<br>Score=2	No<br>Score=1	Yes<br>Score=2	Yes<br>Score=2	Moderate
Eguchi et al[21]	No<br>Score=1	Cross-sectional<br>Score=1	Biological sample (blood)<br>Score= 2	Hormonal outcomes<br>Score=2	No<br>Score=1	Yes<br>Score=2	Yes<br>Score=2	Moderate
Xu et al[22]	No<br>Score=1	Cross-sectional<br>Score=1	Biological sample (blood)<br>Score= 2	Hormonal outcomes<br>Score=2	No<br>Score=1	Yes<br>Score=2	Yes<br>Score=2	Moderate
Guo et al[23]	No<br>Score=1	Cross-sectional<br>Score=1	Biological sample (blood)<br>Score= 2	Hormonal and gene expression<br>Score=2	No<br>Score=1	Yes<br>Score=2	Yes<br>Score=2	Moderate
Zheng et al[24]	No<br>Score=1	Cross-sectional<br>Score=1	Biological sample (blood)<br>Score= 2	Hormonal and gene expression<br>Score=2	No<br>Score=1	Yes<br>Score=2	No<br>Score=1	High
Yan et al[25]	No<br>Score=1	Cross-sectional<br>Score=1	Biological sample (blood)<br>Score= 2	Hormonal outcomes<br>Score=2	No<br>Score=1	Yes<br>Score=2	No<br>Score=1	High
Guo et al[26]	No<br>Score=1	Cross-sectional<br>Score=1	Biological sample (blood)<br>Score= 2	Hormonal outcomes<br>Score=2	No<br>Score=1	Yes<br>Score=2	Yes<br>Score=2	Moderate
Zhou et al[27]	No<br>Score=1	Cross-sectional<br>Score=1	Self-reported<br>Score= 1	Hormonal outcomes<br>Score=2	No<br>Score=1	No<br>Score=1	Yes<br>Score=2	Moderate
Cao et al[28]	No<br>Score=1	Cross-sectional<br>Score=1	Biological sample (blood)<br>Score= 2	Immunological outcomes<br>Score=2	No<br>Score=1	Yes<br>Score=2	Yes<br>Score=2	Moderate
Huo et al[29]	No<br>Score=1	Cross-sectional<br>Score=1	Biological sample (blood)<br>Score= 2	Immunological outcomes<br>Score=2	No<br>Score=1	Yes<br>Score=2	Yes<br>Score=2	Moderate
Zhang et al[30]	No<br>Score=1	Cross-sectional<br>Score=1	Biological sample (blood)<br>Score= 2	Immunological outcomes<br>Score=2	No<br>Score=1	Yes<br>Score=2	Yes<br>Score=2	Moderate

Li et al[2]	No Score=1	Cross-sectional Score=1	Biological sample (umbilical cord tissue) Score= 2	Birth and genetic outcomes Score= 2	No Score= 1	Yes Score=2	Yes Score=2	Moderate
Lin et al[31]	No Score=1	Cross-sectional Score=1	Biological sample (placental tissue) Score= 2	Genetic outcomes Score= 2	No Score= 1	No Score=1	Yes Score=2	High
Zeng et al[32]	No Score=1	Cross-sectional Score=1	Biological sample (umbilical cord blood) Score= 2	Genetic outcomes Score= 2	No Score= 1	Yes Score=2	Yes Score=2	Moderate
Huo et al[33]	No Score=1	Cross-sectional Score=1	Biological sample (blood) Score= 2	Genetic outcomes Score= 2	No Score= 1	No Score=1	Yes Score=2	High
Xu et al[34]	No Score=1	Cross-sectional Score=1	Biological sample (blood) Score= 2	Genetic and olfactory outcomes Score= 2	No Score= 1	Yes Score=2	Yes Score=2	Moderate
Li et al[35]	No Score=1	Cross-sectional Score=1	Biological sample (blood) Score= 2	Genetic outcomes Score= 2	No Score= 1	No Score=1	Yes Score=2	High
Yuan et al[36]	No Score=1	Cohort Score=2	Biological sample (blood) Score= 2	Genetic outcomes Score= 2	No Score= 1	No Score=1	No Score=1	High
Li et al[37]	No Score=1	Cross-sectional Score=1	Biological sample (blood) Score= 2	Genetic outcomes Score= 2	No Score= 1	No Score=1	Yes Score=2	Moderate
He et al[38]	No Score=1	Cross-sectional Score=1	Biological sample (blood) Score= 2	Genetic outcomes Score= 2	No Score= 1	No Score=1	Yes Score=2	Moderate
Guo et al[23]	No Score=1	Cross-sectional Score=1	Biological sample (blood) Score= 2	Hormonal and oxidative outcomes Score=2	No Score=1	Yes Score=2	Yes Score=2	Moderate
Ni et al[39]	No Score=1	Cross-sectional Score=1	Biological sample (umbilical cord blood) Score= 2	Oxidative damage Score=2	No Score=1	Yes Score=2	Yes Score=2	Moderate
Zhou et al[27]	No Score=1	Cross-sectional Score=1	Self-reported Score= 1	Hormonal and oxidative damage Score=2	No Score=1	No Score=1	Yes Score=2	High
Xu et al[40]	No Score=1	Cross-sectional Score=1	Biological sample (blood and urine) Score= 2	Oxidative damage Score=2	No Score=1	No Score=1	No Score=1	High
Li et al[41]	No Score=1	Cross-sectional Score=1	Biological sample (blood) Score= 2	Oxidative damage Score=2	No Score=1	No Score=1	Yes Score=2	Moderate
Lu et al[42]	No Score=1	Cross-sectional Score=1	Biological sample (urine) Score= 2	Oxidative damage Score=2	No Score=1	Yes Score=2	Yes Score=2	Moderate
Lu et al[43]	No Score=1	Cross-sectional Score=1	Biological sample (urine) Score= 2	Oxidative damage Score=2	No Score=1	No Score=1	Yes Score=2	Moderate
Yang et al[44]	No Score=1	Cross-sectional Score=1	Biological sample (urine) Score= 2	Oxidative damage Score=2	No Score=1	Yes Score=2	No Score=1	High
Zhang et al [45]	No	Cross-sectional	Biological sample (urine)	Oxidative damage	No	No	Yes	Moderate

	Score=1	Score=1	Score= 2	Score=2	Score=1	Score=1	Score=2	
Zhang et al[46]	No<br>Score=1	Cross-sectional<br>Score=1	Biological sample (urine)<br>Score= 2	Oxidative damage<br>Score=2	No<br>Score=1	No<br>Score=1	Yes<br>Score=2	High
Zhang et al[47]	No<br>Score=1	Cross-sectional<br>Score=1	Biological sample (urine)<br>Score= 2	Oxidative damage<br>Score=2	No<br>Score=1	No<br>Score=1	Yes<br>Score=2	High
Zeng et al[48]	No<br>Score=1	Cross-sectional<br>Score=1	Biological sample (blood)<br>Score= 2	Respiratory outcomes<br>Score=2	No<br>Score=1	Yes<br>Score=2	Yes<br>Score=2	Moderate
Zeng et al[49]	No<br>Score=1	Cross-sectional<br>Score=1	Anthropometric measure<br>Score= 2	Respiratory outcomes<br>Score=2	No<br>Score=1	Yes<br>Score=2	Yes<br>Score=2	Moderate
Zeng et al[50]	No<br>Score=1	Cross-sectional<br>Score=1	Biological and environmental sample<br>Sore=2	Respiratory outcomes<br>Score=2	No<br>Score=1	Yes<br>Score=2	Yes<br>Score=2	Moderate
Zhang et al[51]	No<br>Score=1	Cross-sectional<br>Score=1	Ambient particulate matter<br>Score=1	Respiratory and pro-inflammatory cytokines<br>Score=2	No<br>Score=1	Yes<br>Score=2	Yes<br>Score=2	Moderate
Lu et al[52]	No<br>Score=1	Cross-sectional<br>Score=1	Biological sample (blood)<br>Score= 2	Cardiovascular outcomes<br>Score=2	No<br>Score=1	Yes<br>Score=2	Yes<br>Score=2	Moderate
Zheng et al[53]	No<br>Score=1	Cross-sectional<br>Score=1	Biological sample (blood and urine)<br>Score= 2	Cardiovascular outcomes<br>Score=2	No<br>Score=1	No<br>Score=1	Yes<br>Score=2	High
Cong et al[54]	No<br>Score=1	Cross-sectional<br>Score=1	Air pollutants<br>Score=1	Cardiovascular outcomes<br>Score=2	No<br>Score=1	Yes<br>Score=2	Yes<br>Score=2	Moderate
Gangwar et al[55]	No<br>Score=1	Cross-sectional<br>Score=1	Biological sample and ambient particulate matter<br>Score=2	Cardiovascular outcomes<br>Score=2	No<br>Score=1	No<br>Score=1	Yes<br>Score=2	High
Burns et al[56]	No<br>Score=1	Cross-sectional<br>Score=1	Self-reported measure<br>Score=1	Noise and cardiovascular outcomes<br>Score=1	No<br>Score=1	Yes<br>Score=2	No<br>Score=1	High
Dai et al[57]	No<br>Score=1	Cross-sectional<br>Score=1	Biological sample (blood)<br>Score= 2	Hematopoietic function<br>Score=2	No<br>Score=1	Yes<br>Score=2	Yes<br>Score=2	Moderate
Zeng et al[58]	No<br>Score=1	Cross-sectional<br>Score=1	Biological sample (blood)<br>Score= 2	Coagulation impairment<br>Score=2	No<br>Score=1	Yes<br>Score=2	Yes<br>Score=2	Moderate
Zhang et al[59]	No<br>Score=1	Cross-sectional<br>Score=1	Biological sample (blood)<br>Score= 2	Natural and innate immunity<br>Score=2	No<br>Score=1	Yes<br>Score=2	Yes<br>Score=2	Moderate
Dai et al[60]	No<br>Score=1	Cross-sectional<br>Score=1	Biological sample (blood)<br>Score= 2	Haematological parameters<br>Score=2	No<br>Score=1	Yes<br>Score=2	Yes<br>Score=2	Moderate
Xu et al[22]	No<br>Score=1	Cross-sectional<br>Score=1	Biological sample (blood)<br>Score= 2	Haematological parameters<br>Score=2	No<br>Score=1	Yes<br>Score=2	Yes<br>Score=2	Moderate

Chen et al[61]	No<br>Score=1	Cross-sectional<br>Score=1	Biological sample (blood)<br>Score= 2	Haematological parameters<br>Score=2	No<br>Score=1	Yes<br>Score=2	Yes<br>Score=2	Moderate
Lin et al[62]	No<br>Score=1	Cross-sectional<br>Score=1	Biological sample (blood)<br>Score= 2	Antibody titers<br>Score=2	No<br>Score=1	Yes<br>Score=2	Yes<br>Score=2	Moderate
Lin et al[63]	No<br>Score=1	Cross-sectional<br>Score=1	Biological sample (blood)<br>Score= 2	Antibody titers<br>Score=2	No<br>Score=1	No<br>Score=1	Yes<br>Score=2	High
Xu et al[64]	No<br>Score=1	Cross-sectional<br>Score=1	Biological sample (blood)<br>Score= 2	Antibody titers<br>Score=2	No<br>Score=1	Yes<br>Score=2	Yes<br>Score=2	Moderate
Liu et al[65]	No<br>Score=1	Cross-sectional<br>Score=1	Biological sample (blood)<br>Score= 2	Pure tone air conduction (PTA)<br>Score=2	No<br>Score=1	Yes<br>Score=2	Yes<br>Score=2	Moderate
Xu et al[34]	No<br>Score=1	Cross-sectional<br>Score=1	Biological sample (blood)<br>Score= 2	DNA methylation<br>Hearing ability<br>Score=2	No<br>Score=1	Yes<br>Score=2	Yes<br>Score=2	Moderate
Zhang et al[66]	No<br>Score=1	Cross-sectional<br>Score=1	Biological sample (blood)<br>Score= 2	Olfactory memory<br>Score=2	No<br>Score=1	No<br>Score=1	Yes<br>Score=2	High
Yu et al[67]	No<br>Score=1	Cross-sectional<br>Score=1	Biological sample (blood)<br>Score= 2	Male reproductive health<br>Score=2	No<br>Score=1	Yes<br>Score=2	Yes<br>Score=2	Moderate
Wang et al[68]	No<br>Score=1	Cross-sectional<br>Score=1	Biological sample (blood)<br>Score= 2	Male reproductive health<br>Score=2	No<br>Score=1	Yes<br>Score=2	Yes<br>Score=2	Moderate
Chen et al[61]	No<br>Score=1	Cross-sectional<br>Score=1	Biological sample (blood)<br>Score= 2	Hepatic enzymes<br>Score=2	No<br>Score=1	Yes<br>Score=2	Yes<br>Score=2	Moderate
Xu et al[22]	No<br>Score=1	Cross-sectional<br>Score=1	Biological sample (blood)<br>Score= 2	Renal function Score=2	No<br>Score=1	Yes<br>Score=2	Yes<br>Score=2	Moderate
Hou et al[69]	No<br>Score=1	Cross-sectional<br>Score=1	Biological sample (blood)<br>Score= 2	Oral health (Salivary sialic acid and dental caries)<br>Score=2	No<br>Score=1	Yes<br>Score=2	Yes<br>Score=2	Moderate
Song et al[70]	No<br>Score=1	Cross-sectional<br>Score=1	Biological sample (blood)<br>Score= 2	Metabolic disease<br>Score=2	No<br>Score=1	No<br>Score=1	Yes<br>Score=2	High

Table S2. List of excluded studies at full-text screening stage with brief reasons

sl	Author	Title	Year	Article Type	Inclusion/Exclusion	Reason for exclusion
1	Abdallah MA[71]	Environmental Occurrence, Analysis and Human Exposure to the Flame Retardant tetrabromobisphenol-A (TBBP-A)-A Review	2016	Review	Excluded	Review article
2	Azad M, Ismail M, Hossin MI[72]	Generation of electronic-waste and its impact on environment and public health in Malaysia	2017	Review	Excluded	Review article
3	Ackah M[73]	Informal E-waste recycling in developing countries: review of metal(loid)s pollution, environmental impacts and transport pathways	2017	Review	Excluded	Review article
4	Ackah M[74]	Soil elemental concentrations, geoaccumulation index, non-carcinogenic and carcinogenic risks in functional areas of an informal e-waste recycling area in Accra, Ghana	2019	Article	Excluded	No human population investigated
5	Akortia E, Olukunle OI, Daso AP, Okonkwo JO[75]	Soil concentrations of polybrominated diphenyl ethers and trace metals from an electronic waste dump site in the Greater Accra Region, Ghana: Implications for human exposure	2017	Article	Excluded	No human population investigated
6	Amankwaa EF, AdovorTsikudo KA, Bowman J[76]	'Away' is a place: The impact of electronic waste recycling on blood lead levels in Ghana	2017	Article	Excluded	No health outcomes reported
7	An et al[77]	Pollution profiles and health risk assessment of VOCs emitted during e-waste dismantling processes associated with different dismantling methods	2014	Article	Excluded	No human population investigated
8	Anderson JC, Cropp A, Paradise JC[78]	Solubility of indium-tin oxide in simulated lung and gastric fluids: Pathways for human intake	2017	Article	Excluded	In vitro experiment
9	Anh et al[79]	Polybrominated diphenyl ethers in plastic products, indoor dust, sediment and fish from informal e-waste recycling sites in Vietnam: a comprehensive assessment of contamination, accumulation pattern, emissions, and human exposure	2017	Article	Excluded	No health outcomes reported
10	Annamalai J[80]	Occupational health hazards related to informal recycling of E-waste in India: An overview	2015	Article	Excluded	Review article
11	Arain AL, Neitzel RL[81]	A review of biomarkers used for assessing human exposure to metals from E-waste	2019	Review	Excluded	Review article
12	Asamoah et al[82]	Assessment of PCBs and exposure risk to infants in breast milk of primiparae and multiparae mothers in an electronic waste hot spot and non-hot spot areas in Ghana	2018	Article	Excluded	No health outcomes reported
13	Asamoah et al[83]	PAHs contamination levels in the breast milk of Ghanaian women from an e-waste recycling site and a residential area	2019	Article	Excluded	No health outcomes reported
14	Awasthi et al[84]	Environmental pollution and human body burden from improper recycling of e-waste in China: A short-review	2018	Review	Excluded	Review article
15	Awasthi et al[85]	Relationship between e-waste recycling and human health risk in India: a critical review	2016	Review	Excluded	Review article
16	Awasthi et al[86]	Environmental pollution of electronic waste recycling in India: A critical review	2016	Review	Excluded	Review article
17	Bai et al[87]	A pilot study of metabolites of organophosphorus flame retardants in paired maternal urine and amniotic fluid samples: Potential exposure risks of tributyl phosphate to pregnant women	2019	Article	Excluded	No health outcomes reported

18	Basu et al[88]	Occupational and Environmental Health Risks Associated with Informal Sector Activities-Selected Case Studies from West Africa	2016	Review	Excluded	Review article
19	Becker et al[89]	Environmental impact of bottles, teats, and packaging in maternity units	2019	Letter	Excluded	Letter to editor
20	Ben et al[90]	Dechlorane Plus and its dechlorinated analogs from an e-waste recycling center in maternal serum and breast milk of women in Wenling, China	2013	Article	Excluded	No health outcomes reported
21	Borthakur A[91]	Health and Environmental Hazards of Electronic Waste in India	2016	Special report	Excluded	Report
22	Bruce-Vanderpuije et al[92]	Background levels of dioxin-like polychlorinated biphenyls (dlPCBs), polychlorinated, polybrominated and mixed halogenated dibenzo-p-dioxins and dibenzofurans (PCDD/Fs, PBDD/Fs & PXDD/Fs) in sera of pregnant women in Accra, Ghana	2019	Article	Excluded	No health outcomes reported
23	Budnik LT, Casteleyn L[93]	Mercury pollution in modern times and its socio-medical consequences	2019	Review	Excluded	Review article
24	Burns et al[94]	Stress, health, noise exposures, and injuries among electronic waste recycling workers in Ghana	2019	Article	Excluded	No health outcomes reported
25	Cabrera-Rodríguez et al[95]	Occurrence of 44 elements in human cord blood and their association with growth indicators in newborns	2018	Article	Excluded	No exposure to e-waste
26	Cai et al[96]	The history, status, gaps, and future directions of neurotoxicology in China	2016	Review	Excluded	Review article
27	Cao et el[97]	Bioaccessibility and human health risk assessment of metal(loid)s in soil from an e-waste open burning site in Agbogbloshie, Accra, Ghana	2020	Article	Excluded	No human population investigated
28	Caudle WM[98]	Occupational Metal Exposure and Parkinsonism	2017	Chapter	Excluded	Chapter
29	Ceballos et al[99]	Metal Exposures at three U.S. electronic scrap recycling facilities	2017	Article	Excluded	No health outcomes reported
30	Ceballos DM, Dong Z[100]	The formal electronic recycling industry: Challenges and opportunities in occupational and environmental health research	2016	Review	Excluded	Review article
31	Cesaro et al[101]	A device-specific prioritization strategy based on the potential for harm to human health in informal WEEE recycling	2018	Article	Excluded	No human population investigated
32	Chakraborty P[102]	Modeling the emission sources for polychlorinated biphenyls in India: Implications for human health risk assessment	2014	Article	Excluded	No human population investigated
33	Chakraborty et al[103]	PCBs and PCDD/Fs in soil from informal e-waste recycling sites and open dumpsites in India: Levels, congener profiles and health risk assessment	2018	Article	Excluded	No health outcomes reported
34	Ceballos et al[104]	A Pilot Assessment of Occupational Health Hazards in the US Electronic Scrap Recycling Industry	2015	Article	Excluded	No health outcomes reported
35	Chan et al[105]	Dietary intake of PBDEs of residents at two major electronic waste recycling sites in China	2013	Article	Excluded	No health outcomes reported

36	Chan et al[106]	Dietary exposure to polychlorinated dibenzo-p-dioxins and dibenzofurans via fish consumption and dioxin-like activity in fish determined by H4IIE-luc bioassay	2013	Article	Excluded	No human population investigated
37	Chan et al[107]	A review of environmental fate, body burdens, and human health risk assessment of PCDD/Fs at two typical electronic waste recycling sites in China	2013	Review	Excluded	Review article
38	Chen et al[108]	Combined Effects of Dust and Dietary Exposure of Occupational Workers and Local Residents to Short- and Medium-Chain Chlorinated Paraffins in a Mega E-Waste Recycling Industrial Park in South China	2018	Article	Excluded	No human population investigated
39	Chen et al[109]	Seasonal profiles of atmospheric PAHs in an e-waste dismantling area and their associated health risk considering bioaccessible PAHs in the human lung	2019	Article	Excluded	No human population investigated
40	Chen et al[110]	VOCs elimination and health risk reduction in e-waste dismantling workshop using integrated techniques of electrostatic precipitation with advanced oxidation technologies	2016	Article	Excluded	No human population investigated
41	Chen et al[111]	Dechlorane Plus in paired hair and serum samples from e-waste workers: Correlation and differences	2015	Article	Excluded	No health outcomes reported
42	Chen et al[112]	Children's non-carcinogenic health risk assessment of heavy metals exposure to residential indoor dust around an e-waste dismantling area in South China	2019	Chinese article	Excluded	Chinese article
43	Cole et al[113]	An assessment of achievements of the WEEE Directive in promoting movement up the waste hierarchy: experiences in the UK	2019	Article	Excluded	No health outcomes reported
44	Dartey et al[114]	Essential and non-essential trace elements among working populations in Ghana	2017	Article	Excluded	No health outcomes reported
45	Davis JM, Garb Y[115]	A strong spatial association between e-waste burn sites and childhood lymphoma in the West Bank, Palestine	2019	Article	Excluded	No human population investigated
46	Decharat et al[116]	Urinary mercury levels among workers in e-waste shops in Nakhon Si Thammarat Province, Thailand	2018	Article	Excluded	No health outcomes reported
47	Déportes et al[117]	Potential health impacts of waste electrical and electronic equipment management: A brief comparison between emerging and developed countries	2018	Article	Excluded	No health outcomes reported
48	Devi et al[118]	Polychlorinated Biphenyls in Surface Soil from North-East India: Implication for Sources Apportionment and Health-Risk Assessment	2018	Article	Excluded	No health outcomes reported
49	Die et al[119]	Concentrations and occupational exposure assessment of polybrominated diphenyl ethers in modern Chinese e-waste dismantling workshops	2019	Article	Excluded	No human population investigated
50	Dowling et al[120]	Reducing blood lead levels in children exposed to electronic waste recycling in Montevideo	2016	Abstract	Excluded	Abstract

51	Du et al[121]	Cytogenetics alteration in adult men involved in the recycling of electronic wastes	2018	Tombstone	Excluded	Data article
52	Esogwah et al[122]	Hepatotoxic effects of electronic waste leachate on rats	2014	Abstract	Excluded	Abstract
53	Etzel, RA[123]	Environmental hazards that matter for children's health	2015	Review	Excluded	Review article
54	Fang et al[124]	PM10 and PM2.5 and health risk assessment for heavy metals in a typical factory for cathode ray tube television recycling	2013	Article	Excluded	No human population investigated
55	Feldt et al[125]	High levels of PAH-metabolites in urine of e-waste recycling workers from Agbogbloshie, Ghana	2014	Article	Excluded	No health outcomes reported
56	Aguilera et al[126]	Assessment of exposure to persistent organic pollutants and alteration on thyroid hormone levels in Mexican children from a community dedicated to electronic waste recycling	2016	Article	Excluded	Abstract
57	Fu et al[127]	Influence of E-waste dismantling and its regulations: Temporal trend, spatial distribution of heavy metals in rice grains, and its potential health risk	2013	Article	Excluded	No human population investigated
58	Giudice, LC[128]	Environmental toxicants: hidden players on the reproductive stage	2016	Preface	Excluded	Preface
59	Gomathi et al[129]	Study of E-waste- hazards & recycling techniques- A review	2015	Review	Excluded	Review article
60	González-Antuña et al[130]	Simultaneous quantification of 49 elements associated to e-waste in human blood by ICP-MS for routine analysis	2017	Article	Excluded	Protocol article
61	Grant et al[131]	Health consequences of exposure to e-waste: A systematic review	2013	Review	Excluded	Review article
62	Gravel et al[132]	Assessment of Occupational Exposure to Organic Flame Retardants: A Systematic Review	2019	Review	Excluded	Review article
63	Gwenzi et al[133]	Electronic waste recycling exposure and hormone levels in workers	2019	Abstract	Excluded	Abstract
64	Gravel et al[134]	Electronic waste recycling in quÉbec, canada: Hiring practices and occupational health and safety	2019	Abstract	Excluded	Abstract
65	Guo et al[135]	Blood lead levels and associated factors among children in Guiyu of China: A population-based study	2014	Article	Excluded	No health outcomes reported
66	Gwenzi et al[136]	Sources, behaviour, and environmental and human health risks of high-technology rare earth elements as emerging contaminants	2018	Review	Excluded	Review article
67	Ha et al[137]	Pleiotropic roles of Ca+2/calmodulin-dependent pathways in regulating cadmium-induced toxicity in human osteoblast-like cell lines	2016	Article	Excluded	In vitro experiment
68	Hahladakis et al[138]	Assessment of released heavy metals from electrical and electronic equipment (EEE) existing in shipwrecks through laboratory-scale simulation reactor	2013	Article	Excluded	No human population investigated
69	Han et al[139]	Ecological and health risks assessment and spatial distribution of residual heavy metals in the soil of an e-waste circular economy park in Tianjin, China	2018	Article	Excluded	No human population investigated
70	He et al[140]	Occurrence of organophosphorus flame retardants in indoor dust in multiple microenvironments of southern China and implications for human exposure	2015	Article	Excluded	No human population investigated

71	He et al[141]	Organic contaminants and heavy metals in indoor dust from e-waste recycling, rural, and urban areas in South China: Spatial characteristics and implications for human exposure	2017	Article	Excluded	No human population investigated
72	Heacock et al[142]	E-waste and harm to vulnerable populations: A growing global problem	2016	Commentary	Excluded	Commentary
73	Heacock et al[143]	E-waste: the growing global problem and next steps	2016	Review	Excluded	Review
74	Hennig, B[144]	Protective influence of healthful nutrition on mechanisms of environmental pollutant toxicity and disease risks.	2017	Article	Excluded	No human population investigated
75	Hennig et al[145]	The role of nutrition in influencing mechanisms involved in environmentally mediated diseases	2018	Review	Excluded	Review article
76	Henríquez-Hernández et al[146]	Blood levels of toxic metals and rare earth elements commonly found in e-waste may exert subtle effects on hemoglobin concentration in sub-Saharan immigrants	2017	Article	Excluded	No exposure to e-waste
77	Henríquez-Hernández et al[147]	Study of the influencing factors of the blood levels of toxic elements in Africans from 16 countries	2017	Article	Excluded	No exposure to e-waste
78	Henríquez-Hernández et al[148]	Biomonitoring of 45 inorganic elements measured in plasma from Spanish subjects: A cross-sectional study in Andalusian population	2020	Article	Excluded	No exposure to e-waste
79	Hou et al[149]	Corrigendum to “Elevated levels of lead exposure and impact on the anti-inflammatory ability of oral sialic acids among preschool children in e-waste areas”	2020	Erratum	Excluded	Erratum
80	Hu et al[150]	Spatial distribution of polychlorinated dibenzo-p-dioxins and dibenzo-furans (PCDDs/Fs) in dust, soil, sediment and health risk assessment from an intensive electronic waste recycling site in Southern China	2013	Article	Excluded	No human population investigated
81	Huang et al[151]	Potential health risk for residents around a typical e-waste recycling zone via inhalation of size-fractionated particle-bound heavy metals	2016	Article	Excluded	No human population investigated
82	Huang et al[152]	E-waste disposal effects on the aquatic environment: Accra, Ghana	2014	Review	Excluded	Review article
83	Huang et al[153]	Levels and risk factors of antimony contamination in human hair from an electronic waste recycling area, Guiyu, China	2015	Article	Excluded	No health outcomes reported
84	Huo et al[154]	Impact of informal e-waste recycling on human health	2019	Review chinese	Excluded	Review article
85	Hussain, M Mumtaz, S[155]	E-waste: Impacts, issues and management strategies	2014	Article	Excluded	No health outcomes reported
86	Ibe et al[156]	Environmental and health implications of trace metal concentrations in street dusts around some electronic repair workshops in Owerri, Southeastern Nigeria	2018	Article	Excluded	No human population investigated
87	Ilankoon et al[157]	E-waste in the international context – A review of trade flows, regulations, hazards, waste management strategies and technologies for value recovery	2018	Review	Excluded	Review article
88	Iqbal et al[158]	Emerging issue of e-waste in Pakistan: A review of status, research needs and data gaps	2015	Review	Excluded	Review article

89	Iqbal et al[159]	E-Waste Driven Pollution in Pakistan: The First Evidence of Environmental and Human Exposure to Flame Retardants (FRs) in Karachi City	2017	Article	Excluded	No human population investigated
90	Isara et al[160]	Serum lipid profile and atherogenic indices of e-waste workers in benin city, nigeria	2018	Abstract	Excluded	Abstract
91	Jafarzadeh-Ghoushchi, S.Dorosti, S[161]	Effects of exposure to a variety of waste on human health - A review	2017	Review	Excluded	Review article
92	Jiang et al[162]	Daily intake of polybrominated diphenyl ethers via dust and diet from an e-waste recycling area in China	2014	Article	Excluded	No health outcomes reported
93	Jiang et al[163]	Polybrominated diphenyl ethers in the environment and human external and internal exposure in China: A review	2019	Review	Excluded	Review article
94	Jibiri et al[164]	Assessment of radiation exposure levels at Alaba e-waste dumpsite in comparison with municipal waste dumpsites in southwest Nigeria	2014	Article	Excluded	No health outcomes reported
95	Julander et al[165]	Formal recycling of e-waste leads to increased exposure to toxic metals: an occupational exposure study from Sweden	2014	Article	Excluded	No health outcomes reported
96	Kang et al[166]	Potential environmental and human health impacts of rechargeable lithium batteries in electronic waste	2013	Article	Excluded	No health outcomes reported
97	Khan et al[167]	New insight into the distribution pattern, levels, and risk diagnosis of FRs in indoor and outdoor air at low- and high-altitude zones of Pakistan: Implications for sources and exposure	2017	Article	Excluded	No human population investigated
98	Khlaif, N Qumsiyeh, MB[168]	Genotoxicity of recycled electronic waste in Idhna, Hebron District, occupied Palestinian territory: A case-controlled study	2018	Abstract	Excluded	Abstract
99	Kim et al[169]	Contamination by perfluorinated compounds in water near waste recycling and disposal sites in Vietnam	2013	Article	Excluded	No human population investigated
100	Kim et al[170]	Metal concentrations in pregnant women and neonates from informal electronic waste recycling	2018	Article	Excluded	No health outcomes reported
101	Klinčić et al[171]	Levels and distribution of polybrominated diphenyl ethers in humans and environmental compartments: a comprehensive review of the last five years of research	2020	Review	Excluded	Review article
102	Krishnamoorthy et al[172]	Emerging public health threat of e-waste management: global and Indian perspective	2018	Review	Excluded	Review article
103	Kuo et al[173]	Polybrominated diphenyl ethers (PBDEs) in plasma from E-waste recyclers, outdoor and indoor workers in the Puget Sound, WA region	2019	Article	Excluded	No health outcomes reported
104	Laborde, A[174]	Recycling of e-waste: An estimation of cumulative health risks posed to vulnerable populations through exposure to neurotoxicant mixtures	2016	Abstract	Excluded	Abstract
105	Laborde et al[175]	Children’s health in Latin America: The infuence of environmental exposures	2015	Review	Excluded	Review article

106	Labunska et al[176]	Human dietary intake of organohalogen contaminants at e-waste recycling sites in Eastern China	2015	Article	Excluded	No health outcomes reported
107	Labunska et al[177]	Domestic duck eggs: An important pathway of human exposure to PBDEs around E-waste and scrap metal processing areas in Eastern China	2013	Article	Excluded	No human population investigated
108	Labunska et al[178]	Human dietary exposure to PBDEs around E-waste recycling sites in Eastern China	2014	Article	Excluded	No human population investigated
109	Landrigan et al[179]	Health Consequences of Environmental Exposures: Changing Global Patterns of Exposure and Disease	2016	Review	Excluded	Review article
110	Laskaris et al[180]	Derivation of Time-Activity Data Using Wearable Cameras and Measures of Personal Inhalation Exposure among Workers at an Informal Electronic-Waste Recovery Site in Ghana	2019	Article	Excluded	No health outcomes reported
111	Lau et al[181]	Human health risk assessment based on trace metals in suspended air particulates, surface dust, and floor dust from e-waste recycling workshops in Hong Kong, China	2014	Article	Excluded	No human population investigated
112	Lecler et al[182]	Exposure to hazardous substances in Cathode Ray Tube (CRT) recycling sites in France	2015	Article	Excluded	No health outcomes reported
113	Leyssens et al[183]	Cobalt toxicity in humans—A review of the potential sources and systemic health effects	2017	Review	Excluded	Review article
114	Li et al[184]	Occurrence of multiple classes of emerging photoinitiators in indoor dust from E-waste recycling facilities and adjacent communities in South China and implications for human exposure	2020	Article	Excluded	No health outcomes reported
115	Li et al[185]	Occurrence, behavior and human health risk assessment of dechlorane plus and related compounds in indoor dust of China	2015	Article	Excluded	No human population investigated
116	Li et al[186]	Human exposure levels of PAEs in an e-waste recycling area: Get insight into impacts of spatial variation and manipulation mode	2019	Article	Excluded	No health outcomes reported
117	Li et al[187]	Accumulation of polybrominated diphenyl ethers in breast milk of women from an e-waste recycling center in China	2017	Article	Excluded	No health outcomes reported
118	Liang et al[188]	Brominated flame retardants in the hair and serum samples from an e-waste recycling area in southeastern China: the possibility of using hair for biomonitoring	2016	Article	Excluded	No health outcomes reported
119	Lin et al[189]	Insights into biomonitoring of human exposure to polycyclic aromatic hydrocarbons with hair analysis: A case study in e-waste recycling area	2020	Article	Excluded	No health outcomes reported
120	Liu et al[190]	The study of exposure levels of dioxin-like compounds in cord blood of newborns in an e-waste dismantling area in Guangdong Province	2019	Article chinese	Excluded	Chinese article
121	Liu et al[191]	Cutting down on the ozone and SOA formation as well as health risks of VOCs emitted from e-waste dismantlement by integration technique	2019	Article	Excluded	No health outcomes reported

122	Liu et al[192]	Comparing pollution patterns and human exposure to atmospheric PBDEs and PCBs emitted from different e-waste dismantling processes	2019	Article	Excluded	No human population investigated
123	Luo et al[193]	Size-dependent atmospheric deposition and inhalation exposure of particle-bound organophosphate flame retardants	2016	Article	Excluded	No human population investigated
124	Luo et al[194]	Size-dependent distribution and inhalation cancer risk of particle-bound polycyclic aromatic hydrocarbons at a typical e-waste recycling and an urban site	2015	Article	Excluded	No human population investigated
125	Luo et al[195]	Health risk characterization for resident inhalation exposure to particle-bound halogenated flame retardants in a typical e-waste recycling zone	2014	Article	Excluded	No human population investigated
126	Luzardo et al[196]	Socioeconomic development as a determinant of the levels of organochlorine pesticides and PCBs in the inhabitants of Western and Central African countries	2014	Article	Excluded	No health outcomes reported
127	Ma, L[197]	Effects of e-waste exposure on the synthesis of hemoglobin in preschool children	2014	Abstract	Excluded	Abstract
128	Ma et al[198]	Effects of E-waste exposure on the synthesis of haemoglobin in preschool children	2014	Abstract	Excluded	Abstract
129	Ma et al[199]	Polychlorinated biphenyls and their hydroxylated metabolites in the serum of e-waste dismantling workers from eastern China	2018	Article	Excluded	No health outcome reported
130	Malliari, E Kalantzi, OI[200]	Children's exposure to brominated flame retardants in indoor environments - A review	2017	Review	Excluded	Review article
131	Man et al[201]	Human health risk assessment of soil dioxin/furans contamination and dioxin-like activity determined by ethoxyresorufin-O-deethylase bioassay	2015	Article	Excluded	No human population investigated
132	Man et al[202]	A pilot study on health risk assessment based on body loadings of PCBs of lactating mothers at Taizhou, China, the world's major site for recycling transformers	2017	Article	Excluded	No health outcomes reported
133	Man et al[203]	Cancer risk assessments of Hong Kong soils contaminated by polycyclic aromatic hydrocarbons	2013	Article	Excluded	No health outcomes reported
134	Matovu et al[204]	Polybrominated diphenyl ethers in mothers' breast milk and associated health risk to nursing infants in Uganda	2019	Article	Excluded	No exposure to e-waste
135	Méndez et al[205]	Blood lead levels and potential sources of lead exposure among children in Montevideo, Uruguay	2016	Abstract	Excluded	Abstract
136	Mishra, S[206]	Perceived and manifested health problems among informal e-waste handlers: A scoping review	2019	Review/PMC	Excluded	Review article
137	Mishra et al[207]	Exploring the Awareness Regarding E-waste and its Health Hazards among the Informal Handlers in Musheerabad Area of Hyderabad	2017	Article/PMC	Excluded	No health outcomes reported

138	Mo et al[208]	Dechlorane Plus flame retardant in kingfishers (Alcedoatthis) from an electronic waste recycling site and a reference site, South China: Influence of residue levels on the isomeric composition	2013	Article	Excluded	No human population investigated
139	Naqvi et al[209]	Quantification of polychlorinated biphenyl contamination using human placenta as biomarker from Punjab Province, Pakistan	2018	Article	Excluded	No exposure to e-waste
140	Newman et al[210]	Investigation of Childhood Lead Poisoning from Parental Take-Home Exposure from an Electronic Scrap Recycling Facility — Ohio, 2012	2015	Report	Excluded	Report
141	Ni et al[211]	A review of human exposure to polybrominated diphenyl ethers (PBDEs) in China	2013	Review	Excluded	Review article
142	Ni et al[212]	Hair mercury concentrations and associated factors in an electronic waste recycling area, Guiyu, China	2013	Article	Excluded	No health outcomes reported
143	Noel-Brune et al[213]	Health effects of exposure to e-waste	2013	Correspondence	Excluded	Correspondence
144	Nyarku et al[214]	Schoolchildren's personal exposure to ultrafine particles in and near Accra, Ghana	2019	Article	Excluded	No health outcomes reported
145	Obiri et al[215]	Exposure to toxicants in soil and bottom ash deposits in Agbogbloshie, Ghana: human health risk assessment	2016	Article	Excluded	No human population investigated
146	Oguri et al[216]	Exposure assessment of heavy metals in an e-waste processing area in northern Vietnam	2018	Article	Excluded	No human population investigated
147	Ohajinwa et al[217]	Health risks of polybrominated diphenyl ethers (PBDEs) and metals at informal electronic waste recycling sites	2019	Article	Excluded	No human population investigated
148	May et al[218]	Prevalence and injury patterns among electronic waste workers in the informal sector in Nigeria	2018	Article	Excluded	No health outcomes reported
149	Okeme et al[219]	Electronic Waste Recycling: Occupational Exposures and Work-Related Health Effects	2019	Review	Excluded	Review article
150	Ouyang et al[220]	Non-carcinogenic health risk assessment of nickel in agricultural products and drinking water in an e-waste dismantling area of Qingyuan City, Guangdong Province	2019	Chinese article	Excluded	Chinese article
151	Owumi et al[221]	Electronic waste in Nigeria: Potential for genotoxicity and metalloid induced carcinogenesis	2013	Abstract	Excluded	Abstract
152	Pagano et al[222]	Human exposures to rare earth elements: Present knowledge and research prospects	2019	Review	Excluded	Review article
153	Pascale et al[223]	E-Waste Informal Recycling: An Emerging Source of Lead Exposure in South America	2016	Article	Excluded	No health outcomes reported
154	Peng et al[224]	Consumption of rice and fish in an electronic waste recycling area contributes significantly to total daily intake of mercury	2015	Article	Excluded	No health outcomes reported
155	Poole et al[225]	Systematic Review: Occupational illness in the waste and recycling sector	2017	Review	Excluded	Review article

156	Potera, C[226]	Roadmap for children's health: Controlling diverse environmental exposures in Latin America	2015	News	Excluded	News
157	Puangprasert, S Prueksasit, T[227]	Health risk assessment of airborne Cd, Cu, Ni and Pb for electronic waste dismantling workers in Buriram Province, Thailand	2019	Article	Excluded	No human population investigated
158	Qiao et al[228]	Legacy and Currently Used Organic Contaminants in Human Hair and Hand Wipes of Female E-Waste Dismantling Workers and Workplace Dust in South China	2019	Article	Excluded	No health outcomes reported
159	Qin et al[229]	Air pollution and body burden of persistent organic pollutants at an electronic waste recycling area of China	2019	Review	Excluded	Review article
160	Qu et al[230]	Comprehensive assessment of exposure to identify health consequences of e-waste	2014	Correspondence	Excluded	Correspondence
161	Ramírez-Hernández et al[231]	Environmental risks and children's health in a Mayan community from southeast of Mexico	2018	Article	Excluded	No exposure to e-waste
162	Schecter et al[232]	Biomonitoring of Metals, Polybrominated Diphenyl Ethers, Polychlorinated Biphenyls, and Persistent Pesticides in Vietnamese Female Electronic Waste Recyclers	2018	Article	Excluded	No health outcomes reported
163	Scruggs et al[233]	Improving information flow on chemicals in electronic products and E-waste to minimize negative consequences for health and the environment	2016	Article	Excluded	No health outcomes reported
164	Seeberger et al[234]	Special Report: E-Waste Management in the United States and Public Health Implications	2016	Report	Excluded	Report
165	Seith et al[235]	Self-Reported Health and Metal Body Burden in an Electronic Waste Recycling Community in Northeastern Thailand	2019	Article	Excluded	No health outcomes reported
166	Shang et al[236]	Bioaccumulation of PCDD/Fs, PCBs and PBDEs by earthworms in field soils of an E-waste dismantling area in China	2013	Article	Excluded	No human population investigated
167	Sharma, DC[237]	Emissions from e-waste recycling threaten workers' health	2015	Spotlight	Excluded	Spotlight
168	Shen et al[238]	Occurrence of two novel triazine-based flame retardants in an E-waste recycling area in South China: Implication for human exposure	2019	Article	Excluded	No human population investigated
169	Shi et al[239]	The health concern of polychlorinated biphenyls (PCBs) in a notorious e-waste recycling site	2019	Article	Excluded	No human population investigated
170	Shi et al[240]	Health risks of polycyclic aromatic hydrocarbons via fish consumption in Haimen bay (China), downstream of an e-waste recycling site (Guiyu)	2016	Article	Excluded	No human population investigated
171	Shi et al[241]	Short-term variability in levels of urinary phosphate flame retardant metabolites in adults and children from an e-waste recycling site	2016	Article	Excluded	No health outcomes reported

172	Shi et al[242]	Legacy and emerging brominated flame retardants in China: A review on food and human milk contamination, human dietary exposure and risk assessment	2018	Review	Excluded	Review article
173	Singh et al[243]	Health risk assessment of the workers exposed to the heavy metals in e-waste recycling sites of Chandigarh and Ludhiana, Punjab, India	2018	Article	Excluded	No human population investigated
174	Song, Q Li, J[244]	A systematic review of the human body burden of e-waste exposure in China	2014	Review	Excluded	Review article
175	Song, Q Li, J[245]	Environmental effects of heavy metals derived from the e-waste recycling activities in China: A systematic review	2014	Review	Excluded	Review article
176	Song, Q Li, J[246]	A review on human health consequences of metals exposure to e-waste in China	2015	Review	Excluded	Review article
177	Song et al[247]	Multivariate linear regression model for source apportionment and health risk assessment of heavy metals from different environmental media	2018	Article	Excluded	No human population investigated
178	Srigboh et al[248]	Multiple elemental exposures amongst workers at the Agbogbloshie electronic waste (e-waste) site in Ghana	2016	Article	Excluded	No health outcomes reported
179	Ssebugere et al[249]	Human and environmental exposure to PCDD/Fs and dioxin-like PCBs in Africa: A review	2019	Review	Excluded	Review article
180	Sthiannopkao, S Wong, MH[250]	Handling e-waste in developed and developing countries: Initiatives, practices, and consequences	2013	Article	Excluded	No health outcomes reported
181	Su et al[251]	A novel approach to stimulate the biphenyl-degrading potential of bacterial community from PCBs-contaminated soil of e-waste recycling sites	2013	Article	Excluded	No human population investigated
182	Surenderan, S Murkunde, Y[252]	Elucidation of Acute and Neurobehavioral Toxicity of E-Waste Extracts with Special Reference to Cognitive Impairment, Anxiety and Stress Response Using Zebrafish	2019	Chinese Article	Excluded	Chinese article
183	Tang et al[253]	Polychlorinated biphenyls and their methylsulfonyl metabolites in fish from an electronic waste recycling site in south China: tissue distribution and human dietary exposure	2014	Article chinese	Excluded	Chinese article
184	Tang et al[254]	Distribution of polybrominated diphenyl ethers in breast milk, cord blood and placentas: a systematic review	2017	Review	Excluded	Review article
185	Tang et al[255]	Mercury levels and estimated total daily intakes for children and adults from an electronic waste recycling area in Taizhou, China: Key role of rice and fish consumption	2015	Article	Excluded	No health outcomes reported
186	Tang et al[256]	Polybrominated diphenyl ethers (PBDEs) and heavy metals in road dusts from a plastic waste recycling area in north China: implications for human health	2016	Article	Excluded	No human population investigated

187	Tao et al[257]	Emerging Halogenated Flame Retardants and Hexabromocyclododecanes in Food Samples From an E-Waste Processing Area in Vietnam	2016	Article	Excluded	No health outcomes reported
188	Tao et al[258]	Bioaccessibility and health risk of heavy metals in ash from the incineration of different e-waste residues	2015	Article	Excluded	No human population investigated
189	The Lancet, Child Adolescent, Health[259]	Pollution: think of the children	2017	Editorial	Excluded	Editorial
190	Thompson, LA Darwish, WS[260]	Environmental Chemical Contaminants in Food: Review of a Global Proble	2019	Review	Excluded	Review article
191	Tokumaru et al[261]	Determination of the Extent of Trace Metals Pollution in Soils, Sediments and Human Hair at e-Waste Recycling Site in Ghana	2017	Article	Excluded	No health outcome
192	Tsamo, C[262]	E-waste assessment in Cameroon. Case study: Town of Maroua	2014	Case study	Excluded	Case study
193	Tue et al[263]	Dioxin-related compounds in breast milk of women from Vietnamese e-waste recycling sites: Levels, toxic equivalents and relevance of non-dietary exposure	2014	Article	Excluded	No health outcomes reported
194	Tue et al[264]	Environmental contamination and human exposure to dioxin-related compounds in e-waste recycling sites of developing countries	2013	Review	Excluded	Review article
195	Tue et al[265]	Contamination of indoor dust and air by polychlorinated biphenyls and brominated flame retardants and relevance of non-dietary exposure in Vietnamese informal e-waste recycling sites	2013	Article	Excluded	No human population investigated
196	Vaccari et al[266]	WEEE treatment in developing countries: Environmental pollution and health consequences—An overview	2019	Review	Excluded	Review article
197	Velis et al[267]	Unsound waste management and public health: The neglected link?	2016	Editorial	Excluded	Editorial
198	Velmurugan et al[268]	Gut Microbiota, Endocrine-Disrupting Chemicals, and the Diabetes Epidemic	2017	Review	Excluded	Review article
199	Vimalraj et al[269]	MicroRNAs: Impaired vasculogenesis in metal induced teratogenicity	2017	Review	Excluded	Review article
200	Wang et al[270]	Occupational exposure to polybrominated diphenyl ethers or decabromodiphenyl ethane during chemical manufacturing: Occurrence and health risk assessment	2019	Article	Excluded	No health outcomes reported
201	Wang et al[271]	Human health risk assessment of occupational and residential exposures to dechlorane plus in the manufacturing facility area in China and comparison with e-waste recycling site	2013	Article	Excluded	No human population investigated
202	Wang et al[272]	Study on genomic stability of male workers in an e-waste dismantling area in Tianjin	2019	Article chinese	Excluded	Chinese article
203	Wang et al[273]	Study on the exposure of polychlorinated biphenyl contamination and DNA methylation in male employees in an e-waste dismantling area in Tianjin	2019	Article chinese	Excluded	Chinese article

204	Wang et al[274]	Distribution of polybrominated diphenyl ethers in wild crucian carp and exposure estimation of dietary intake	2014	Article chinese	Excluded	Chinese article
205	Wang et al[275]	Synthetic Phenolic Antioxidants and Their Metabolites in Indoor Dust from Homes and Microenvironments	2016	Article	Excluded	No human population investigated
206	Wang et al[276]	Health risk assessment of migrant workers' exposure to polychlorinated biphenyls in air and dust in an e-waste recycling area in China: Indication for a new wealth gap in environmental rights	2016	Article	Excluded	No health outcomes reported
207	White ety al[277]	Exposure Potential and Health Impacts of Indium and Gallium, Metals Critical to Emerging Electronics and Energy Technologies	2016	Review	Excluded	Review article
208	Wittsiepe et al[278]	Pilot study on the internal exposure to heavy metals of informal-level electronic waste workers in Agbogbloshie, Accra, Ghana	2017	Article	Excluded	No health outcomes reported
209	Wittsiepe et al[279]	Levels of polychlorinated dibenzo-p-dioxins, dibenzofurans (PCDD/Fs) and biphenyls (PCBs) in blood of informal e-waste recycling workers from Agbogbloshie, Ghana, and controls	2015	Article	Excluded	No health outcomes reported
210	Wolansky, MJ[280]	From pesticide product RandD and registration to completion of a knowledge base on health risks by exposure to pesticide formulations: What should scientists do to protect public health in the interim?	2016	Abstract	Excluded	Abstract
211	Woo et al[281]	Potential resource and toxicity impacts from metals in waste electronic devices	2016	Article	Excluded	No human population investigated
212	Wu et al[282]	Spatial characteristics of cadmium in topsoils in a typical e-waste recycling area in southeast China and its potential threat to shallow groundwater	2014	Article	Excluded	No human population investigated
213	Wu et al[283]	Dermal Uptake from Airborne Organics as an Important Route of Human Exposure to E-Waste Combustion Fumes	2016	Review	Excluded	Review article
214	Wu et al[284]	Hepatic ethoxyresorufin-O-deethylase induction in the common kingfisher from an electronic waste recycling site	2016	Article	Excluded	No human population investigated
215	Wu et al[285]	Sex-dependent accumulation and maternal transfer of Dechlorane Plus flame retardant in fish from an electronic waste recycling site in South China	2013	Article	Excluded	No human population investigated
216	Wu et al[286]	Trace metals in e-waste lead to serious health risk through consumption of rice growing near an abandoned e-waste recycling site: Comparisons with PBDEs and AHFRs	2019	Article	Excluded	No human population investigated

217	Wu et al[286]	Trace metals in e-waste lead to serious health risk through consumption of rice growing near an abandoned e-waste recycling site: Comparisons with PBDEs and AHFRs	2019	Article	Excluded	No human population investigated
218	Xu et al[287]	Characterization of heavy metals and brominated flame retardants in the indoor and outdoor dust of e-waste workshops: implication for on-site human exposure	2015	Article	Excluded	No human population investigated
219	Xu et al[288]	Chaotic time series prediction for prenatal exposure to polychlorinated biphenyls in umbilical cord blood using the least squares SEATR model	2016	Report	Excluded	Report
220	Xu et al[289]	Chromium exposure among children from an electronic waste recycling town of China	2015	Article	Excluded	No health outcomes reported
221	Xu et al[290]	E-waste environmental contamination and harm to public health in China	2015	Review	Excluded	Review article
222	Xu et al[291]	Increase male genital diseases morbidity linked to informal electronic waste recycling in Guiyu, China	2014	Article	Excluded	No health outcomes reported
223	Yan et al[292]	Liver and Kidney Function of E-waste Dismantling Workers and Potential Influencing Factors	2018	Chinese Article	Excluded	Chinese article
224	Yan et al[293]	Urinary metabolites of phosphate flame retardants in workers occupied with e-waste recycling and incineration	2018	Article	Excluded	No health outcomes reported
225	Yang et al[294]	Multiple-life-stage probabilistic risk assessment for the exposure of Chinese population to PBDEs and risk managements	2018	Article	Excluded	No human population investigated
226	Yang et al[295]	Exposure to typical persistent organic pollutants from an electronic waste recycling site in Northern China	2013	Article	Excluded	No health outcomes reported
227	Yedla, S[296]	Development of a methodology for electronic waste estimation: A material flow analysis-based SYE-Waste Model	2016	Article	Excluded	No human population investigated
228	Yekeen et al[297]	Assessment of health risk of trace metal pollution in surface soil and road dust from e-waste recycling area in China	2016	Article	Excluded	No human population investigated
229	Yin et al[298]	Distribution Characteristics and Health Risk Assessment of Heavy Metals in a Soil-Rice System in an E-waste Dismantling Area	2018	Chinese article	Excluded	Chinese article
230	Yohannessen et al[299]	Health assessment of electronic waste workers in chile: Participant characterization	2019	Article	Excluded	No health outcomes reported
231	Yu et al[300]	Informal processing of electronic waste at Agbogbloshie, Ghana: workers' knowledge about associated health hazards and alternative livelihoods	2017	Article	Excluded	No health outcomes reported
232	Yu, G de Boer, J[301]	BFR2015 in Beijing: Scientists are becoming more concerned about FRs in indoor environment	2017	Editorial	Excluded	Editorial

233	Yu et al[302]	Thermal treatment of flame retardant plastics: A case study on a waste TV plastic shell sample	2019	Article	Excluded	No human population investigated
234	Yu et al[303]	Comments on "Polybrominated diphenyl ethers in foodstuffs from Taiwan: Level and human dietary exposure assessment" by Chen and co-authors	2013	Comment	Excluded	Comment
235	Yu et al[304]	Health implication of heavy metals exposure via multiple pathways for residents living near a former e-waste recycling area in China: A comparative study	2019	Article	Excluded	No human population investigated
236	Zeng et al[305]	Children with health impairments by heavy metals in an e-waste recycling area	2016	Review	Excluded	Review article
237	Zeng et al[306]	Lung function and respiratory symptoms in children from an electronic waste recycling area in China	2015	Abstract	Excluded	Abstract
238	Zeng et al[307]	Polychlorinated biphenyls and chlorinated paraffins in home-produced eggs from an e-waste polluted area in South China: Occurrence and human dietary exposure	2018	Article	Excluded	No human population investigated
239	Zeng et al[308]	Species-Specific Bioaccumulation of Halogenated Organic Pollutants and Their Metabolites in Fish Serum from an E-Waste Site, South China	2014	Article	Excluded	No human population investigated
240	Zhan et al[309]	Assessment of heavy metals exposure, noise and thermal safety in the ambiance of a vacuum metallurgy separation system for recycling heavy metals from crushed e-wastes	2014	Article	Excluded	No human population investigated
241	Zhang et al[310]	Concentrations of bisphenol A and its alternatives in paired maternal–fetal urine, serum and amniotic fluid from an e-waste dismantling area in China	2020	Article	Excluded	No health outcomes reported
242	Zhang et al[311]	PCB contamination in soils of the Pearl River Delta, South China: Levels, sources, and potential risks	2013	Article	Excluded	No human population investigated
243	Zhang et al[312]	Occupational exposure characteristics and health risk of PBDEs at different domestic e-waste recycling workshops in China	2019	Article	Excluded	No human population investigated
244	Zhang et al[313]	Risk assessment of polychlorinated biphenyls and heavy metals in soils of an abandoned e-waste site in China	2014	Article	Excluded	No human population investigated
245	Zhang et al[314]	Lead contamination in Chinese surface soils: Source identification, spatial-temporal distribution and associated health risks	2019	Review	Excluded	Review article
246	Zhang et al[315]	Blood lead levels among Chinese children: The shifting influence of industry, traffic, and e-waste over three decades	2020	Review	Excluded	Review article
247	Zhao et al[316]	Polybrominated diphenyl ethers (PBDEs) in aborted human fetuses and placental transfer during the first trimester of pregnancy	2013	Article	Excluded	No health outcomes reported

248	Zheng et al[317]	Association between lung function in school children and exposure to three transition metals from an e-waste recycling area	2013	Article	Excluded	2012 article
249	Zheng et al[318]	Polybrominated diphenyl ethers (PBDEs) in paired human hair and serum from e-waste recycling workers: Source apportionment of hair PBDEs and relationship between hair and serum	2014	Article	Excluded	No health outcomes reported
250	Zheng et al[319]	Heavy metals in food, house dust, and water from an e-waste recycling area in South China and the potential risk to human health	2013	Article	Excluded	No human population investigated
251	Zheng et al[320]	Polychlorinated biphenyls in human hair at an e-waste site in China: Composition profiles and chiral signatures in comparison to dust	2013	Article	Excluded	No health outcomes reported
252	Zheng et al[321]	Polychlorinated Biphenyls (PCBs) in Human Hair and Serum from E-Waste Recycling Workers in Southern China: Concentrations, Chiral Signatures, Correlations, and Source Identification	2016	Article	Excluded	No health outcomes reported
253	Zheng et al[322]	Flame retardants and organochlorines in indoor dust from several e-waste recycling sites in South China: Composition variations and implications for human exposure	2015	Article	Excluded	No human population investigated
254	Zheng et al[323]	Halogenated flame retardants during egg formation and chicken embryo development: Maternal transfer, possible biotransformation, and tissue distribution	2014	Article	Excluded	No human population investigated
255	Zhou et al[324]	S100P is a potential molecular target of cadmium-induced inhibition of human placental trophoblast cell proliferation	2016	Article	Excluded	In vitro experiment
256	Zhu et al[325]	Polychlorinated biphenyls in house dust at an e-waste site and urban site in the Pearl River Delta, southern China: sources and human exposure and health risks	2014	Chinese Article	Excluded	Chinese article
257	Zimmermann et al[326]	Occupational exposure in the fluorescent lamp recycling sector in France	2014	Article	Excluded	No human population investigated
258	Chen et al[327]	Chronic co-exposure to low levels of brominated flame retardants and heavy metals induces reproductive toxicity in zebrafish	2018	Article	Excluded	No human population investigated
259	Araujo et al[328]	Generation of domestic waste electrical and electronic equipment on Fernando de Noronha Island: qualitative and quantitative aspects	2017	Article	Excluded	No health outcome
260	Asante et al[329]	E-waste interventions in Ghana	2016	Article	Excluded	No health outcome
261	Cao et al[330]	Health risk assessment of various metal(loid)s via multiple exposure pathways on children living near a typical lead-acid battery plant, China	2015	Article	Excluded	No health outcome
262	Cesaro et al[331]	A relative risk assessment of the open burning of WEEE	2019	Review	Excluded	Review article
263	Gerić et al[332]	Environmental risk assessment of wastewaters from printed circuit board production: A multibiomarker approach using human cells	2017	Article	Excluded	In vitro experiment

264	Koike et al[333]	Penta- and octa-bromodiphenyl ethers promote proinflammatory protein expression in human bronchial epithelial cells in vitro	2014	Article	Excluded	In vitro experiment
265	Lu et al[334]	Directly repurposing waste optical discs with prefabricated nanogrooves as a platform for investigation of cell-substrate interactions and guiding neuronal growth	2018	Article	Excluded	In vitro experiment
266	Magalini, Federico[335]	Global challenges for e-waste management: the societal implications	2016	Article	Excluded	No health outcome
267	McAllister et al[336]	Women, e-waste, and technological solutions to climate change	2014	Article	Excluded	No health outcome
268	Mogharabi et al[337]	Toxicity of nanomaterials; an undermined issue	2014	Editorial	Excluded	Editorial
269	Ni et al[338]	A review of human exposure to polybrominated diphenyl ethers (PBDEs) in China	2013	Review	Excluded	Review article
270	Ohajinwa et al[339]	Health Risks Awareness of Electronic Waste Workers in the Informal Sector in Nigeria	2017	Article	Excluded	No health outcome
271	Ohajinwa et al[340]	Impact of informal electronic waste recycling on metal concentrations in soils and dusts	2018	Article	Excluded	No human population investigated
272	Perkins et al[341]	E-waste: a global hazard	2014	Review	Excluded	Review article
273	Song et al[342]	Environmental risk assessment of CRT and PCB workshops in a mobile e-waste recycling plant	2015	Article	Excluded	No human population investigated
274	Vaccari et al[343]	WEEE Treatment in Developing Countries: Environmental Pollution and Health Consequences-An Overview	2019	Review	Excluded	Review article
275	Vojta et al[344]	Screening for halogenated flame retardants in European consumer products, building materials and wastes	2017	Article	Excluded	No health outcome
276	Mishra, Sapna[345]	Perceived and Manifested Health Problems among Informal E-waste Handlers: A Scoping Review	2019	Review/PMC	Excluded	Review article
277	Afonso, JC[346]	Waste Electrical and Electronic Equipment: The Anthropocene Knocks on Our Door	2018	Review	Excluded	Review article
278	Chandrakant, SS[347]	IMAPACT OF E- WASTE ON ENVIRONMENT, HUMAN HEALTH AND EMPLOYMENT- A REVIEW	2018	Review	Excluded	Review article

References

1. Huo X, Wu Y, Xu L, Zeng X, Qin Q, Xu X. Maternal urinary metabolites of PAHs and its association with adverse birth outcomes in an intensive e-waste recycling area. *Environ Pollut* 2019; **245**: 453-61.
2. Li M, Huo X, Pan Y, Cai H, Dai Y, Xu X. Proteomic evaluation of human umbilical cord tissue exposed to polybrominated diphenyl ethers in an e-waste recycling area. *Environ Int* 2018: 362-71.
3. Xu L, Ge J, Huo X, Zhang Y, Lau ATY, Xu X. Differential proteomic expression of human placenta and fetal development following e-waste lead and cadmium exposure in utero. *Sci Total Environ* 2016; **550**: 1163-70.
4. Xu L, Huo X, Zhang Y, Li W, Zhang J, Xu X. Polybrominated diphenyl ethers in human placenta associated with neonatal physiological development at a typical e-waste recycling area in China. *Environ Pollut* 2015; **196**: 414-22.
5. Zhang Y, Xu X, Chen A, et al. Maternal urinary cadmium levels during pregnancy associated with risk of sex-dependent birth outcomes from an e-waste pollution site in China. *Reprod Toxicol* 2018; **75**: 49-55.
6. Xu X, Liu J, Huang C, Lu F, Chiung YM, Huo X. Association of polycyclic aromatic hydrocarbons (PAHs) and lead co-exposure with child physical growth and development in an e-waste recycling town. *Chemosphere* 2015; **139**: 295-302.
7. Yang H, Huo X, Yekeen TA, Zheng Q, Zheng M, Xu X. Effects of lead and cadmium exposure from electronic waste on child physical growth. *Environ Sci Pollut Res* 2013; **20**(7): 4441-7.
8. Zeng X, Xu X, Qin Q, Ye K, Wu W, Huo X. Heavy metal exposure has adverse effects on the growth and development of preschool children. *Environ Geochem Health* 2019; **41**(1): 309-21.
9. Cai H, Xu X, Zhang Y, Cong X, Lu X, Huo X. Elevated lead levels from e-waste exposure are linked to sensory integration difficulties in preschool children. *Neurotoxicology* 2019; **71**: 150-8.
10. Liu L, Xu X, Yekeen TA, Lin K, Li W, Huo X. Assessment of association between the dopamine D2 receptor (DRD2) polymorphism and neurodevelopment of children exposed to lead. *Environ Sci Pollut Res Int* 2015; **22**(3): 1786-93.
11. Liu W, Huo X, Liu D, Zeng X, Zhang Y, Xu X. S100β in heavy metal-related child attention-deficit hyperactivity disorder in an informal e-waste recycling area. *Neurotoxicology* 2014; **45**: 185-91.
12. Zhang R, Huo X, Ho G, et al. Attention-deficit/hyperactivity symptoms in preschool children from an e-waste recycling town: assessment by the parent report derived from DSM-IV. *BMC Pediatr* 2015; **15**(1): 1-8.
13. Liu L, Zhang B, Lin K, Zhang Y, Xu X, Huo X. Thyroid disruption and reduced mental development in children from an informal e-waste recycling area: A mediation analysis. *Chemosphere* 2018; **193**: 498-505.
14. Lv QX, Wang W, Li XH, Yu L, Zhang Y, Tian Y. Polychlorinated biphenyls and polybrominated biphenyl ethers in adipose tissue and matched serum from an E-waste recycling area (Wenling, China). *Environ Pollut* 2015; **199**: 219-26.
15. Ben YJ, Li XH, Yang YL, et al. Placental transfer of dechlorane plus in mother-infant pairs in an E-waste recycling area (Wenling, China). *Environ Sci Technol* 2014; **48**(9): 5187-93.
16. Zheng MY, Li XH, Zhang Y, Yang YL, Wang WY, Tian Y. Partitioning of polybrominated biphenyl ethers from mother to fetus and potential health-related implications. *Chemosphere* 2017; **170**: 207-15.
17. Xu X, Yekeen TA, Xiao Q, Wang Y, Lu F, Huo X. Placental IGF-1 and IGFBP-3 expression correlate with umbilical cord blood PAH and PBDE levels from prenatal exposure to electronic waste. *Environ Pollut* 2013; **182**: 63-9.
18. Xu X, Liu J, Zeng X, Lu F, Chen A, Huo X. Elevated serum polybrominated diphenyl ethers and alteration of thyroid hormones in children from Guiyu, China. *PLoS One* 2014; **9**(11): e113699.
19. Xu P, Lou X, Ding G, et al. Association of PCB, PBDE and PCDD/F body burdens with hormone levels for children in an e-waste dismantling area of Zhejiang Province, China. *Sci Total Environ* 2014; **499**(1): 55-61.
20. Eguchi A, Kunisue T, Wu Q, et al. Occurrence of Perchlorate and thiocyanate in human serum from e-waste recycling and reference sites in Vietnam: Association with thyroid hormone and iodide levels. *Arch Environ Contam Toxicol* 2014; **67**(1): 29-41.
21. Eguchi A, Nomiyama K, Minh Tue N, et al. Residue profiles of organohalogen compounds in human serum from e-waste recycling sites in North Vietnam: Association with thyroid hormone levels. *Environ Res* 2015; **137**: 440-9.
22. Xu P, Lou X, Ding G, et al. Effects of PCBs and PBDEs on thyroid hormone, lymphocyte proliferation, hematology and kidney injury markers in residents of an e-waste dismantling area in Zhejiang, China. *Sci Total Environ* 2015; **536**: 215-22.
23. Guo LC, Yu S, Wu D, et al. Disruption of thyroid hormone regulated proteins and gene expression by polychlorinated biphenyls, polybrominated diphenyl ethers and new flame retardants in residents of an e-waste region. *Environ Pollut* 2019; **254**: 112925.

24. Zheng J, He CT, Chen SJ, et al. Disruption of thyroid hormone (TH) levels and TH-regulated gene expression by polybrominated diphenyl ethers (PBDEs), polychlorinated biphenyls (PCBs), and hydroxylated PCBs in e-waste recycling workers. *Environ Int* 2017; **102**: 138-44.
25. Yang Y, Lu XS, Li DL, Yu YJ. Effects of environmental lead pollution on blood lead and sex hormone levels among occupationally exposed group in An E-waste dismantling area. *Biomed Environ Sci* 2013; **26**(6): 474-84.
26. Guo LC, Pan S, Yu S, et al. Human Sex Hormone Disrupting Effects of New Flame Retardants and Their Interactions with Polychlorinated Biphenyls, Polybrominated Diphenyl Ethers, a Case Study in South China. *Environ Sci Technol* 2018; **52**(23): 13935-41.
27. Zhou X, Ju Y, Wu Z, Yang K. Disruption of sex hormones and oxidative homeostasis in parturient women and their matching fetuses at an e-waste recycling site in China. *Int J Occup Environ Health* 2013; **19**(1): 22-8.
28. Cao J, Xu X, Zhang Y, Zeng Z, Hylkema MN, Huo X. Increased memory T cell populations in Pb-exposed children from an e-waste-recycling area. *Sci Total Environ* 2018; **616-617**: 988-95.
29. Huo X, Dai Y, Yang T, Zhang Y, Li M, Xu X. Decreased erythrocyte CD44 and CD58 expression link e-waste Pb toxicity to changes in erythrocyte immunity in preschool children. *Sci Total Environ* 2019; **664**: 690-7.
30. Zhang Y, Huo X, Cao J, Yang T, Xu L, Xu X. Elevated lead levels and adverse effects on natural killer cells in children from an electronic waste recycling area. *Environ Pollut* 2016; **213**: 143-50.
31. Lin S, Huo X, Zhang Q, et al. Short Placental Telomere was Associated with Cadmium Pollution in an Electronic Waste Recycling Town in China. *PLoS One* 2013; **8**(4): e60815.
32. Zeng Z, Huo X, Zhang Y, Hylkema MN, Wu Y, Xu X. Differential DNA methylation in newborns with maternal exposure to heavy metals from an e-waste recycling area. *Environ Res* 2019; **171**: 536-45.
33. Huo X, Peng L, Qiu B, Zheng L, Yekeen TA, Xu X. ALAD genotypes and blood lead levels of neonates and children from e-waste exposure in Guiyu, China. *Environ Sci Pollut Res Int* 2014; **21**(10): 6744-50.
34. Xu L, Huo X, Liu Y, Zhang Y, Qin Q, Xu X. Hearing loss risk and DNA methylation signatures in preschool children following lead and cadmium exposure from an electronic waste recycling area. *Chemosphere* 2020; **246**: 125829.
35. Li K, Liu S, Yang Q, et al. Genotoxic effects and serum abnormalities in residents of regions proximal to e-waste disposal facilities in Jinghai, China. *Ecotoxicol Environ Saf* 2014; **105**(1): 51-8.
36. Jinghua Y, Juan W, Yuxia Z, et al. Long-term Persistent Organic Pollutants Exposure Induced Telomere Dysfunction and Senescence-Associated Secretary Phenotype. *J Gerontol A Biol Sci Med Sci* 2018; **73**(8): 1027-35.
37. Li Z, Guo C, Li X, et al. Associations between metal exposure and global DNA methylation in potentially affected people in E-Waste recycling sites in Taizhou City, China. *Sci Total Environ* 2019; **711**: 135100.
38. He X, Jing Y, Wang J, et al. Significant accumulation of persistent organic pollutants and dysregulation in multiple DNA damage repair pathways in the electronic-waste-exposed populations. *Environ Res* 2015; **137**: 458-66.
39. Ni W, Huang Y, Wang X, Zhang J, Wu K. Associations of neonatal lead, cadmium, chromium and nickel co-exposure with DNA oxidative damage in an electronic waste recycling town. *Sci Total Environ* 2014; **472**: 354-62.
40. Xu X, Liao W, Lin Y, Dai Y, Shi Z, Huo X. Blood concentrations of lead, cadmium, mercury and their association with biomarkers of DNA oxidative damage in preschool children living in an e-waste recycling area. *Environ Geochem Health* 2018; **40**(4): 1481-94.
41. Li R, Yang Q, Qiu X, et al. Reactive oxygen species alteration of immune cells in local residents at an electronic waste recycling site in northern china. *Environ Sci Technol* 2013; **47**(7): 3344-52.
42. Lu SY, Li YX, Zhang JQ, et al. Associations between polycyclic aromatic hydrocarbon (PAH) exposure and oxidative stress in people living near e-waste recycling facilities in China. *Environ Int* 2016; **94**: 161-9.
43. Lu SY, Li YX, Zhang T, et al. Effect of E-waste Recycling on Urinary Metabolites of Organophosphate Flame Retardants and Plasticizers and Their Association with Oxidative Stress. *Environ Sci Technol* 2017; **51**(4): 2427-37.
44. Yang Q, Qiu X, Li R, Ma J, Li K, Li G. Polycyclic aromatic hydrocarbon (PAH) exposure and oxidative stress for a rural population from the North China Plain. *Environ Sci Pollut Res Int* 2015; **22**(3): 1760-9.
45. Zhang B, Zhang T, Duan Y, et al. Human exposure to phthalate esters associated with e-waste dismantling: Exposure levels, sources, and risk assessment. *Environ Int* 2019; **124**: 1-9.
46. Zhang T, Ruan J, Zhang B, et al. Heavy metals in human urine, foods and drinking water from an e-waste dismantling area: Identification of exposure sources and metal-induced health risk. *Ecotoxicol Environ Saf* 2019; **169**: 707-13.
47. Zhang T, Xue J, Gao CZ, et al. Urinary Concentrations of Bisphenols and Their Association with Biomarkers of Oxidative Stress in People Living Near E-Waste Recycling Facilities in China. *Environ Sci Technol* 2016; **50**(7): 4045-53.

48. Zeng X, Xu X, Boezen HM, Vonk JM, Wu W, Huo X. Decreased lung function with mediation of blood parameters linked to e-waste lead and cadmium exposure in preschool children. *Environ Pollut* 2017; **230**: 838-48.
49. Zeng X, Xu X, Zhang Y, Li W, Huo X. Chest circumference and birth weight are good predictors of lung function in preschool children from an e-waste recycling area. *Environ Sci Pollut Res Int* 2017; **24**(28): 22613-21.
50. Zeng X, Xu X, Zheng X, Reponen T, Chen A, Huo X. Heavy metals in PM2.5 and in blood, and children's respiratory symptoms and asthma from an e-waste recycling area. *Environ Pollut* 2016; **210**: 346-53.
51. Zhang S, Huo X, Zhang Y, Huang Y, Zheng X, Xu X. Ambient fine particulate matter inhibits innate airway antimicrobial activity in preschool children in e-waste areas. *Environ Int* 2019; **123**: 535-42.
52. Lu X, Xu X, Zhang Y, Zhang Y, Wang C, Huo X. Elevated inflammatory Lp-PLA2 and IL-6 link e-waste Pb toxicity to cardiovascular risk factors in preschool children. *Environ Pollut* 2018; **234**: 601-9.
53. Zheng X, Huo X, Zhang Y, Wang Q, Zhang Y, Xu X. Cardiovascular endothelial inflammation by chronic coexposure to lead (Pb) and polycyclic aromatic hydrocarbons from preschool children in an e-waste recycling area. *Environ Pollut* 2019; **246**: 587-96.
54. Cong X, Xu X, Xu L, et al. Elevated biomarkers of sympatho-adrenomedullary activity linked to e-waste air pollutant exposure in preschool children. *Environ Int* 2018; **115**: 117-26.
55. Gangwar C, Choudhari R, Chauhan A, Kumar A, Singh A, Tripathi A. Assessment of air pollution caused by illegal e-waste burning to evaluate the human health risk. *Environ Int* 2019; **125**: 191-9.
56. Burns KN, Sun K, Fobil JN, Neitzel RL. Heart rate, stress, and occupational noise exposure among electronic waste recycling workers. *Int J Environ Res Public Health* 2016; **13**(1).
57. Dai Y, Huo X, Zhang Y, Yang T, Li M, Xu X. Elevated lead levels and changes in blood morphology and erythrocyte CR1 in preschool children from an e-waste area. *Sci Total Environ* 2017; **592**: 51-9.
58. Zeng Z, Huo X, Zhang Y, Xiao Z, Zhang Y, Xu X. Lead exposure is associated with risk of impaired coagulation in preschool children from an e-waste recycling area. *Environ Sci Pollut Res Int* 2018; **25**(21): 20670-9.
59. Zhang Y, Xu X, Sun D, Cao J, Zhang Y, Huo X. Alteration of the number and percentage of innate immune cells in preschool children from an e-waste recycling area. *Ecotoxicol Environ Saf* 2017; **145**: 615-22.
60. Dai Y, Huo X, Cheng Z, Wang Q, Zhang Y, Xu X. Alterations in platelet indices link polycyclic aromatic hydrocarbons toxicity to low-grade inflammation in preschool children. *Environ Int* 2019; **131**: 105043.
61. Chen Y, Xu X, Zeng Z, Lin X, Qin Q, Huo X. Blood lead and cadmium levels associated with hematological and hepatic functions in patients from an e-waste-polluted area. *Chemosphere* 2019; **220**: 531-8.
62. Lin X, Xu X, Zeng X, Xu L, Zeng Z, Huo X. Decreased vaccine antibody titers following exposure to multiple metals and metalloids in e-waste-exposed preschool children. *Environ Pollut* 2017; **220**: 354-63.
63. Lin Y, Xu X, Dai Y, Zhang Y, Li W, Huo X. Considerable decrease of antibody titers against measles, mumps, and rubella in preschool children from an e-waste recycling area. *Sci Total Environ* 2016; **573**: 760-6.
64. Xu X, Chen X, Zhang J, et al. Decreased blood hepatitis B surface antibody levels linked to e-waste lead exposure in preschool children. *J Hazard Mater* 2015; **298**: 122-8.
65. Liu Y, Huo X, Xu L, et al. Hearing loss in children with e-waste lead and cadmium exposure. *Sci Total Environ* 2018; **624**: 621-7.
66. Zhang B, Huo X, Xu L, et al. Elevated lead levels from e-waste exposure are linked to decreased olfactory memory in children. *Environ Pollut* 2017; **231**: 1112-21.
67. Yu YJ, Lin BG, Liang WB, et al. Associations between PBDEs exposure from house dust and human semen quality at an e-waste areas in South China–A pilot study. *Chemosphere* 2018; **198**: 266-73.
68. Wang Y, Sun X, Fang L, et al. Genomic instability in adult men involved in processing electronic waste in Northern China. *Environ Int* 2018; **117**: 69-81.
69. Hou R, Huo X, Zhang S, Xu C, Huang Y, Xu X. Elevated levels of lead exposure and impact on the anti-inflammatory ability of oral sialic acids among preschool children in e-waste areas. *Sci Total Environ* 2020; **699**: 134380.
70. Song S, Duan Y, Zhang T, et al. Serum concentrations of bisphenol A and its alternatives in elderly population living around e-waste recycling facilities in China: Associations with fasting blood glucose. *Ecotoxicol Environ Saf* 2019; **169**: 822-8.
71. Abou-Elwafa Abdallah M. Environmental occurrence, analysis and human exposure to the flame retardant tetrabromobisphenol-A (TBBP-A)-A review. *Environ Int* 2016; **94**: 235-50.
72. Abul Kalam Azad M, Islam M, Ismail Hossin M. Generation of electronic-waste and its impact on environment and public health in Malaysia. *Ann Trop Med PH* 2017; **10**(5): 1123-7.
73. Ackah M. Informal E-waste recycling in developing countries: review of metal(loid)s pollution, environmental impacts and transport pathways. *Environ Sci Pollut Res Int* 2017; **24**(31): 24092-101.
74. Ackah M. Soil elemental concentrations, geoaccumulation index, non-carcinogenic and carcinogenic risks in functional areas of an informal e-waste recycling area in Accra, Ghana. *Chemosphere* 2019; **235**: 908-17.

75. Akortia E, Olukunle OI, Daso AP, Okonkwo JO. Soil concentrations of polybrominated diphenyl ethers and trace metals from an electronic waste dump site in the Greater Accra Region, Ghana: Implications for human exposure. *Ecotoxicol Environ Saf* 2017; **137**: 247-55.
76. Amankwaa EF, Adovor Tsikudo KA, Bowman J. 'Away' is a place: The impact of electronic waste recycling on blood lead levels in Ghana. *Sci Total Environ* 2017; **601-602**: 1566-74.
77. An T, Huang Y, Li G, He Z, Chen J, Zhang C. Pollution profiles and health risk assessment of VOCs emitted during e-waste dismantling processes associated with different dismantling methods. *Environ Int* 2014; **73**: 186-94.
78. Andersen JCØ, Cropp A, Paradise DC. Solubility of indium-tin oxide in simulated lung and gastric fluids: Pathways for human intake. *Sci Total Environ* 2017; **579**: 628-36.
79. Anh HQ, Nam VD, Tri TM, et al. Polybrominated diphenyl ethers in plastic products, indoor dust, sediment and fish from informal e-waste recycling sites in Vietnam: a comprehensive assessment of contamination, accumulation pattern, emissions, and human exposure. *Environ Geochem Health* 2017; **39**(4): 935-54.
80. Annamalai J. Occupational health hazards related to informal recycling of E-waste in India: An overview. *Indian J Occup Environ Med* 2015; **19**(1): 61-5.
81. Arain AL, Neitzel RL. A review of biomarkers used for assessing human exposure to metals from E-waste. *Int J Environ Res Public Health* 2019; **16**(10).
82. Asamoah A, Essumang DK, Muff J, Kucheryavskiy SV, Søgaard EG. Assessment of PCBs and exposure risk to infants in breast milk of primiparae and multiparae mothers in an electronic waste hot spot and non-hot spot areas in Ghana. *Sci Total Environ* 2018; **612**: 1473-9.
83. Asamoah A, Nikbakht Fini M, Essumang DK, Muff J, Søgaard EG. PAHs contamination levels in the breast milk of Ghanaian women from an e-waste recycling site and a residential area. *Sci Total Environ* 2019; **666**: 347-54.
84. Awasthi AK, Wang M, Awasthi MK, Wang Z, Li J. Environ pollut and human body burden from improper recycling of e-waste in China: A short-review. *Environ pollut* 2018; **243**: 1310-6.
85. Awasthi AK, Zeng X, Li J. Relationship between e-waste recycling and human health risk in India: a critical review. *Environ Sci Pollut Res Int*2016; **23**(12): 11509-32.
86. Awasthi AK, Zeng X, Li J. Environ pollut of electronic waste recycling in India: A critical review. *Environ pollut* 2016; **211**: 259-70.
87. Bai XY, Lu SY, Xie L, et al. A pilot study of metabolites of organophosphorus flame retardants in paired maternal urine and amniotic fluid samples: Potential exposure risks of tributyl phosphate to pregnant women. *Environ Sci Process Impacts* 2019; **21**(1): 124-32.
88. Basu N, Ayelo PA, Djogbénou LS, et al. Occupational and Environmental Health Risks Associated with Informal Sector Activities-Selected Case Studies from West Africa. *New Solut* 2016; **26**(2): 253-70.
89. Becker GE, Ryan-Fogarty Y. Environmental impact of bottles, teats, and packaging in maternity units. *The BMJ* 2019; **367**.
90. Ben YJ, Li XH, Yang YL, et al. Dechlorane Plus and its dechlorinated analogs from an e-waste recycling center in maternal serum and breast milk of women in Wenling, China. *Environ pollut* 2013; **173**: 176-81.
91. Borthakur A. Health and Environmental Hazards of Electronic Waste in India. *J Environ Health* 2016; **78**(8): 18-23.
92. Bruce-Vanderpuije P, Megson D, Jobst K, et al. Background levels of dioxin-like polychlorinated biphenyls (dlPCBs), polychlorinated, polybrominated and mixed halogenated dibenzo-p-dioxins and dibenzofurans (PCDD/Fs, PBDD/Fs & PXDD/Fs) in sera of pregnant women in Accra, Ghana. *Sci Total Environ* 2019; **673**: 631-42.
93. Budnik LT, Casteleyn L. Mercury pollution in modern times and its socio-medical consequences. *Sci Total Environ* 2019; **654**: 720-34.
94. Burns KN, Sayler SK, Neitzel RL. Stress, health, noise exposures, and injuries among electronic waste recycling workers in Ghana. *J Occup Med Toxicol* 2019; **14**(1).
95. Cabrera-Rodríguez R, Luzardo OP, González-Antuña A, et al. Occurrence of 44 elements in human cord blood and their association with growth indicators in newborns. *Environ Int* 2018; **116**: 43-51.
96. Cai T, Luo W, Ruan D, Wu YJ, Fox DA, Chen J. The history, status, gaps, and future directions of neurotoxicology in China. *Environ Health Perspect* 2016; **124**(6): 722-32.
97. Cao P, Fujimori T, Juhasz A, Takaoka M, Oshita K. Bioaccessibility and human health risk assessment of metal(loid)s in soil from an e-waste open burning site in Agbogbloshie, Accra, Ghana. *Chemosphere* 2020; **240**.
98. Caudle WM. Occupational Metal Exposure and Parkinsonism. 2017. p. 143-58.
99. Ceballos D, Beaucham C, Page E. Metal Exposures at three U.S. electronic scrap recycling facilities. *J Occup Environ Hyg* 2017; **14**(6): 401-8.
100. Ceballos DM, Dong Z. The formal electronic recycling industry: Challenges and opportunities in occupational and environmental health research. *Environ Int* 2016; **95**: 157-66.

101. Cesaro A, Belgiorno V, Vaccari M, et al. A device-specific prioritization strategy based on the potential for harm to human health in informal WEEE recycling. *Environ Sci Pollut Res Int* 2018; **25**(1): 683-92.
102. Chakraborty P. Modeling the emission sources for polychlorinated biphenyls in India: Implications for human health risk assessment. *Rev Environ Health* 2014; **29**(1-2): 37-40.
103. Chakraborty P, Selvaraj S, Nakamura M, Prithiviraj B, Cincinelli A, Bang JJ. PCBs and PCDD/Fs in soil from informal e-waste recycling sites and open dumpsites in India: Levels, congener profiles and health risk assessment. *Sci Total Environ* 2018; **621**: 930-8.
104. Ceballos DM, Gong W, Page E. A Pilot Assessment of Occupational Health Hazards in the US Electronic Scrap Recycling Industry. *J Occup Environ Hyg* 2015; **12**(7): 482-8.
105. Chan JKY, Man YB, Wu SC, Wong MH. Dietary intake of PBDEs of residents at two major electronic waste recycling sites in China. *Sci Total Environ* 2013; **463-464**: 1138-46.
106. Chan JKY, Man YB, Xing GH, et al. Dietary exposure to polychlorinated dibenzo-p-dioxins and dibenzofurans via fish consumption and dioxin-like activity in fish determined by H4IIE-luc bioassay. *Sci Total Environ* 2013; **463-464**: 1192-200.
107. Chan JKY, Wong MH. A review of environmental fate, body burdens, and human health risk assessment of PCDD/Fs at two typical electronic waste recycling sites in China. *Sci Total Environ* 2013; **463-464**: 1111-23.
108. Chen H, Lam JCW, Zhu M, et al. Combined Effects of Dust and Dietary Exposure of Occupational Workers and Local Residents to Short- and Medium-Chain Chlorinated Paraffins in a Mega E-Waste Recycling Industrial Park in South China. *Environ Sci Technol* 2018; **52**(20): 11510-9.
109. Chen H, Ma S, Yu Y, et al. Seasonal profiles of atmospheric PAHs in an e-waste dismantling area and their associated health risk considering bioaccessible PAHs in the human lung. *Sci Total Environ* 2019; **683**: 371-9.
110. Chen J, Huang Y, Li G, An T, Hu Y, Li Y. VOCs elimination and health risk reduction in e-waste dismantling workshop using integrated techniques of electrostatic precipitation with advanced oxidation technologies. *J Hazard Mater* 2016; **302**: 395-403.
111. Chen K, Zheng J, Yan X, et al. Dechlorane Plus in paired hair and serum samples from e-waste workers: Correlation and differences. *Chemosphere* 2015; **123**: 43-7.
112. Chen XC, Zhu XH, Lin BG, et al. Children's non-carcinogenic health risk assessment of heavy metals exposure to residential indoor dust around an e-waste dismantling area in South China. *Zhonghua yu fang yi xue za zhi [Chinese journal of preventive medicine]* 2019; **53**(4): 360-4.
113. Cole C, Gnanapragasam A, Cooper T, Singh J. An assessment of achievements of the WEEE Directive in promoting movement up the waste hierarchy: experiences in the UK. *Waste Manag* 2019; **87**: 417-27.
114. Dartey E, Berlinger B, Weinbruch S, et al. Essential and non-essential trace elements among working populations in Ghana. *J Trace Elem Med Biol* 2017; **44**: 279-87.
115. Davis JM, Garb Y. A strong spatial association between e-waste burn sites and childhood lymphoma in the West Bank, Palestine. *nt J Cancer* 2019; **144**(3): 470-5.
116. Decharat S. Urinary Mercury Levels Among Workers in E-waste Shops in Nakhon Si Thammarat Province, Thailand. *J Prev Med Public Health* 2018; **51**(4): 196-204.
117. Déportes I, Fangeat E, Desqueyroux H. Potential health impacts of waste electrical and electronic equipment management: A brief comparison between emerging and developed countries. *Environnement, Risques et Sante* 2018; **17**(1): 57-64.
118. Devi NL, Yadav IC, Chakraborty P, Shihua Q. Polychlorinated Biphenyls in Surface Soil from North-East India: Implication for Sources Apportionment and Health-Risk Assessment. *Archives of Environmental Contamination and Toxicology* 2018; **75**(3): 377-89.
119. Die Q, Nie Z, Huang Q, et al. Concentrations and occupational exposure assessment of polybrominated diphenyl ethers in modern Chinese e-waste dismantling workshops. *Chemosphere* 2019; **214**: 379-88.
120. Dowling R, Feola G, Laborde A, Gualtero S, Hernandez L. Reducing blood lead levels in children exposed to electronic waste recycling in Montevideo. *Ann Glob Health* 2016; 2016. p. 442.
121. Du Y, Wang Y, Du L, et al. Cytogenetics alteration in adult men involved in the recycling of electronic wastes. *Environ Int* 2018; **112**: 174-82.
122. Esogwah BC, Ana G, Bolaji O. Hepatotoxic effects of electronic waste leachate on rats. *Clin Chem Lab Med* 2014; **52**: S1677.
123. Etzel RA. Environmental hazards that matter for children's health. *Hong Kong J Paediatr* 2015; **20**(2): 86-94.
124. Fang W, Yang Y, Xu Z. PM10 and PM2.5 and health risk assessment for heavy metals in a typical factory for cathode ray tube television recycling. *Environ Sci Technol* 2013; **47**(21): 12469-76.
125. Feldt T, Fobil JN, Wittsiepe J, et al. High levels of PAH-metabolites in urine of e-waste recycling workers from Agbogbloshie, Ghana. *Sci Total Environ* 2014; **466-467**: 369-76.
126. Foullon Aguilera M, Mejía Saucedo R, Calderón Hernández J, Yáñez-Estrada L. Assessment of exposure to persistent organic pollutants and alteration on thyroid hormone levels in Mexican children from a community dedicated to electronic waste recycling. *Toxicol Lett* 2016; **259**: S118.

127. Fu J, Zhang A, Wang T, et al. Influence of E-waste dismantling and its regulations: Temporal trend, spatial distribution of heavy metals in rice grains, and its potential health risk. *Environ Sci Technol* 2013; **47**(13): 7437-45.
128. Giudice LC. Environmental toxicants: hidden players on the reproductive stage. *Fertility and Sterility* 2016; **106**(4): 791-4.
129. Gomathi N, Rupesh PL, Sridevi L. Study of E-waste- hazards & recycling techniques- A review. *Int J Chemtech Res* 2015; **8**(11): 300-7.
130. González-Antuña A, Camacho M, Henríquez-Hernández LA, et al. Simultaneous quantification of 49 elements associated to e-waste in human blood by ICP-MS for routine analysis. *MethodsX* 2017; **4**: 328-34.
131. Grant K, Goldizen FC, Sly PD, et al. Health consequences of exposure to e-waste: A systematic review. *Lancet Glob Health* 2013; **1**(6): e350-e61.
132. Gravel S, Aubin S, Labrèche F. Assessment of Occupational Exposure to Organic Flame Retardants: A Systematic Review. *Ann Work Expo Health* 2019; **63**(4): 386-406.
133. Gravel S, Bakhiyi B, Lavoué J, Verner MA, Zayed J, Labrèche F. Electronic waste recycling exposure and hormone levels in workers. *Occup Environ Med* 2019; **76**: A58-A9.
134. Gravel S, Côté D, Gladu S, Labrèche F. Electronic waste recycling in quÉbec, canada: Hiring practices and occupational health and safety management. *Occup Environ Med* 2019; **76**: A11.
135. Guo P, Xu X, Huang B, et al. Blood lead levels and associated factors among children in Guiyu of China: A population-based study. *PLoS One* 2014; **9**(8).
136. Gwenzi W, Mangori L, Danha C, Chaukura N, Dunjana N, Sanganyado E. Sources, behaviour, and environmental and human health risks of high-technology rare earth elements as emerging contaminants. *Sci Total Environ* 2018; **636**: 299-313.
137. Ha TT, Burwell ST, Goodwin ML, Noeker JA, Heggland SJ. Pleiotropic roles of Ca+2/calmodulin-dependent pathways in regulating cadmium-induced toxicity in human osteoblast-like cell lines. *Toxicol Lett* 2016; **260**: 18-27.
138. Hahladakis JN, Stylianos M, Gidarakos E. Assessment of released heavy metals from electrical and electronic equipment (EEE) existing in shipwrecks through laboratory-scale simulation reactor. *J Hazard Mater* 2013; **250-251**: 256-64.
139. Han W, Gao G, Geng J, Li Y, Wang Y. Ecological and health risks assessment and spatial distribution of residual heavy metals in the soil of an e-waste circular economy park in Tianjin, China. *Chemosphere* 2018; **197**: 325-35.
140. He CT, Zheng J, Qiao L, et al. Occurrence of organophosphorus flame retardants in indoor dust in multiple microenvironments of southern China and implications for human exposure. *Chemosphere* 2015; **133**: 47-52.
141. He CT, Zheng XB, Yan X, et al. Organic contaminants and heavy metals in indoor dust from e-waste recycling, rural, and urban areas in South China: Spatial characteristics and implications for human exposure. *Ecotoxicol Environ Saf* 2017; **140**: 109-15.
142. Heacock M, Kelly CB, Asante KA, et al. E-waste and harm to vulnerable populations: A growing global problem. *Environ Health Perspect* 2016; **124**(5): 550-5.
143. Heacock M, Kelly CB, Suk WA. E-waste: the growing global problem and next steps. *Rev Environ Health* 2016; **31**(1): 131-5.
144. Hennig B. Protective influence of healthful nutrition on mechanisms of environmental pollutant toxicity and disease risks. *Chinese Medicine (United Kingdom)* 2018; **13**.
145. Hennig B, Petriello MC, Gamble MV, et al. The role of nutrition in influencing mechanisms involved in environmentally mediated diseases. *Rev Environ Health* 2018; **33**(1): 87-97.
146. Henríquez-Hernández LA, Boada LD, Carranza C, et al. Blood levels of toxic metals and rare earth elements commonly found in e-waste may exert subtle effects on hemoglobin concentration in sub-Saharan immigrants. *Environ Int* 2017; **109**: 20-8.
147. Henríquez-Hernández LA, Luzardo OP, Boada LD, et al. Study of the influencing factors of the blood levels of toxic elements in Africans from 16 countries. *Environ pollut* 2017; **230**: 817-28.
148. Henríquez-Hernández LA, Romero D, González-Antuña A, et al. Biomonitoring of 45 inorganic elements measured in plasma from Spanish subjects: A cross-sectional study in Andalusian population. *Sci Total Environ* 2020; **706**.
149. Hou R, Huo X, Zhang S, Xu C, Huang Y, Xu X. Corrigendum to "Elevated levels of lead exposure and impact on the anti-inflammatory ability of oral sialic acids among preschool children in e-waste areas" (Sci Total Environ (2020) 699, (S0048969719343712), (10.1016/j.scitotenv.2019.134380)). *Sci Total Environ* 2020; **705**.
150. Hu J, Xiao X, Peng P, Huang W, Chen D, Cai Y. Spatial distribution of polychlorinated dibenzo-p-dioxins and dibenzo-furans (PCDDs/Fs) in dust, soil, sediment and health risk assessment from an intensive electronic waste recycling site in Southern China. *Environ Sci Process Impacts* 2013; **15**(10): 1889-96.

151. Huang CL, Bao LJ, Luo P, Wang ZY, Li SM, Zeng EY. Potential health risk for residents around a typical e-waste recycling zone via inhalation of size-fractionated particle-bound heavy metals. *J Hazard Mater* 2016; **317**: 449-56.
152. Huang J, Nkrumah PN, Anim DO, Mensah E. E-waste disposal effects on the aquatic environment: Accra, Ghana. 2014. p. 19-34.
153. Huang Y, Ni W, Chen Y, Wang X, Zhang J, Wu K. Levels and risk factors of antimony contamination in human hair from an electronic waste recycling area, Guiyu, China. *Environ Sci Pollut Res Int* 2015; **22**(9): 7112-9.
154. Huo X, Zheng XB, Liu Q, Zhang T, Wang QH, Xu XJ. Impact of informal e-waste recycling on human health. *Zhonghua yu fang yi xue za zhi [Chinese journal of preventive medicine]* 2019; **53**(4): 426-32.
155. Hussain M, Mumtaz S. E-waste: Impacts, issues and management strategies. *Rev Environ Health* 2014; **29**(1-2): 53-8.
156. Ibe FC, Opara AI, Ibe BO, Adindu BC, Ichu BC. Environmental and health implications of trace metal concentrations in street dusts around some electronic repair workshops in Owerri, Southeastern Nigeria. *Environ Monit Assess* 2018; **190**(12).
157. Ilankoon IMSK, Ghorbani Y, Chong MN, Herath G, Moyo T, Petersen J. E-waste in the international context – A review of trade flows, regulations, hazards, waste management strategies and technologies for value recovery. *Waste Manag* 2018; **82**: 258-75.
158. Iqbal M, Breivik K, Syed JH, et al. Emerging issue of e-waste in Pakistan: A review of status, research needs and data gaps. *Environ pollut* 2015; **207**: 308-18.
159. Iqbal M, Syed JH, Breivik K, et al. E-Waste Driven Pollution in Pakistan: The First Evidence of Environmental and Human Exposure to Flame Retardants (FRs) in Karachi City. *Environ Sci Technol* 2017; **51**(23): 13895-905.
160. Isara AR, Akinfenwa Y, Aigbokhaode AQ. Serum lipid profile and atherogenic indices of e-waste workers in benin city, nigeria. *Am J Respir Crit Care Med* 2018; **197**(MeetingAbstracts).
161. Jafarzadeh-Ghoushchi S, Dorosti S. Effects of exposure to a variety of waste on human health - A review. *JLUMHS* 2017; **16**(1): 3-9.
162. Jiang H, Lin Z, Wu Y, et al. Daily intake of polybrominated diphenyl ethers via dust and diet from an e-waste recycling area in China. *J Hazard Mater* 2014; **276**: 35-42.
163. Jiang Y, Yuan L, Lin Q, Ma S, Yu Y. Polybrominated diphenyl ethers in the environment and human external and internal exposure in China: A review. *Sci Total Environ* 2019; **696**.
164. Jibiri NN, Isinkaye MO, Momoh HA. Assessment of radiation exposure levels at Alaba e-waste dumpsite in comparison with municipal waste dumpsites in southwest Nigeria. *Radiat Res Appl Sci2* 014; **7**(4): 536-41.
165. Julander A, Lundgren L, Skare L, et al. Formal recycling of e-waste leads to increased exposure to toxic metals: an occupational exposure study from Sweden. *Environ Int* 2014; **73**: 243-51.
166. Kang DHP, Chen M, Ogunseitan OA. Potential environmental and human health impacts of rechargeable lithium batteries in electronic waste. *Environ Sci Technol* 2013; **47**(10): 5495-503.
167. Khan MU, Besis A, Li J, Zhang G, Malik RN. New insight into the distribution pattern, levels, and risk diagnosis of FRs in indoor and outdoor air at low- and high-altitude zones of Pakistan: Implications for sources and exposure. *Chemosphere* 2017; **184**: 1372-87.
168. Khlaif N, Qumsiyeh MB. Genotoxicity of recycled electronic waste in Idhna, Hebron District, occupied Palestinian territory: A case-controlled study. *The Lancet* 2018; **391**(SPEC.ISS 1): S22.
169. Kim JW, Tue NM, Isobe T, et al. Contamination by perfluorinated compounds in water near waste recycling and disposal sites in Vietnam. *Environ Monit Assess* 2013; **185**(4): 2909-19.
170. Kim S, Xu X, Zhang Y, et al. Metal concentrations in pregnant women and neonates from informal electronic waste recycling. *Journal of exposure science & environmental epidemiology* 2019; **29**(3): 406-15.
171. Klinčić D, Dvoršćak M, Jagić K, Mendaš G, Herceg Romanić S. Levels and distribution of polybrominated diphenyl ethers in humans and environmental compartments: a comprehensive review of the last five years of research. *Environ Sci Pollut Res Int* 2020.
172. Krishnamoorthy Y, M V, Sakthivel M, Sarveswaran G. Emerging public health threat of e-waste management: global and Indian perspective. *Rev Environ Health* 2018; **33**(4): 321-9.
173. Kuo LJ, Cade SE, Cullinan V, Schultz IR. Polybrominated diphenyl ethers (PBDEs) in plasma from E-waste recyclers, outdoor and indoor workers in the Puget Sound, WA region. *Chemosphere* 2019; **219**: 209-16.
174. Laborde A. Recycling of e-waste: An estimation of cumulative health risks posed to vulnerable populations through exposure to neurotoxicant mixtures. *Toxicol Lett* 2016; **259**: S38.
175. Laborde A, Tomasina F, Bianchi F, et al. Children's health in Latin America: The infuence of environmental exposures. *Environ Health Perspect* 2015; **123**(3): 201-9.
176. Labunska I, Abdallah MAE, Eulaers I, et al. Human dietary intake of organohalogen contaminants at e-waste recycling sites in Eastern China. *Environ Int* 2015; **74**: 209-20.

177. Labunska I, Harrad S, Santillo D, Johnston P, Yun L. Domestic duck eggs: An important pathway of human exposure to PBDEs around E-waste and scrap metal processing areas in Eastern China. *Environ Sci Technol* 2013; **47**(16): 9258-66.
178. Labunska I, Harrad S, Wang M, Santillo D, Johnston P. Human dietary exposure to PBDEs around E-waste recycling sites in Eastern China. *Environ Sci Technol* 2014; **48**(10): 5555-64.
179. Landrigan PJ, Sly JL, Ruchirawat M, et al. Health Consequences of Environmental Exposures: Changing Global Patterns of Exposure and Disease. *Ann Glob Health* 2016; **82**(1): 10-9.
180. Laskaris Z, Milando C, Batterman S, et al. Derivation of Time-Activity Data Using Wearable Cameras and Measures of Personal Inhalation Exposure among Workers at an Informal Electronic-Waste Recovery Site in Ghana. *Ann Work Expo Health* 2019; **63**(8): 829-41.
181. Lau WKY, Liang P, Man YB, Chung SS, Wong MH. Human health risk assessment based on trace metals in suspended air particulates, surface dust, and floor dust from e-waste recycling workshops in Hong Kong, China. *Environ Sci Pollut Res* 2014; **21**(5): 3813-25.
182. Lecler MT, Zimmermann F, Silvente E, Clerc F, Chollot A, Grosjean J. Exposure to hazardous substances in Cathode Ray Tube (CRT) recycling sites in France. *Waste Manag* 2015; **39**: 226-35.
183. Leyssens L, Vinck B, Van Der Straeten C, Wuyts F, Maes L. Cobalt toxicity in humans—A review of the potential sources and systemic health effects. *Toxicology* 2017; **387**: 43-56.
184. Li J, Li W, Gao X, et al. Occurrence of multiple classes of emerging photoinitiators in indoor dust from E-waste recycling facilities and adjacent communities in South China and implications for human exposure. *Environ Int* 2020; **136**.
185. Li WL, Qi H, Ma WL, et al. Occurrence, behavior and human health risk assessment of dechlorane plus and related compounds in indoor dust of China. *Chemosphere* 2015; **134**: 166-71.
186. Li X, Duan Y, Sun H, et al. Human exposure levels of PAEs in an e-waste recycling area: Get insight into impacts of spatial variation and manipulation mode. *Environ Int* 2019; **133**.
187. Li X, Tian Y, Zhang Y, Ben Y, Lv Q. Accumulation of polybrominated diphenyl ethers in breast milk of women from an e-waste recycling center in China. *J Environ Sci (China)* 2017; **52**: 305-13.
188. Liang S, Xu F, Tang W, et al. Brominated flame retardants in the hair and serum samples from an e-waste recycling area in southeastern China: the possibility of using hair for biomonitoring. *Environ Sci Pollut Res Int* 2016; **23**(15): 14889-97.
189. Lin M, Tang J, Ma S, et al. Insights into biomonitoring of human exposure to polycyclic aromatic hydrocarbons with hair analysis: A case study in e-waste recycling area. *Environ Int* 2020; **136**.
190. Liu DC, Xu XJ, Zheng XB, Jiang YS, Zhang JQ, Huo X. The study of exposure levels of dioxin-like compounds in cord blood of newborns in an e-waste dismantling area in Guangdong Province. *Zhonghua yu fang yi xue za zhi [Chinese journal of preventive medicine]* 2019; **53**(4): 365-70.
191. Liu R, Chen J, Li G, Wang X, An T. Cutting down on the ozone and SOA formation as well as health risks of VOCs emitted from e-waste dismantlement by integration technique. *J Environ Manage* 2019; **249**.
192. Liu R, Ma S, Li G, Yu Y, An T. Comparing pollution patterns and human exposure to atmospheric PBDEs and PCBs emitted from different e-waste dismantling processes. *J Hazard Mater* 2019; **369**: 142-9.
193. Luo P, Bao LJ, Guo Y, Li SM, Zeng EY. Size-dependent atmospheric deposition and inhalation exposure of particle-bound organophosphate flame retardants. *J Hazard Mater* 2016; **301**: 504-11.
194. Luo P, Bao LJ, Li SM, Zeng EY. Size-dependent distribution and inhalation cancer risk of particle-bound polycyclic aromatic hydrocarbons at a typical e-waste recycling and an urban site. *Environ pollut* 2015; **200**: 10-5.
195. Luo P, Bao LJ, Wu FC, Li SM, Zeng EY. Health risk characterization for resident inhalation exposure to particle-bound halogenated flame retardants in a typical e-waste recycling zone. *Environ Sci Technol* 2014; **48**(15): 8815-22.
196. Luzardo OP, Boada LD, Carranza C, et al. Socioeconomic development as a determinant of the levels of organochlorine pesticides and PCBs in the inhabitants of Western and Central African countries. *Sci Total Environ* 2014; **497-498**: 97-105.
197. Ma L. Effects of e-waste exposure on the synthesis of hemoglobin in preschool children. *Pediatr Blood Cancer* 2014; **61**: S291-S2.
198. Ma L, Wang TY, Chen YB. Effects of E-waste exposure on the synthesis of haemoglobin in preschool children. *Arch Dis Child* 2014; **99**: A112.
199. Ma S, Ren G, Zeng X, Yu Z, Sheng G, Fu J. Polychlorinated biphenyls and their hydroxylated metabolites in the serum of e-waste dismantling workers from eastern China. *Environ Geochem Health* 2018; **40**(5): 1931-40.
200. Malliari E, Kalantzi OI. Children's exposure to brominated flame retardants in indoor environments - A review. *Environ Int*2017; **108**: 146-69.
201. Man YB, Chow KL, Wang HS, et al. Human health risk assessment of soil dioxin/furans contamination and dioxin-like activity determined by ethoxyresorufin-O-deethylase bioassay. *Environ Sci Pollut Res Int*2015; **22**(7): 5218-27.

202. Man YB, Chow KL, Xing GH, Chan JKY, Wu SC, Wong MH. A pilot study on health risk assessment based on body loadings of PCBs of lactating mothers at Taizhou, China, the world's major site for recycling transformers. *Environ pollut* 2017; **227**: 364-71.
203. Man YB, Kang Y, Wang HS, et al. Cancer risk assessments of Hong Kong soils contaminated by polycyclic aromatic hydrocarbons. *J Hazard Mater* 2013; **261**: 770-6.
204. Matovu H, Sillanpää M, Ssebugere P. Polybrominated diphenyl ethers in mothers' breast milk and associated health risk to nursing infants in Uganda. *Sci Total Environ* 2019; **692**: 1106-15.
205. Méndez M, Battocletti A, Sosa A, Pose D, Moll MJ, Laborde A. Blood lead levels and potential sources of lead exposure among children in Montevideo, Uruguay. *Toxicol Lett* 2016; **259**: S170.
206. Mishra S. Perceived and manifested health problems among informal e-waste handlers: A scoping review. *Indian Journal of Occup Environ Med* 2019; **23**(1): 7-14.
207. Mishra S, Shamanna B, Kannan S. Exploring the Awareness Regarding E-waste and its Health Hazards among the Informal Handlers in Musheerabad Area of Hyderabad. *Indian Journal of Occup Environ Med* 2017; **21**(3): 143-8.
208. Mo L, Wu JP, Luo XJ, et al. Dechlorane Plus flame retardant in kingfishers (Alcedo atthis) from an electronic waste recycling site and a reference site, South China: Influence of residue levels on the isomeric composition. *Environ pollut* 2013; **174**: 57-62.
209. Naqvi A, Qadir A, Mahmood A, et al. Quantification of polychlorinated biphenyl contamination using human placenta as biomarker from Punjab Province, Pakistan. *Environ Sci Pollut Res Int* 2018; **25**(15): 14551-62.
210. Newman N, Jones C, Page E, Ceballos D, Oza A. Investigation of Childhood Lead Poisoning from Parental Take-Home Exposure from an Electronic Scrap Recycling Facility — Ohio, 2012. *MMWR Morbidity and mortality weekly report* 2015; **64**(27): 743-5.
211. Ni K, Lu Y, Wang T, et al. A review of human exposure to polybrominated diphenyl ethers (PBDEs) in China. *Int J Hyg Environ Health* 2013; **216**(6): 607-23.
212. Ni W, Chen Y, Huang Y, et al. Hair mercury concentrations and associated factors in an electronic waste recycling area, Guiyu, China. *Environ Res* 2013.
213. Noel-Brune M, Goldizen FC, Neira M, et al. Health effects of exposure to e-waste. *Lancet Glob* 2013; **1**(2): e70.
214. Nyarku M, Buonanno G, Ofosu F, Jayaratne R, Mazaheri M, Morawska L. Schoolchildren's personal exposure to ultrafine particles in and near Accra, Ghana. *Environ Int* 2019; **133**.
215. Obiri S, Ansa-Asare OD, Mohammed S, Darko HF, Dartey AG. Exposure to toxicants in soil and bottom ash deposits in Agbogbloshie, Ghana: human health risk assessment. *Environ Monit Assess* 2016; **188**(10).
216. Oguri T, Suzuki G, Matsukami H, et al. Exposure assessment of heavy metals in an e-waste processing area in northern Vietnam. *Sci Total Environ* 2018; **621**: 1115-23.
217. Ohajinwa CM, van Bodegom PM, Osibanjo O, et al. Health risks of polybrominated diphenyl ethers (PBDEs) and metals at informal electronic waste recycling sites. *Int J Environ Res Public Health* 2019; **16**(6).
218. Ohajinwa CM, van Bodegom PM, Vijver MG, Olumide AO, Osibanjo O, Peijnenburg WJGM. Prevalence and injury patterns among electronic waste workers in the informal sector in Nigeria. *Inj Prev* 2018; **24**(3): 185-92.
219. Okeme JO, Arrandale VH. Electronic Waste Recycling: Occupational Exposures and Work-Related Health Effects. *Curr Environ Health Rep* 2019; **6**(4): 256-68.
220. Ouyang JP, Song SM, Gao CZ, Gui MW, Zhang T. Non-carcinogenic health risk assessment of nickel in agricultural products and drinking water in an e-waste dismantling area of Qingyuan City, Guangdong Province. *Zhonghua yu fang yi xue za zhi [Chinese journal of preventive medicine]* 2019; **53**(4): 405-7.
221. Owumi SE, Gbadegesin MA, Osuagwu FC, et al. Electronic waste in Nigeria: Potential for genotoxicity and metalloid induced carcinogenesis. *Cancer Prevention Research* 2013; **6**(11).
222. Pagano G, Thomas PJ, Di Nunzio A, Trifuoggi M. Human exposures to rare earth elements: Present knowledge and research prospects. *Environ Res* 2019; **171**: 493-500.
223. Pascale A, Sosa A, Bares C, et al. E-Waste Informal Recycling: An Emerging Source of Lead Exposure in South America. *Ann Glob Health* 2016; **82**(1): 197-201.
224. Peng H, Reid MS, Le XC. Consumption of rice and fish in an electronic waste recycling area contributes significantly to total daily intake of mercury. *J Environ Sci (China)* 2015; **38**: 83-6.
225. Poole CJM, Basu S. Systematic Review: Occupational illness in the waste and recycling sector. *Occup Med (Lond)* 2017; **67**(8): 626-36.
226. Potera C. Roadmap for children's health: Controlling diverse environmental exposures in Latin America. *Environ Health Perspect* 2015; **123**(3): A70.
227. Puangprasert S, Prueksasit T. Health risk assessment of airborne Cd, Cu, Ni and Pb for electronic waste dismantling workers in Buriram Province, Thailand. *J Environ Manage* 2019; **252**.
228. Qiao L, Zheng XB, Zheng J, et al. Legacy and Currently Used Organic Contaminants in Human Hair and Hand Wipes of Female E-Waste Dismantling Workers and Workplace Dust in South China. *Environ Sci Technol* 2019; **53**(5): 2820-9.

229. Qin Q, Xu X, Dai Q, Ye K, Wang C, Huo X. Air pollution and body burden of persistent organic pollutants at an electronic waste recycling area of China. *Environmental geochemistry and health* 2019; **41**(1): 93-123.
230. Qu W, Zheng Y, Andersen ME, Zheng W, Rappaport SM. Comprehensive assessment of exposure to identify health consequences of e-waste. *Lancet Glob Health* 2014; **2**(2): e73.
231. Ramírez-Hernández H, Perera-Rios J, May-Euán F, Uicab-Pool G, Peniche-Lara G, Pérez-Herrera N. Environmental risks and children's health in a Mayan community from southeast of Mexico. *Ann Glob Health* 2018; **84**(2): 292-9.
232. Schecter A, Kincaid J, Quynh HT, et al. Biomonitoring of Metals, Polybrominated Diphenyl Ethers, Polychlorinated Biphenyls, and Persistent Pesticides in Vietnamese Female Electronic Waste Recyclers. *Journal of Occup Environ Med* 2018; **60**(2): 191-7.
233. Scruggs CE, Nimpuno N, Moore RBB. Improving information flow on chemicals in electronic products and E-waste to minimize negative consequences for health and the environment. *Resour Conserv Recycl* 2016; **113**: 149-64.
234. Seeberger J, Grandhi R, Kim SS, et al. Special Report: E-Waste Management in the United States and Public Health Implications. *J Environ Health* 2016; **79**(3): 8-16.
235. Seith R, Arain AL, Nambunmee K, Adar SD, Neitzel RL. Self-Reported Health and Metal Body Burden in an Electronic Waste Recycling Community in Northeastern Thailand. *J Occup Environ Med* 2019; **61**(11): 905-9.
236. Shang H, Wang P, Wang T, et al. Bioaccumulation of PCDD/Fs, PCBs and PBDEs by earthworms in field soils of an E-waste dismantling area in China. *Environ Int* 2013; **54**: 50-8.
237. Sharma DC. Emissions from e-waste recycling threaten workers' health. *Lancet Respir Med* 2015; **3**(11): 847-8.
238. Shen M, Ge J, Lam JCW, Zhu M, Li J, Zeng L. Occurrence of two novel triazine-based flame retardants in an E-waste recycling area in South China: Implication for human exposure. *Sci Total Environ* 2019; **683**: 249-57.
239. Shi J, Xiang L, Luan H, Wei Y, Ren H, Chen P. The health concern of polychlorinated biphenyls (PCBs) in a notorious e-waste recycling site. *Ecotoxicol Environ Saf* 2019; **186**.
240. Shi J, Zheng GJS, Wong MH, et al. Health risks of polycyclic aromatic hydrocarbons via fish consumption in Haimen bay (China), downstream of an e-waste recycling site (Guiyu). *Environ Res* 2016; **147**: 233-40.
241. Shi Y, Zheng X, Yan X, et al. Short-term variability in levels of urinary phosphate flame retardant metabolites in adults and children from an e-waste recycling site. *Chemosphere* 2019; **234**: 395-401.
242. Shi Z, Zhang L, Li J, Wu Y. Legacy and emerging brominated flame retardants in China: A review on food and human milk contamination, human dietary exposure and risk assessment. *Chemosphere* 2018; **198**: 522-36.
243. Singh M, Thind PS, John S. Health risk assessment of the workers exposed to the heavy metals in e-waste recycling sites of Chandigarh and Ludhiana, Punjab, India. *Chemosphere* 2018; **203**: 426-33.
244. Song Q, Li J. A systematic review of the human body burden of e-waste exposure in China. *Environ Int* 2014; **68**: 82-93.
245. Song Q, Li J. Environmental effects of heavy metals derived from the e-waste recycling activities in China: A systematic review. *Waste Manag*2014; **34**(12): 2587-94.
246. Song Q, Li J. A review on human health consequences of metals exposure to e-waste in China. *Environ Pollut* 2015; **196**: 450-61.
247. Song Y, Li H, Li J, et al. Multivariate linear regression model for source apportionment and health risk assessment of heavy metals from different environmental media. *Ecotoxicol Environ Saf* 2018; **165**: 555-63.
248. Srigboh RK, Basu N, Stephens J, et al. Multiple elemental exposures amongst workers at the Agbogbloshie electronic waste (e-waste) site in Ghana. *Chemosphere* 2016; **164**: 68-74.
249. Ssebugere P, Sillanpää M, Matovu H, Mubiru E. Human and environmental exposure to PCDD/Fs and dioxin-like PCBs in Africa: A review. *Chemosphere* 2019; **223**: 483-93.
250. Sthiannopkao S, Wong MH. Handling e-waste in developed and developing countries: Initiatives, practices, and consequences. *Sci Total Environ* 2013; **463-464**: 1147-53.
251. Su X, Shen H, Yao X, Ding L, Yu C, Shen C. A novel approach to stimulate the biphenyl-degrading potential of bacterial community from PCBs-contaminated soil of e-waste recycling sites. *Bioresour Technol* 2013; **146**: 27-34.
252. Surenderan S, Murkunde Y. Elucidation of Acute and Neurobehavioral Toxicity of E-Waste Extracts with Special Reference to Cognitive Impairment, Anxiety and Stress Response Using Zebrafish. *J Toxicol Environ* 2019; **11**(1): 27-35.
253. Tang B, Luo XJ, Zeng YH, Mai BX. Polychlorinated biphenyls and their methylsulfonyl metabolites in fish from an electronic waste recycling site in south China: tissue distribution and human dietary exposure. *Huan jing ke xue= Huanjing kexue / [bian ji, Zhongguo ke xue yuan huan jing ke xue wei yuan hui "Huan jing ke xue" bian ji wei yuan hui]* 2014; **35**(12): 4655-62.
254. Tang J, Zhai JX. Distribution of polybrominated diphenyl ethers in breast milk, cord blood and placentas: a systematic review. *Environ Sci Pollut Res Int* 2017; **24**(27): 21548-73.

255. Tang W, Cheng J, Zhao W, Wang W. Mercury levels and estimated total daily intakes for children and adults from an electronic waste recycling area in Taizhou, China: Key role of rice and fish consumption. *J Environ Sci (China)* 2015; **34**: 107-15.
256. Tang Z, Huang Q, Yang Y, et al. Polybrominated diphenyl ethers (PBDEs) and heavy metals in road dusts from a plastic waste recycling area in north China: implications for human health. *Environ Sci Pollut Res Int* 2016; **23**(1): 625-37.
257. Tao F, Matsukami H, Suzuki G, et al. Emerging halogenated flame retardants and hexabromocyclododecanes in food samples from an e-waste processing area in Vietnam. *Environ Sci Process Impacts* 2016; **18**(3): 361-70.
258. Tao XQ, Shen DS, Shentu JL, Long YY, Feng YJ, Shen CC. Bioaccessibility and health risk of heavy metals in ash from the incineration of different e-waste residues. *Environ Sci Pollut Res Int* 2015; **22**(5): 3558-69.
259. The Lancet C, Adolescent H. Pollution: think of the children. *Lancet Child Adolesc Health* 2017; **1**(4): 249.
260. Thompson LA, Darwish WS. Environmental Chemical Contaminants in Food: Review of a Global Problem. *J Toxicol* 2019; **2019**.
261. Tokumaru T, Ozaki H, Onwona-Agyeman S, Ofosu-Anim J, Watanabe I. Determination of the Extent of Trace Metals Pollution in Soils, Sediments and Human Hair at e-Waste Recycling Site in Ghana. *Arch Environ Contam Toxicol* 2017; **73**(3): 377-90.
262. Tsamo C. E-waste assessment in Cameroon. Case study: Town of Maroua. *Int J Chemtech Res* 2014; **6**(1): 681-90.
263. Tue NM, Katsura K, Suzuki G, et al. Dioxin-related compounds in breast milk of women from Vietnamese e-waste recycling sites: Levels, toxic equivalents and relevance of non-dietary exposure. *Ecotoxicol Environ Saf* 2014; **106**: 220-5.
264. Tue NM, Takahashi S, Subramanian A, Sakai S, Tanabe S. Environmental contamination and human exposure to dioxin-related compounds in e-waste recycling sites of developing countries. *Environ Sci Process Impacts* 2013; **15**(7): 1326-31.
265. Tue NM, Takahashi S, Suzuki G, et al. Contamination of indoor dust and air by polychlorinated biphenyls and brominated flame retardants and relevance of non-dietary exposure in Vietnamese informal e-waste recycling sites. *Environ Int* 2013; **51**: 160-7.
266. Vaccari M, Vinti G, Cesaro A, et al. WEEE treatment in developing countries: Environ pollut and health consequences—An overview. *Int J Environ Res Public Health* 2019; **16**(9).
267. Velis C, Mavropoulos A. Unsound waste management and public health: The neglected link? *Waste Manag Res* 2016; **34**(4): 277-9.
268. Velmurugan G, Ramprasath T, Gilles M, Swaminathan K, Ramasamy S. Gut Microbiota, Endocrine-Disrupting Chemicals, and the Diabetes Epidemic. *Trends Endocrinol Metab* 2017; **28**(8): 612-25.
269. Vimalraj S, Sumantran VN, Chatterjee S. MicroRNAs: Impaired vasculogenesis in metal induced teratogenicity. *Reprod Toxicol* 2017; **70**: 30-48.
270. Wang D, Chen T, Fu Z, et al. Occupational exposure to polybrominated diphenyl ethers or decabromodiphenyl ethane during chemical manufacturing: Occurrence and health risk assessment. *Chemosphere* 2019; **231**: 385-92.
271. Wang DG, Alaee M, Byer JD, Brimble S, Pacepavicius G. Human health risk assessment of occupational and residential exposures to dechlorane plus in the manufacturing facility area in China and comparison with e-waste recycling site. *Sci Total Environ* 2013; **445-446**: 329-36.
272. Wang JH, Wang Y, Du LQ, Xu C, Liu Q. Study on genomic stability of male workers in an e-waste dismantling area in Tianjin. *Zhonghua yu fang yi xue za zhi [Chinese journal of preventive medicine]* 2019; **53**(4): 371-5.
273. Wang JH, Wang Y, Du LQ, Xu C, Liu Q. Study on the exposure of polychlorinated biphenyl contamination and DNA methylation in male employees in an e-waste dismantling area in Tianjin. *Zhonghua yu fang yi xue za zhi [Chinese journal of preventive medicine]* 2019; **53**(4): 376-81.
274. Wang JX, Wang CY, Liu LL, Zhou XY, Liu YC, Lin KF. Distribution of polybrominated diphenyl ethers in wild crucian carp and exposure estimation of dietary intake. *Huan jing ke xue= Huanjing kexue / [bian ji, Zhongguo ke xue yuan huan jing ke xue wei yuan hui "Huan jing ke xue" bian ji wei yuan hui]* 2014; **35**(8): 3175-82.
275. Wang W, Asimakopoulos AG, Abualnaja KO, et al. Synthetic Phenolic Antioxidants and Their Metabolites in Indoor Dust from Homes and Microenvironments. *Environ Sci Technol* 2016; **50**(1): 428-34.
276. Wang Y, Hu J, Lin W, et al. Health risk assessment of migrant workers' exposure to polychlorinated biphenyls in air and dust in an e-waste recycling area in China: Indication for a new wealth gap in environmental rights. *Environ Int* 2016; **87**: 33-41.
277. White SJ, Shine JP. Exposure Potential and Health Impacts of Indium and Gallium, Metals Critical to Emerging Electronics and Energy Technologies. *Curr Environ Health Rep* 2016; **3**(4): 459-67.
278. Wittsiepe J, Feldt T, Till H, Burchard G, Wilhelm M, Fobil JN. Pilot study on the internal exposure to heavy metals of informal-level electronic waste workers in Agbogbloshie, Accra, Ghana. *Environ Sci Pollut Res Int* 2017; **24**(3): 3097-107.

279. Wittsiepe J, Fobil JN, Till H, Burchard GD, Wilhelm M, Feldt T. Levels of polychlorinated dibenzo-p-dioxins, dibenzofurans (PCDD/Fs) and biphenyls (PCBs) in blood of informal e-waste recycling workers from Agbogbloshie, Ghana, and controls. *Environ Int* 2015; **79**: 65-73.
280. Wolansky MJ. From pesticide product RandD and registration to completion of a knowledge base on health risks by exposure to pesticide formulations: What should scientists do to protect public health in the interim? *Toxicol Lett* 2016; **259**: S39.
281. Woo SH, Lee DS, Lim SR. Potential resource and toxicity impacts from metals in waste electronic devices. *Integr Environ Assess Manag* 2016; **12**(2): 364-70.
282. Wu C, Luo Y, Deng S, Teng Y, Song J. Spatial characteristics of cadmium in topsoils in a typical e-waste recycling area in southeast China and its potential threat to shallow groundwater. *Sci Total Environ* 2014; **472**: 556-61.
283. Wu CC, Bao LJ, Tao S, Zeng EY. Dermal Uptake from Airborne Organics as an Important Route of Human Exposure to E-Waste Combustion Fumes. *Environ Sci Technol* 2016; **50**(13): 6599-605.
284. Wu JP, Mo L, Zhi H, et al. Hepatic ethoxyresorufin-O-deethylase induction in the common kingfisher from an electronic waste recycling site. *Environ Toxicol Chem* 2016; **35**(6): 1594-9.
285. Wu JP, She YZ, Zhang Y, et al. Sex-dependent accumulation and maternal transfer of Dechlorane Plus flame retardant in fish from an electronic waste recycling site in South China. *Environ pollut* 2013; **177**: 150-5.
286. Wu Q, Leung JYS, Du Y, et al. Trace metals in e-waste lead to serious health risk through consumption of rice growing near an abandoned e-waste recycling site: Comparisons with PBDEs and AHFRs. *Environ pollut* 2019; **247**: 46-54.
287. Xu F, Liu Y, Wang J, et al. Characterization of heavy metals and brominated flame retardants in the indoor and outdoor dust of e-waste workshops: implication for on-site human exposure. *Environ Sci Pollut Res Int* 2015; **22**(7): 5469-80.
288. Xu X, Tang Q, Xia H, Zhang Y, Li W, Huo X. Chaotic time series prediction for prenatal exposure to polychlorinated biphenyls in umbilical cord blood using the least squares SEATR model. *Scientific reports* 2016; **6**: 25005.
289. Xu X, Yekeen TA, Liu J, Zhuang B, Li W, Huo X. Chromium exposure among children from an electronic waste recycling town of China. *Environ Sci Pollut Res Int* 2015; **22**(3): 1778-85.
290. Xu X, Zeng X, Boezen HM, Huo X. E-waste environmental contamination and harm to public health in China. *Front Med* 2015; **9**(2): 220-8.
291. 'Xu X, Zhang Y, Yekeen TA, Li Y, Zhuang B, Huo X. Increase male genital diseases morbidity linked to informal electronic waste recycling in Guiyu, China. *Environ Sci Pollut Res* 2014; **21**(5): 3540-5.
292. Yan X, Li SY, Wang MH, Xu RF, Zheng J, Ren MZ. Liver and Kidney Function of E-waste Dismantling Workers and Potential Influencing Factors. *Huan jing ke xue= Huanjing kexue* 2018; **39**(2): 953-60.
293. Yan X, Zheng X, Wang M, et al. Urinary metabolites of phosphate flame retardants in workers occupied with e-waste recycling and incineration. *Chemosphere* 2018; **200**: 569-75.
294. Yang J, Huang D, Zhang L, et al. Multiple-life-stage probabilistic risk assessment for the exposure of Chinese population to PBDEs and risk managements. *Sci Total Environ* 2018; **643**: 1178-90.
295. Yang Q, Qiu X, Li R, et al. Exposure to typical persistent organic pollutants from an electronic waste recycling site in Northern China. *Chemosphere* 2013; **91**(2): 205-11.
296. Yedla S. Development of a methodology for electronic waste estimation: A material flow analysis-based SYE-Waste Model. *Waste Manag Res* 2016; **34**(1): 81-6.
297. Yekeen TA, Xu X, Zhang Y, et al. Assessment of health risk of trace metal pollution in surface soil and road dust from e-waste recycling area in China. *Environ Sci Pollut Res Int* 2016; **23**(17): 17511-24.
298. Yin YM, Zhao WT, Huang T, Cheng SG, Zhao ZL, Yu CC. Distribution Characteristics and Health Risk Assessment of Heavy Metals in a Soil-Rice System in an E-waste Dismantling Area. *Huan jing ke xue= Huanjing kexue* 2018; **39**(2): 916-26.
299. Yohannessen K, Pinto-Galleguillos D, Parra-Giordano D, et al. Health assessment of electronic waste workers in chile: Participant characterization. I*nt J Environ Res Public Health* 2019; **16**(3).
300. Yu EA, Akormedi M, Asampong E, Meyer CG, Fobil JN. Informal processing of electronic waste at Agbogbloshie, Ghana: workers' knowledge about associated health hazards and alternative livelihoods. *Glob Health Promot* 2017; **24**(4): 90-8.
301. Yu G, de Boer J. BFR2015 in Beijing: Scientists are becoming more concerned about FRs in indoor environment. *Chemosphere* 2017; **174**: 664.
302. Yu S, Su W, Wu D, et al. Thermal treatment of flame retardant plastics: A case study on a waste TV plastic shell sample. *Sci Total Environ* 2019; **675**: 651-7.
303. Yu Y, Zhang X, Fu J. Comments on "Polybrominated diphenyl ethers in foodstuffs from Taiwan: Level and human dietary exposure assessment" by Chen and co-authors. *Sci Total Environ* 2013; **444**: 241-2.
304. Yu Y, Zhu X, Li L, et al. Health implication of heavy metals exposure via multiple pathways for residents living near a former e-waste recycling area in China: A comparative study. *Ecotoxicol Environ Saf* 2019; **169**: 178-84.

305. Zeng X, Xu X, Boezen HM, Huo X. Children with health impairments by heavy metals in an e-waste recycling area. *Chemosphere* 2016; **148**: 408-15.
306. Zeng X, Xu X, Boezen HM, et al. Lung function and respiratory symptoms in children from an electronic waste recycling area in China. *Eur Respir J* 2015; **46**.
307. Zeng Y, Huang C, Luo X, Liu Y, Ren Z, Mai B. Polychlorinated biphenyls and chlorinated paraffins in home-produced eggs from an e-waste polluted area in South China: Occurrence and human dietary exposure. *Environ Int* 2018; **116**: 52-9.
308. Zeng YH, Luo XJ, Zheng XB, Tang B, Wu JP, Mai BX. Species-Specific Bioaccumulation of Halogenated Organic Pollutants and Their Metabolites in Fish Serum from an E-Waste Site, South China. *Arch Environ Contam Toxicol* 2014; **67**(3): 348-57.
309. Zhan L, Xu Z. Assessment of heavy metals exposure, noise and thermal safety in the ambiance of a vacuum metallurgy separation system for recycling heavy metals from crushed e-wastes. *Waste Manag Res* 2014; **32**(12): 1247-53.
310. Zhang B, He Y, Zhu H, et al. Concentrations of bisphenol A and its alternatives in paired maternal–fetal urine, serum and amniotic fluid from an e-waste dismantling area in China. *Environ Int* 2020; **136**.
311. Zhang H, Luo Y, Teng Y, Wan H. PCB contamination in soils of the Pearl River Delta, South China: Levels, sources, and potential risks. *Environ Sci Pollut Res* 2013; **20**(8): 5150-9.
312. Zhang M, Shi J, Meng Y, et al. Occupational exposure characteristics and health risk of PBDEs at different domestic e-waste recycling workshops in China. *Ecotoxicol Environ Saf* 2019; **174**: 532-9.
313. Zhang Q, Ye J, Chen J, Xu H, Wang C, Zhao M. Risk assessment of polychlorinated biphenyls and heavy metals in soils of an abandoned e-waste site in China. *Environ pollut* 2014; **185**: 258-65.
314. Zhang Y, Hou D, O'Connor D, et al. Lead contamination in Chinese surface soils: Source identification, spatial-temporal distribution and associated health risks. *Critical Reviews in Environ Sci Technol* 2019; **49**(15): 1386-423.
315. Zhang Y, O'Connor D, Xu W, Hou D. Blood lead levels among Chinese children: The shifting influence of industry, traffic, and e-waste over three decades. *Environ Int* 2020; **135**.
316. Zhao Y, Ruan X, Li Y, Yan M, Qin Z. Polybrominated diphenyl ethers (PBDEs) in aborted human fetuses and placental transfer during the first trimester of pregnancy. *Environ Sci Technol* 2013; **47**(11): 5939-46.
317. Zheng G, Xu X, Li B, Wu K, Yekeen TA, Huo X. Association between lung function in school children and exposure to three transition metals from an e-waste recycling area. *J Expo Sci Environ Epidemiol* 2013; **23**(1): 67-72.
318. Zheng J, Chen KH, Luo XJ, et al. Polybrominated diphenyl ethers (PBDEs) in paired human hair and serum from e-waste recycling workers: Source apportionment of hair PBDEs and relationship between hair and serum. *Environ Sci Technol* 2014; **48**(1): 791-6.
319. Zheng J, Chen KH, Yan X, et al. Heavy metals in food, house dust, and water from an e-waste recycling area in South China and the potential risk to human health. *Ecotoxicol Environ Saf* 2013; **96**: 205-12.
320. Zheng J, Yan X, Chen SJ, et al. Polychlorinated biphenyls in human hair at an e-waste site in China: Composition profiles and chiral signatures in comparison to dust. *Environ Int* 2013; **54**: 128-33.
321. Zheng J, Yu LH, Chen SJ, et al. Polychlorinated Biphenyls (PCBs) in Human Hair and Serum from E-Waste Recycling Workers in Southern China: Concentrations, Chiral Signatures, Correlations, and Source Identification. *Environ Sci Technol* 2016; **50**(3): 1579-86.
322. Zheng X, Xu F, Chen K, et al. Flame retardants and organochlorines in indoor dust from several e-waste recycling sites in South China: Composition variations and implications for human exposure. *Environ Int* 2015; **78**: 1-7.
323. Zheng XB, Luo XJ, Zeng YH, Wu JP, Chen SJ, Mai BX. Halogenated flame retardants during egg formation and chicken embryo development: Maternal transfer, possible biotransformation, and tissue distribution. *Environ Toxicol Chem* 2014; **33**(8): 1712-9.
324. Zhou T, Wang H, Zhang S, Jiang X, Wei X. S100P is a potential molecular target of cadmium-induced inhibition of human placental trophoblast cell proliferation. *Exp Toxicol Pathol* 2016; **68**(10): 565-70.
325. Zhu ZC, Chen SJ, Ding N, Wang J, Luo XJ, Mai BX. Polychlorinated biphenyls in house dust at an e-waste site and urban site in the Pearl River Delta, southern China: sources and human exposure and health risks. *Huan jing ke xue= Huanjing kexue / [bian ji, Zhongguo ke xue yuan huan jing ke xue wei yuan hui "Huan jing ke xue" bian ji wei yuan hui]* 2014; **35**(8): 3066-72.
326. Zimmermann F, Lecler MT, Clerc F, Chollot A, Silvente E, Grosjean J. Occupational exposure in the fluorescent lamp recycling sector in France. *Waste Manag* 2014; **34**(7): 1257-63.
327. Chen J, Ma X, Tian L, et al. Chronic co-exposure to low levels of brominated flame retardants and heavy metals induces reproductive toxicity in zebrafish. *Toxicol Ind Health* 2018; **34**(9): 631-9.
328. Araujo DRR, de Oliveira JD, Selva VF, Silva MM, Santos SM. Generation of domestic waste electrical and electronic equipment on Fernando de Noronha Island: qualitative and quantitative aspects. *Environ Sci Pollut Res Int* 2017; **24**(24): 19703-13.

329. Asante KA, Pwamang JA, Amoyaw-Osei Y, Ampofo JA. E-waste interventions in Ghana. *Rev Environ Health* 2016; **31**(1): 145-8.
330. Cao S, Duan X, Zhao X, et al. Health risk assessment of various metal(loid)s via multiple exposure pathways on children living near a typical lead-acid battery plant, China. *Environ pollut (Barking, Essex : 1987)* 2015; **200**: 16-23.
331. Cesaro A, Belgiorno V, Gorrasi G, et al. A relative risk assessment of the open burning of WEEE. *Environ Sci Pollut Res Int* 2019; **26**(11): 11042-52.
332. Gerić M, Gajski G, Oreščanin V, Domijan A-M, Kollar R, Garaj-Vrhovac V. Environmental risk assessment of wastewaters from printed circuit board production: A multibiomarker approach using human cells. *Chemosphere* 2017; **168**: 1075-81.
333. Koike E, Yanagisawa R, Takigami H, Takano H. Penta- and octa-bromodiphenyl ethers promote proinflammatory protein expression in human bronchial epithelial cells in vitro. *Toxicol In Vitro* 2014; **28**(2): 327-33.
334. Lu JY, Zhu QY, Zhang XX, et al. Directly repurposing waste optical discs with prefabricated nanogrooves as a platform for investigation of cell-substrate interactions and guiding neuronal growth. *Ecotoxicol Environ Saf* 2018; **160**: 273-81.
335. Magalini F. Global challenges for e-waste management: the societal implications. *Rev Environ Health* 2016; **31**(1): 137-40.
336. McAllister L, Magee A, Hale B. Women, E-Waste, and Technological Solutions to Climate Change. *Health & Human Rights: An International Journal* 2014; **16**(1): 166-78.
337. Mogharabi M, Abdollahi M, Faramarzi MA. Toxicity of nanomaterials; an undermined issue. *Daru* 2014; **22**(1): 59-.
338. Ni K, Lu Y, Wang T, et al. A review of human exposure to polybrominated diphenyl ethers (PBDEs) in China. 2013. p. 607-23.
339. Ohajinwa CM, Van Bodegom PM, Vijver MG, Peijnenburg WJGM. Health Risks Awareness of Electronic Waste Workers in the Informal Sector in Nigeria. *Int J Environ Res Public Health* 2017; **14**(8): 911.
340. Ohajinwa CM, van Bodegom PM, Vijver MG, Peijnenburg WJGM. Impact of informal electronic waste recycling on metal concentrations in soils and dusts. *Environ Res* 2018; **164**: 385-94.
341. Perkins DN, Brune Drisse M-N, Nxele T, Sly PD. E-waste: a global hazard. *Ann Glob Health* 2014; **80**(4): 286-95.
342. Song Q, Zeng X, Li J, Duan H, Yuan W. Environmental risk assessment of CRT and PCB workshops in a mobile e-waste recycling plant. *Environ Sci Pollut Res Int* 2015; **22**(16): 12366-73.
343. Vaccari M, Vinti G, Cesaro A, et al. WEEE Treatment in Developing Countries: Environ pollut and Health Consequences-An Overview. *Int J Environ Res Public Health* 2019; **16**(9): 1595.
344. Vojta Š, Bečanová J, Melymuk L, et al. Screening for halogenated flame retardants in European consumer products, building materials and wastes. *Chemosphere* 2017; **168**: 457-66.
345. Mishra S. Perceived and Manifested Health Problems among Informal E-waste Handlers: A Scoping Review. *Indian J Occup Environ Med* 2019; **23**(1): 7-14.
346. Afonso JC. Waste Electrical and Electronic Equipment: The Anthropocene Knocks on Our Door. *Revista Virtual De Quimica* 2018; **10**(6): 1849-97.
347. Chandrakant SS. IMAPACT OF E- WASTE ON ENVIRONMENT, HUMAN HEALTH AND EMPLOYMENT- A REVIEW. *Indo Am j pharm sci* 2018; **5**(1): S115-S9.
